# First nitrosoproteomic profiling deciphers the cysteine S-nitrosylation involved in multiple metabolic pathways of tea leaves

**DOI:** 10.1038/s41598-019-54077-2

**Published:** 2019-11-26

**Authors:** Chen Qiu, Jianhao Sun, Yu Wang, Litao Sun, Hui Xie, Yiqian Ding, Wenjun Qian, Zhaotang Ding

**Affiliations:** Tea Research Institute, Qingdao Agricultural University, Qingdao, Shandong China

**Keywords:** Proteome, Protein-protein interaction networks

## Abstract

Cysteine S-nitrosylation is a reversible protein post-translational modification and critically regulates the activity, localization and stability of proteins. Tea (*Camellia sinensis* (L.) O. Kuntze) is one of the most thoroughly studied evergreen crop due to its broad non-alcoholic beverage and huge economic impact in the world. However, little is known about the S-nitrosylome in this plant. Here, we performed a global analysis of cysteine S-nitrosylation in tea leaves. In total, 228 cysteine S-nitrosylation sites were identified in 191 proteins, representing the first extensive data on the S-nitrosylome in tea plants. These S-nitrosylated proteins were located in various subcellular compartments, especially in the chloroplast and cytoplasm. Furthermore, the analysis of functional enrichment and PPI network revealed that the S-nitrosylated proteins were mainly involved in multiple metabolic pathways, including glycolysis, pyruvate metabolism, Calvin cycle and TCA cycle. Overall, this study not only systematically identified the proteins of S-nitrosylation in cysteines of tea leaves, but also laid the solid foundation for further verifying the roles of S-nitrosylation in cysteines of tea plants.

## Introduction

Tea (*Camellia sinensis* (L.) O. Kuntze), an evergreen woody plant, is one of the most popular non-alcoholic beverage crops in the world. With the completion of the tea genome sequence^1,2^, the research around the proteomics and protein modification is becoming increasingly important in tea plants. For example, the global proteomics of lysine acetylation and crotonylation in tea leaves have identified and reported in our previous research^3,4^. In recent years, S-nitrosylation, an oxidative modification of cysteine thiol by nitric oxide to form protein S-nitrosothiols, is considered as one of the most important post-translational modification^5,6^. It has diverse regulatory roles in bacteria, yeast and plants and in all mammalian cells, including alterations in conformation, stability, subcellular localization, biochemical activity and protein-protein interactions of the modified targets^7–9^.

Cysteine S-nitrosylation was found widespread in plants. For example, there were 13 S-nitrosylated proteins (SNPs) identified in *Pisum sativum* by the biotin-switch method after treating peroxisomal proteins with S-nitrosoglutathione. The S-nitrosylation levels of catalase and glycolate oxidase were down-regulated under cadmium and 2,4-D treatments, suggesting that S-nitrosylation could regulate the level of H_2_O_2_ under abiotic stress^10^. There were approximately 80 S-nitrosylated candidate proteins identified in potato leaves and tubers using a modified biotin switch assay and nano liquid chromatography combined with mass spectrometry^11^. There were 203 SNPs identified in isoprene-emitting and non-isoprene-emitting genotypes of *Populus* × *canescens* under the short, acute ozone exposure by label-free liquid chromatography-tandem mass spectrometry (LC-MS/MS) approach. And the S-nitroso-proteome of the non-isoprene-emitting genotype *in vivo* was more susceptible to ozone-induced changes compared with the isoprene-emitting plants^12^. There were 1195 endogenously S-nitrosylated peptides in 926 proteins identified in *Arabidopsis* wild type and gsnor1-3 seedlings, representing the largest dataset reported to date in plants. Those identified proteins were involved in a wide range of biological processes and significantly enriched in photosynthesis and carbohydrate metabolism^13^. However, the information about SNPs in tea plants is not yet determined.

In order to gain more insight into the cysteine S-nitrosylation of tea leaves, we performed the S-nitrosoproteomic analysis using iodo-TMT (iodo-tandem mass tag) labelling, affinity enrichment and high-resolution LC-MS/MS. In the past, studies on the S-nitrosoproteomic of plants mainly used biotin-switch-based shotgun methods and mass spectrometry-based site-specific S-nitrosoproteomic analysis^14–16^. However, due to various technical limitations, the number of SNPs identified by shotgun proteomics was usually small^16^. The site-specific S-nitrosoproteomic analysis enormously enhanced the ability to identify SNPs^17^. This is the first time to identify the plant S-nitrosoproteomic using iodo-TMT labelling site-specific S-nitrosoproteomic. In the aggregate, 228 S-nitrosylation sites (SNSs) were identified in 191 proteins. Our research provided the first comprehensive view of the S-nitrosylome in tea plants.

## Materials and Methods

### Plant material and growth conditions

The 1-year-old seedlings of the tea plant cultivar ‘QN 3’ were used which was planted in pots in the phytotron. The parameters of phytotron were set as follows: temperature, 25 °C/ 20 °C (12 h light/12 h dark); humidity, 75%; and light intensity, 18000 Lx. Plants were irrigated with nutrient solution (Plant- Prod 20- 20- 20 Fertilizer, Lambrou Agro, Cyprus) every 3 days. The third and/or fourth mature leaves from the terminal bud were collected after two weeks. The samples were quickly frozen in liquid nitrogen and stored at −80 °C for further study. Six biological replicates were tested.

### Protein extraction

1 g sample was taken out from −80 °C and grinded in pre-cooled mortar by liquid nitrogen into cell powder. Then 0.5 g cell powder was transferred to a 5-mL centrifuge tube. After that, 2 mL of the phenol extraction buffer (containing 20 mM Methyl methanethiosulfonate (MMTS) (Sigma), 1% protein inhibitor (Calbiochem) and 2 mM EDTA(Sigma)) was added to centrifuge tube, followed by sonication, and then incubation at room temperature for 30 min to block free cysteine thiols. An equal volume of Tris equilibrium phenol was added and centrifuged at 5500 g at 4 °C for 10 min. Taking 0.8 mL supernatant and adding 4 mL of 0.1 M ammonium acetate/methanol to precipitate overnight, and the protein pellet was washed with methanol and acetone, respectively. Re-dissolving the pellet in HES Buffer (50 mM HEPES pH 8.0, 1 mM EDTA, 0.1% SDS (Sigma)) and determining the protein concentration using BCA kit (Beyotime Biotechnology).

### Iodo-TMT labeling

A 1 mg solution was taken and the volume was made up with HES Buffer, the protein (2 μg/μL) was labeled according to the instructions of Iodo-TMT kit (90102 Thermo scientific). The simple operation is as follows: the labeled reagent was thawed, mixed with protein through vortex, added a final concentration of 20 mM sodium ascorbate (Sigma), the reaction solution was incubated at 37 °C for 1 h in the dark, add a final concentration of 20 mM DTT (Sigma) and the reaction solution was incubated at 37 °C for 15 min in the dark to stop the reaction. The labeled protein was precipitated with 6 times pre-cooled acetone to remove excess reagents, and then washed with acetone for 2 times. The protein was broken up to treated with 100 mM ammonium bicarbonate (Sigma) for enzymatic hydrolysis.

### Trypsin digestion

Trypsin (Promega) was added to the protein solution at a mass ratio of 1:50 (trypsin: protein) and digested overnight at 37 °C. For digestion, the protein solution was reduced with 5 mM dithiothreitol at 56 °C for 30 min and alkylated with 11 mM iodoacetamide (Sigma) at room temperature for 15 min in darkness. Trypsin was added at a mass ratio of 1:100 (trypsin: protein) for 4 h-digestion. After the trypsin-digested peptides were desalted, the same amounts of peptides were mixed and vacuum-dried.

### HPLC fractionation

The peptides were fractionated by high pH reverse-phase HPLC using Thermo Betasil C18 column. The specification and dimension of C18 column were 5 μm and 4.6 × 250 mm, respectively. The flow rate was 1 mL/min. In brief, the fractional gradient of the peptide was 8–32% acetonitrile (Fisher Chemical, pH 9.0), 60 fractions were separated in 60 min. Afterwards, the peptides were synthesized into 4 fractions, centrifuged and dried in vacuo.

### Affinity enrichment

Wash the Anti-TMT Resin (Prod#90076, ThermoFisher Scientific Waltham USA) three times with one column volume of 1X TBS. Resuspend lyophilized peptides with 100 μL of 1X TBS. Save a small portion of this unfractionated sample for direct analysis of the non-enriched samples. Add peptides to the Anti-TMT Resin and incubate for 2 hours at room temperature or overnight with end-over-end mixing at 4 °C. Remove the supernatant and wash the resin five times (5 minutes/wash) with one column volume of TBS. Note: Addition of 2 M urea or MS-compatible detergents (0.05–0.2%) to TBS wash buffers can be used to decrease nonspecific peptide binding. Wash the resin three times with one column volume of 1X TBS. Wash the resin three times with one column volume of water. Elute the sample with four column volumes of TMT Elution Buffer (50% acetonitrile, 0.4% trifluoroacetic acid (Sigma-Aldrich)). Pool the eluate, freeze peptides and lyophilize using a vacuum concentrator. Resuspend the samples in 25 μL of 5% acetonitrile/0.1% formic acid and inject 1–5 μL directly onto an LC-MS/MS system.

### LC-MS/MS analysis

The peptide was dissolved in A phase (0.1% formic acid in 2% acetonitrile) and separated using an EASY-nLC 1000 system. The gradient was comprised of an increase from 9% to 23% solvent B (0.1% formic acid (Fluka) in 90% acetonitrile) over 20 min, 23% to 35% in 13 min and climbing to 80% in 4 min then holding at 80% for the last 3 min, all at a constant flow rate of 700 nL/min on an EASY-nLC 1000 UPLC system.

The peptides were separated by ultra-high performance liquid phase system and injected into an NSI ion source for ionization. After that, the sample was proceeded to MS/MS of Q Exactive^TM^ Plus (Thermo) for analysis. The primary mass spectrometer scan range was 350–1800 m/z and the scan resolution was set to 70000; the tandem mass spectrometer scan range has a fixed starting point of 100 m/z and the Orbitrap scan resolution was set to 17500. The electrospray voltage was set to 2.0 kV. A data-dependent procedure that alternated between one MS scan followed by 20 MS/MS scans with 15.0 s dynamic exclusion. Automatic gain control was set at 5E4.

### Database search

The resulting MS/MS data were processed using Maxquant search engine (v.1.5.2.8). Tandem mass spectra were searched against *Camellia sinensis* database (36,951 sequences, http://www.plantkingdomgdb.com/tea_tree/) concatenated with reverse decoy database. The minimum length of the peptide was set to 7 amino acid residues and the maximum number of peptides was set to 5. Trypsin/P was specified as cleavage enzyme allowing up to 4 missing cleavages. The mass tolerance for precursor ions was set to 20 ppm in First search range, 5 ppm in Main search and 0.02 Da for fragment ions. The variable modification was set to oxidation of methionine, acetylation of the N-terminus of the protein, Iodo TMT-6plex var. The false discovery rate of protein identification and peptide spectrum match identification was set to 1%.

### Bioinformatics analysis

The secondary structures of proteins were predicted by NetSurfP. The GO annotation and enrichment analyses were done by UniProt-GOA database (http://www.ebi.ac.uk/GOA/). The subcellular localization was performed by wolfpsort (http://www.genscript.com/wolf-psort.html). The pathway of proteins was annotated by KEGG database. The functional domain of proteins was annotated by InterPro (http://www.ebi.ac.uk/interpro/scan.html) databases. The protein-protein interaction (PPI) network analysis was performed by Cytoscape software, and the PPI network was obtained from the STRING database version 10.5 (http://string-db.org/). A graph theoretical clustering algorithm, MCODE, was utilized to analyze densely connected regions.

## Results

### Detection of cysteine SNPs and SNSs

**Figure 1:**
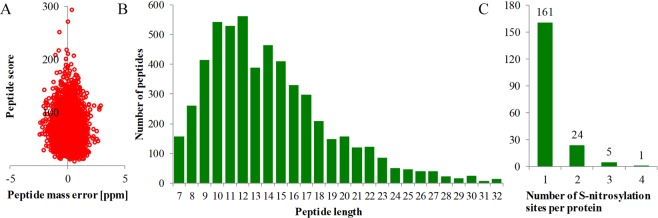
Quality Control validation of LC-MS/MS data. (**A)** Peptide score of all S-nitrosylated peptides is plotted as a function of calibrated peptide mass errors measured from all identified peptides in parts per million (ppm). (**B**) Length distribution of the identified peptides. **(C)** Distribution of SNSs in proteins.

To characterize the global S-nitrosylation proteome of tea leaves, we performed the nitrosoproteomic analysis using iodo-TMT labelling, affinity enrichment and high-resolution LC-MS/MS. Altogether, 228 unique cysteine SNSs in 191 SNPs were identified, representing the rich data on the S-nitrosylome of tea leaves. MS data were available from the Proteome Xchange database (Project accession: PXD012443). The distribution of the mass error was near zero and most values were less than 5 ppm (Fig. 1A), which fitted the requirement of the mass accuracy of MS data. Furthermore, most peptides were ranged from 8 to 20 amino acids (Fig. 1B), which was in agreement with the property of tryptic peptides, and thus sample preparation met the technically standard. Of all the 191 SNPs, about 84.3% (161) of them contained only one S-nitrosylation site (SNS), whereas 15.7% (30) contained multiple SNSs (Fig. 1C). Only one protein, glutamate-glyoxylate aminotransferase (GGAT), had the most extensive SNSs (C226, C377, C382, C417 in CSA000918.1). Detailed information about all identified SNPs can be found in Supplementary Table 1.

### Pattern analysis of SNSs

**Figure 2:**
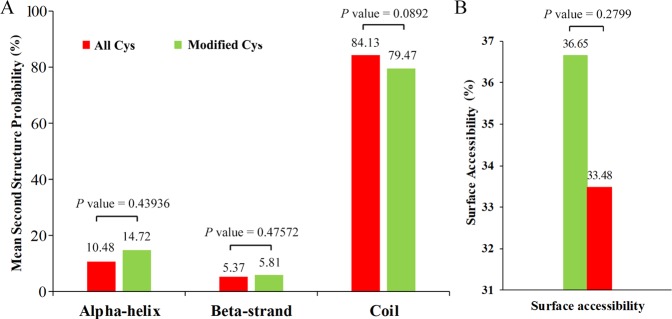
Predicted protein secondary structures near SNSs. (**A)** Probabilities for different secondary structures (alpha-helix, beta-strand and coil) of modified cysteine residues were compared with the secondary structure probabilities of all cysteine residues. **(B)** Predicted surface accessibility of SNSs. All cysteine sites were marked in green and S-nitrosylated cysteine sites were marked in red.

To understand the local secondary structures of protein sequences surrounding the SNSs in more details, the NetSurfP software was used. The results in Fig. 2A: 20.53% of the S-nitrosylated cysteines were located in the regions of ordered secondary structure. Thereinto, 14.72% sites were located in alpha-helices and 5.81% sites in beta-strands. The remaining sites were located in disordered regions. Nevertheless, it seems that there was no tendency towards S-nitrosylation in tea leaves based on the similarity of distribution patterns between the S-nitrosylated cysteines and all cysteines. The surface accessibility of the S-nitrosylated cysteine sites was also assessed. As shown in Fig. 2B, 33.48% of the SNSs were exposed to the protein surface, compared with 36.65% of all cysteine residues (Supplementary Table 2). Therefore, cysteine nitroso group might have a slight change in the surface property of the proteins.

### Functional classification and subcellular localization of SNPs

**Figure 3:**
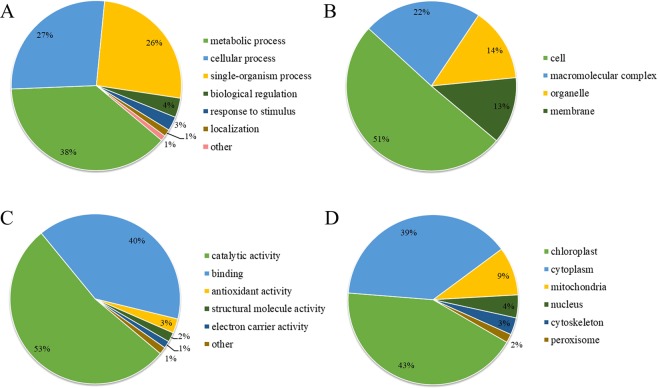
Functional classification and subcellular localization of SNPs. (**A)** Biological process, **(B)** Cellular component, **(C)** Molecular function and **(D)** Subcellular localization.

To better elucidate the potential roles of SNPs in tea leaves, we performed the GO functional classification of identified SNPs with UniProt-GOA database (Supplementary Table 3). According to the analysis of biological processes, major SNPs were involved in metabolic process (38%) and cellular process (27%) (Fig. 3A). For the analysis of cellular component, a large number of the modified proteins were distributed within the cell (51%), macromolecular complex (23%), organelle (14%) and membrane (12%) (Fig. 3B). In the analysis of molecular functions, over half of the SNPs were classified into catalytic activity-related proteins (53%) and less than 40 percent were classified into binding proteins (39%) (Fig. 3C). The results showed that the functions of SNPs mainly involved in catalytic activity, cell component and metabolic process.

We also investigated the subcellular localization of the SNPs. As shown in Fig. 3D, 79 of the identified SNPs were distributed in the chloroplast (41%), 71 in the cytoplasm (37%), and 17 in the mitochondrion (9%). The data showed that the highest proportion of SNPs was located in chloroplast of tea leaves.

### GO, KEGG enrichment and protein domain analysis of SNPs

**Figure 4:**
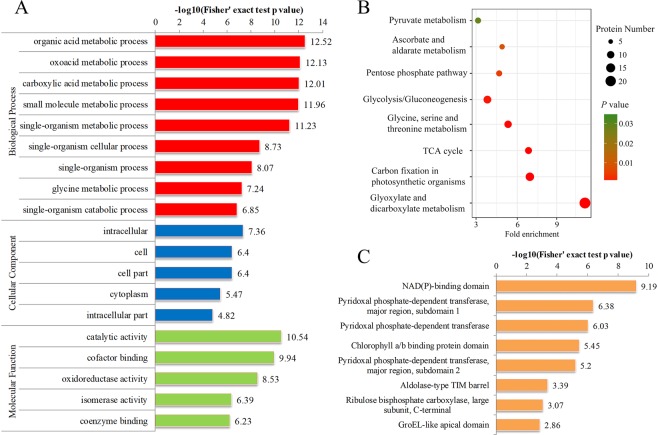
The GO, KEGG enrichment analysis and protein domain of SNPs. (**A)** GO enrichment analysis of SNPs. **(B)** KEGG enrichment analysis of SNPs. **(C)** Domain enrichment analysis of SNPs. The horizontal axis is −log10 (Fisher’s exact test p value).

To estimate the nature of SNPs in tea leaves, we performed GO and KEGG pathway (Supplementary Table 4). As for GO enrichment, 191 SNPs were significantly enriched in biological process, cellular component and molecular function. In biological process, the SNPs were significantly enriched in ‘organic acid metabolic process’, ‘oxoacid metabolic process’ and ‘carboxylic acid metabolic process’. Meanwhile, significant enrichments of SNPs involved in ‘intracellular’, ‘cell’ and ‘cytoplasm’ were observed in the cellular component. In agreement with these observations, many modified proteins were found to be associated with enzymatic and binding activities in molecular function analysis (Fig. 4A). According to the analysis of KEGG enrichment, 10 significantly enriched pathways were identified, including carbon fixation in photosynthetic organisms, glycolysis, pyruvate metabolism and tricarboxylic acid (TCA) cycle (Fig. 4B).

We also performed protein domain analysis of SNPs (Supplementary Table 4). The results showed that SNPs were mostly enriched in NAD(P)-binding domain, pyridoxal phosphate-dependent transferase and chlorophyll a/b binding protein domain (Fig. 4C).

### Cysteine S-nitrosylation in various key enzymes of metabolic pathways

**Figure 5:**
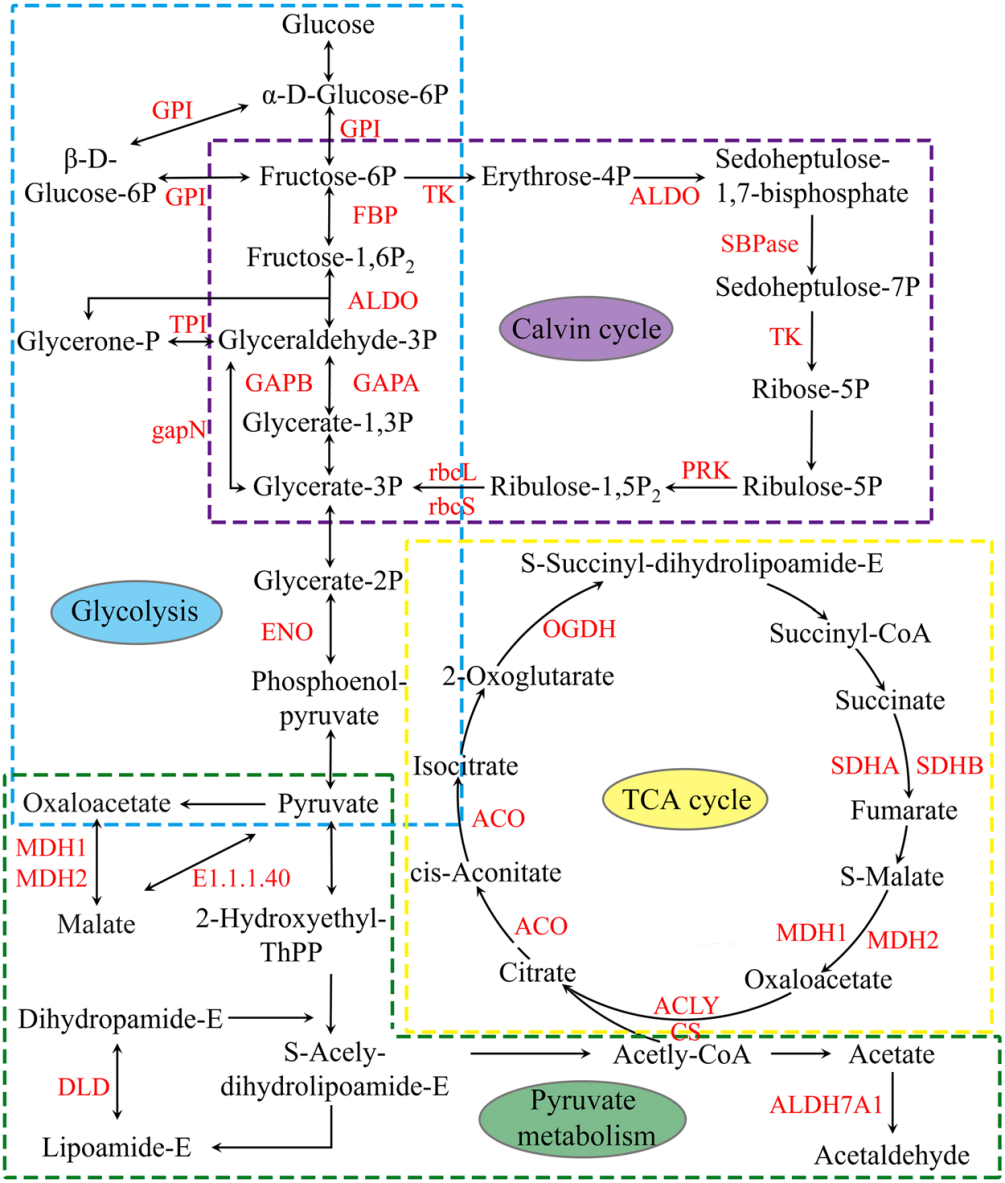
S-nitrosylated enzymes involved in major metabolic pathways (glycolysis, pyruvate metabolism, TCA cycle, Calvin cycle). The SNPs are marked in red.

The analysis of S-nitrosylome in KEGG pathway showed that many SNPs were involved in multiple metabolic pathways, such as glycolysis, pyruvate metabolism, TCA cycle and Calvin cycle (Fig. 5).

In total, eight glycolytic enzymes were identified as SNPs, including glucose-6-phosphate isomerase (GPI), fructose-1,6-bisphosphatase (FBP), fructose-bisphosphate aldolase (ALDO), triosephosphate isomerase (TPI), glyceraldehyde-3-phosphate dehydrogenase A (GAPA), glyceraldehyde-3-phosphate dehydrogenase B (GAPB), NADP-dependent glyceraldehyde-3-phosphate dehydrogenase (gapN) and enolase (ENO). The LC-MS/MS spectra of three representative S-nitrosyl-peptides (GPI, GAPA and GPAB) in glycolysis were shown in Supplementary Figs. 1–3. Seven key enzymes in pyruvate metabolism were S-nitrosylated, including malate dehydrogenase 1 (MDH1), malate dehydrogenase 2 (MDH2), dihydrolipoamide dehydrogenase (DLD), aldehyde dehydrogenase family 7 member A1 (ALDH7A1), lactoylglutathione lyase (GLO1), malate dehydrogenase [NADP] (E1.1.1.82) and NADP-dependent malic enzyme (E1.1.1.40). The LC-MS/MS spectra of two representative S-nitrosyl-peptides (MDH1 and MDH2) in pyruvate metabolism were shown in Supplementary Figs. 4 and 5.

There were 9 SNPs identified in TCA cycle, including MDH1, MDH2, succinate dehydrogenase (ubiquinone) flavoprotein subunit (SDHA), succinate dehydrogenase (ubiquinone) iron-sulfur subunit (SDHB), DLD, 2-oxoglutarate dehydrogenase E1 component (OGDH), aconitate hydratase (ACO), citrate synthase (CS) and ATP-citrate synthase beta chain protein 1 (ACLY). The LC-MS/MS spectra of three representative S-nitrosyl-peptides (CS, SDHA and SDHB) in TCA cycle were shown in Supplementary Figs. 6–8. Ten metabolic enzymes involved in Calvin cycle were found to be S-nitrosylated, including ribulose-bisphosphate carboxylase large chain (rbcL), ribulose-bisphosphate carboxylase small chain (rbcS), GAPA, GAPB, ALDO, TPI, FBP, transketolase (TK), sedoheptulose-bisphosphatase (SBPase) and phosphoribulokinase (PRK). The LC-MS/MS spectra of three representative S-nitrosyl-peptides (rbcS, rbcL and ALDO) in Calvin cycle were shown in Supplementary Figs. 9–11.

### Protein-protein interaction analysis of SNPs

**Figure 6:**
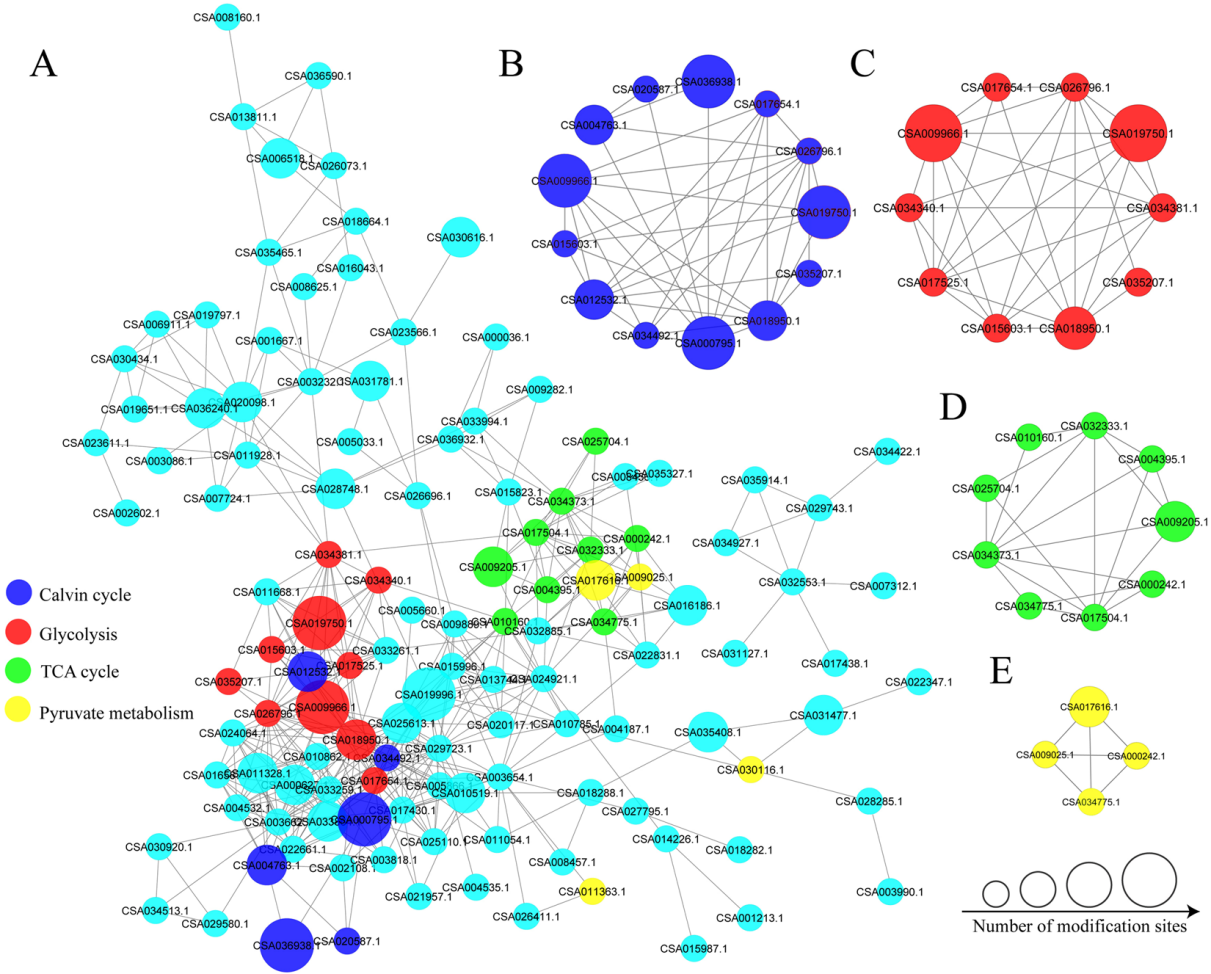
PPI network of SNPs. (**A)** The whole PPI networks. Interaction network of SNPs involved in **(B)** Calvin cycle, **(C)** glycolysis, **(D)** TCA cycle and **(E)** pyruvate metabolism.

To further understand the cellular processes regulated by S-nitrosylation in tea leaves, we analyzed the PPI among the identified 191 SNPs using the STRING database and Cytoscape software. A total of 124 SNPs identified as nodes connected with each other, which represented a global PPI network of tea leaves (Fig. 6A, Supplementary Table 6). Thereinto, seven proteins displayed the highest degree (≥20), including GAPA, GAPB, FBP, PRK, ALDO, glycine cleavage system H protein (gcvH) and glycine hydroxymethyltransferase (glyA). Eight clusters of highly enriched interaction were extracted from the entire interaction network by MCODE. Notably, the S-nitrosylated enzymes of Calvin cycle, glycolysis, TCA cycle and pyruvate metabolism were highly enriched, which was consistent with the analysis of KEGG pathways (Fig. 6B–E).

## Discussion

In this research, 228 SNSs were identified in 191 proteins through a combination of TMT labeling and high-resolution LC−MS/MS in tea leaves. In order to make the data more accurate, six biological replicates were performed. However, compared to previous S-nitrosoproteomic study in model plant *Arabidopsis*^13^, the number of SNPs we identified was small. The labile and dynamic nature of protein S-nitrosylation may be the major causes of the smaller number of SNPs identified in tea leaves. In addition, we have discovered many new SNPs which were not identified in the S-nitrosoproteomic of *Arabidopsis*. For example, in glycolysis and Calvin cycle, GPI, FBP and TK were found to be S-nitrosylated in tea leaves. In pyruvate metabolism and TCA cycle, MDH2, DLD, ALDH7B4, GLO1, E1.1.1.40, E1.1.1.82, ACLY and SDHB were found to be S-nitrosylated in tea leaves.

To check into the SNPs in various metabolic processes, we analyzed the SNPs in glycolysis, Calvin cycle, pyruvate metabolism and TCA cycle. In glycolysis, there were 8 enzymes modified by S-nitrosylation in tea leaves, including GAPA, GAPB and GPI. GAPDH (EC 1.2.1.13) catalyzes the conversion of glyceraldehyde-3P to glycerate-1,3P_2_. Two GAPDH proteins (GAPA and GAPB) were found to be S-nitrosylated in our study. An increased in GAPDH acetylation led to the increased activity in glycolysis^18^. GPI (EC:5.3.1.9) is a multifunctional protein that catalyzes the reversible reaction between glucose-6P and fructose-6P in glycolysis, and it also has other important physiological and biochemical functions. It was reported that GPI was essential for extracellular polysaccharide biosynthesis and DSF signals production^19^. In this research, one SNS (C8 in CSA017525.1) was identified in GPI. From the above, we speculated that cysteine S-nitrosylation might play an important role in glycolysis.

In Calvin cycle, there were 10 enzymes modified by S-nitrosylation in tea leaves, including rbcL, rbcS and ALDO. RuBisco (EC:4.1.1.39) is the major protein in the stroma of chloroplasts, which catalyzes the carboxylation of ribulose-1,5-bisphosphate^20^. Two RuBisco proteins (rbcL and rbcS) were found to be S-nitrosylated in our study. In *Brassica juncea*, RuBisCO inactivation mediated by S-nitrosylation could regulate cellular detoxification in cold stress^21^. In tea leaves, RuBisCO was S-nitrosylated at 6 cysteine sites (C97 in CSA020587.1, C130 and C143 in CSA004763.1, C207, C262 and C299 in CSA036938.1). It is speculated that the function of RuBisCO might be regulated by S-nitrosylation. ALDO (EC 4.1.2.13) is a key enzyme which catalyzes the reversible reaction, such as splitting the fructose 1,6-bisphosphate into the glyceraldehyde 3-phosphate, and splitting the erythrose-4P into the sedoheptulose 1,7-bisphosphate. ALDO has two isoforms, including cytosolic ALDO and chloroplastic ALDO^22^. The cytosolic ALDO was involved in the glycolysis and gluconeogenesis pathways; while the chloroplastic ALDO was involved in the Calvin cycle^23–25^. In *Arabidopsis thaliana*, S-nitrosylation of cytosolic ALDO negatively regulated its enzymatic activity^26^. In this research, two ALDO modified by S-nitrosylation (C78, C162, C276 in CSA009966.1 and C246 in CSA035207.1) were identified in cytoplasm and chloroplast, suggesting that the S-nitrosylated-ALDO might be important for the Calvin cycle and glycolysis of tea leaves.

In TCA cycle, there were 9 enzymes modified by S-nitrosylation in tea leaves, including CS, SDHA and SDHB. CS (E.C. 2.3.3.1) catalyzes the condensation reaction of the two-carbon acetate residue from acetyl coenzyme A and a molecule of four-carbon oxaloacetate to form the six-carbon citrate in the first step of the TCA cycle. In *Arabidopsis*, the oxidation inhibited the activity of CS by the formation of mixed disulfides^27^. Our previous study showed that five acetylated sites were identified in CS^3^. In this study, one SNS (C438 in CSA017504.1) was identified in CS. For SDH (E.C. 1.3.5.1), it is the only enzyme that participates in both the TCA cycle and the electron transport chain^28^. In step 6 of the TCA cycle, SDH catalyzes the oxidation of succinate to fumarate with the reduction of ubiquinone to ubiquinol. So far, the function of S-nitrosylation on SDH in plants has not been explored. In this research, two subunits of SDH, SDHA and SDHB, were S-nitrosylated. Two SNSs (C179 and C309 in CSA009205.1) in SDHA and one SNS (C74 in CSA004395.1) in SDHB were identified in tea leaves. Our results could provide references for exploring the functions of related proteins in TCA cycle.

In pyruvate metabolism, MDH (EC:1.1.1.37) is a homodimeric enzyme which catalyzes the conversion of malate to oxaloacetate, coupled to NAD reduction^29^. The S-nitrosylation of MDH exhibited increased upon ozone exposure in *Populus* × *canescens*^12^. In tea leaves, two subunits of it, MDH1 (C125 in CSA000242.1) and MDH2 (C516 in CSA034775.1), were S-nitrosylated in our study, indicating a potential function of S-nitrosylation in the regulation of enzyme activities in pyruvate metabolism.

In summary, we presented the first global S-nitrosylome of tea plants by iodo-TMT labeling qualitative proteomics. The research greatly expanded our understanding of the plant S-nitrosylome and provided a basic resource for functional validation of SNPs in tea plants. Although these findings suggested an important potential role of cysteine S-nitrosylation in the multiple metabolic pathways of tea, additional experiments would be needed to conclusively prove it.

## Supplementary information

Supplementary information

## Data Availability

The datasets generated during the current study are available in the [ProteomeXchange] repository, [http://www.ebi.ac.uk/pride].
