# Supplementary material for: First nitrosoproteomic profiling deciphers the cysteine S-nitrosylation involved in multiple metabolic pathways of tea leaves
Source: Sci Rep. 2019 Nov 26;9:17525. doi: 10.1038/s41598-019-54077-2 (PMC6879589; doi:10.1038/s41598-019-54077-2)
Supplement: Supplementary file 1 — Supplementary information [file 41598_2019_54077_MOESM1_ESM.pdf]

First nitrosoproteomic profiling deciphers the cysteine S-nitrosylation involved in multiple metabolic pathways of tea leaves

Chen Qiu[1#], Jianhao Sun[1#], Yu Wang[1], Litao Sun[1], Hui Xie[1], Yiqian Ding[1], Wenjun Qian[1], Zhaotang Ding[1*]

1 Tea Research Institute, Qingdao Agricultural University, Qingdao, Shandong, China.

# Equal contributors

* Corresponding author

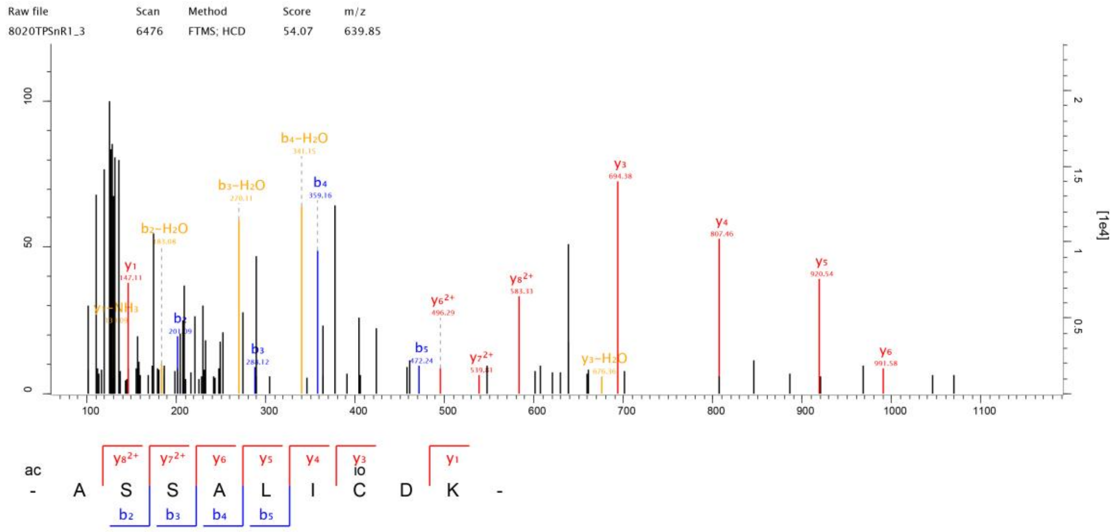

Fig S1: Representative MS/MS spectra of nitrosyl peptides from glycolysis related enzyme GPI (CSA017525.1). S-nitrosyl-peptide _ASSALIC(io)DK_ with S-nitrosylation site at C8 related enzyme GPI.

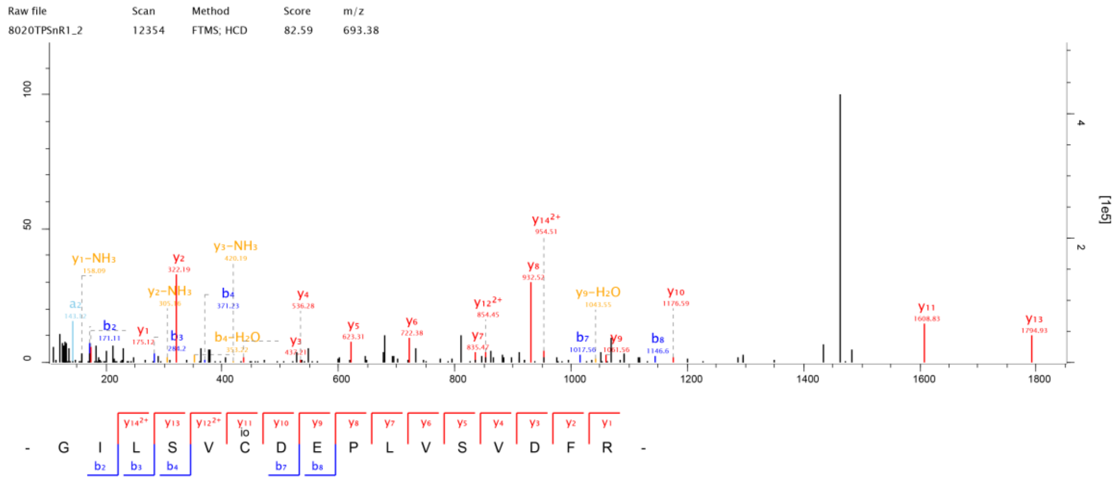

Fig S2: Representative MS/MS spectra of nitrosyl peptides from glycolysis related enzyme GAPA (CSA026796.1). S-nitrosyl-peptide _GILSVC(io)DEPLVSVDFR_ with S-nitrosylation site at C346 related enzyme GAPA.

a.

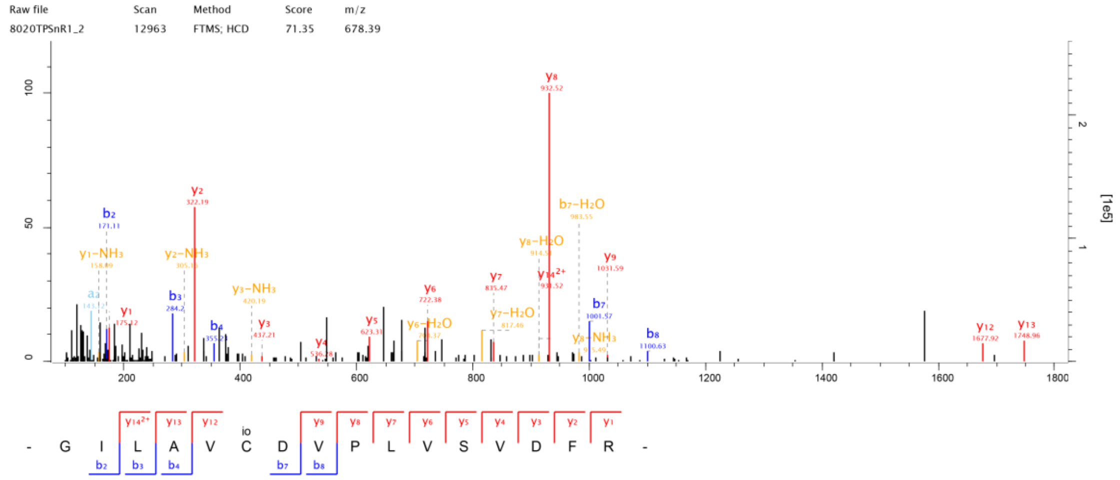

b.

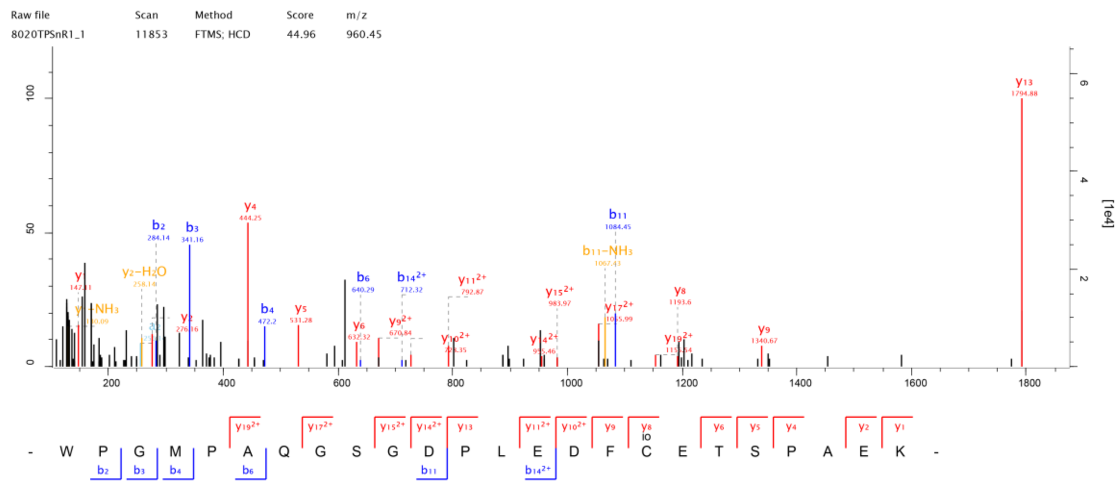

Fig S3: Representative MS/MS spectra of nitrosyl peptides from glycolysis related enzyme GAPB (CSA018950.1). (a) S-nitrosyl-peptide _GILAVC(io)DVPLVSVDFR_ with S-nitrosylation site at C367 related enzyme GAPB.

(b) S-nitrosyl-peptide _WPGMPAQGSGDPLEDFC(io)ETSPAEK_ with S-nitrosylation site at C441 related enzyme GAPB.

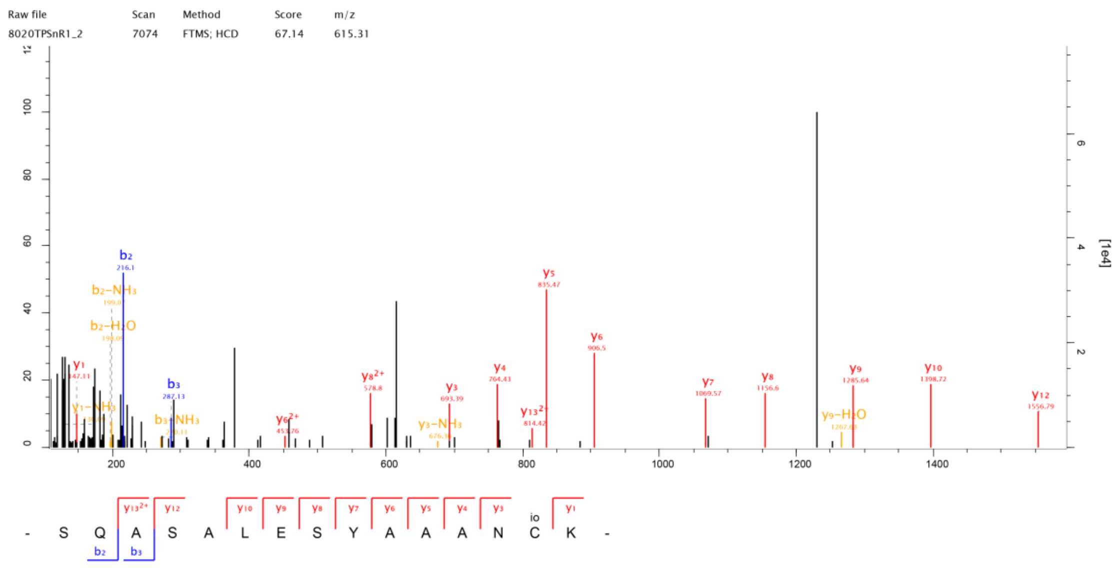

Fig S4: Representative MS/MS spectra of nitrosyl peptides from pyruvate metabolism related enzyme MDH1 (CSA000242.1). S-nitrosyl-peptide _SQASALESYAAANC(io)K_ with S-nitrosylation site at C125 related enzyme MDH1.

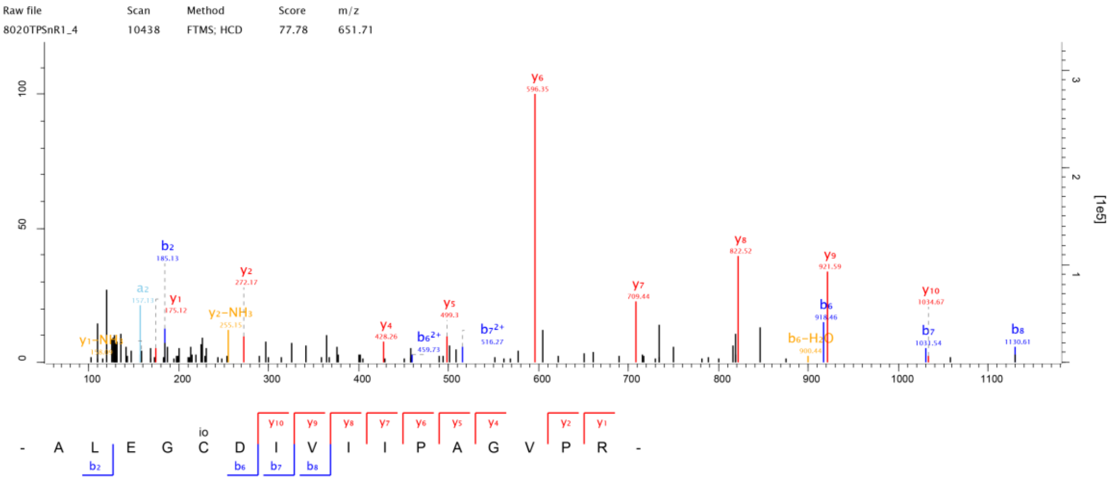

Fig S5: Representative MS/MS spectra of nitrosyl peptides from pyruvate metabolism related enzyme MDH2 (CSA034775.1). S-nitrosyl-peptide _ALEGC(io)DIVIIPAGVPR_ with S-nitrosylation site at C516 related enzyme MDH2.

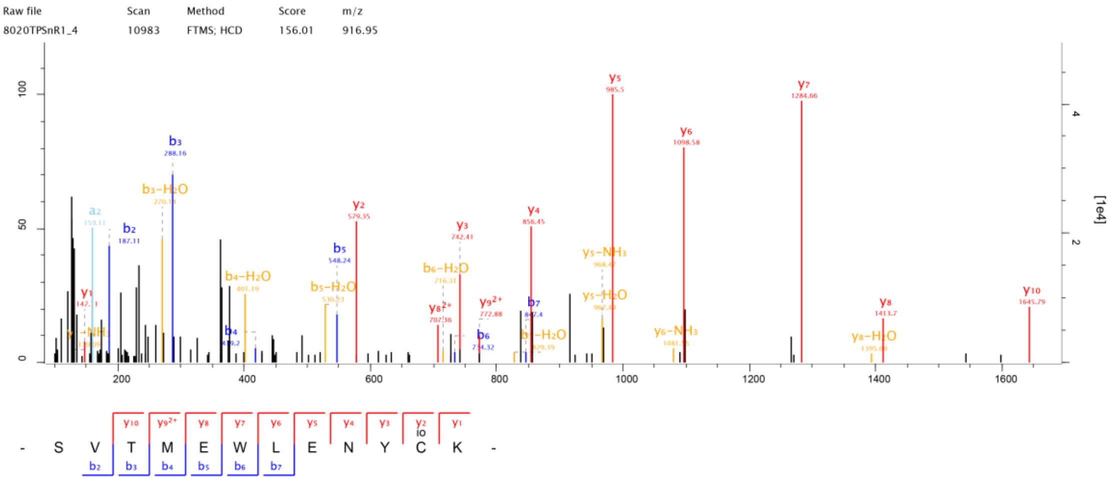

Fig S6: Representative MS/MS spectra of nitrosyl peptides from TCA cycle related enzyme CS (CSA017504.1). S-nitrosyl-peptide _SVTMEWLENYC(io)K_ with S-nitrosylation site at C438 related enzyme CS.

a.

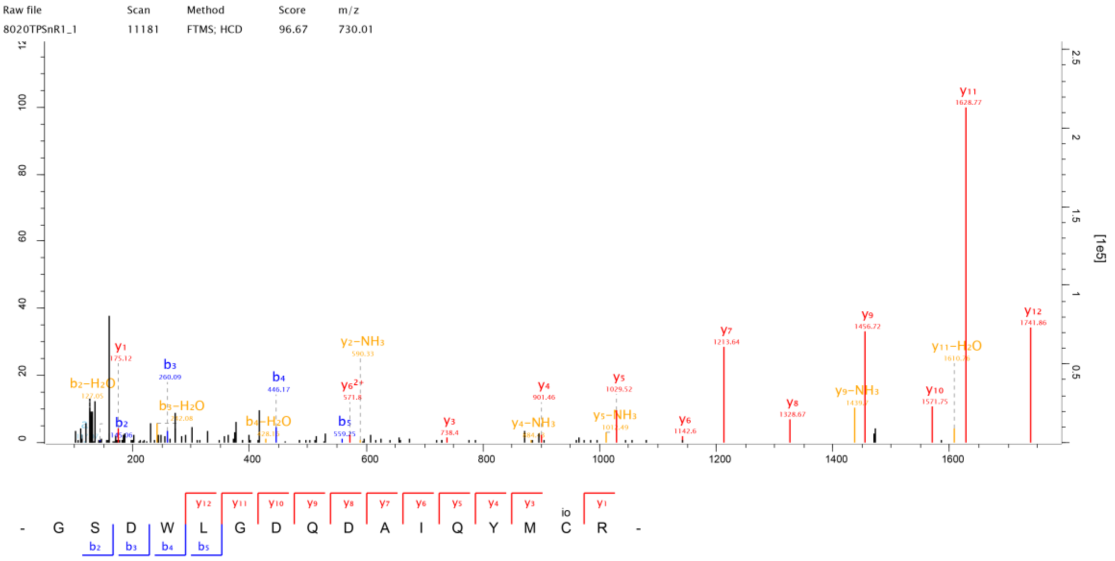

b.

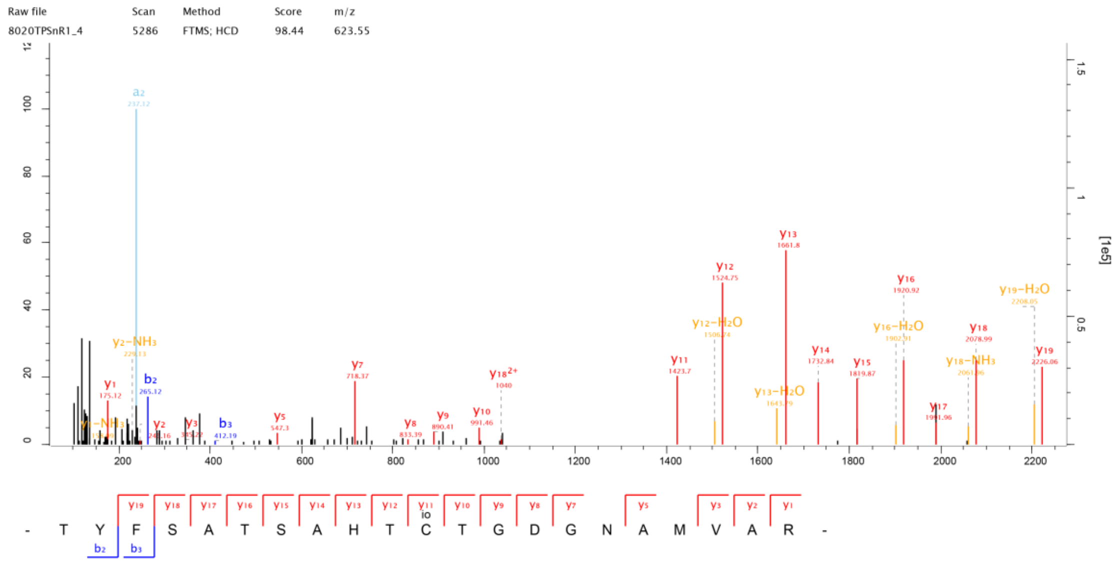

Fig S7: Representative MS/MS spectra of nitrosyl peptides from TCA cycle related enzyme SDHA (CSA009205.1). (a) S-nitrosyl-peptide _GSDWLGDQDAIQYMC(io)R_ with S-nitrosylation site at C179 related enzyme SDHA. (b) S-nitrosyl-peptide _TYFSATSAHTC(io)TGDGNAMVAR_ with S-nitrosylation site at C309 related enzyme SDHA.

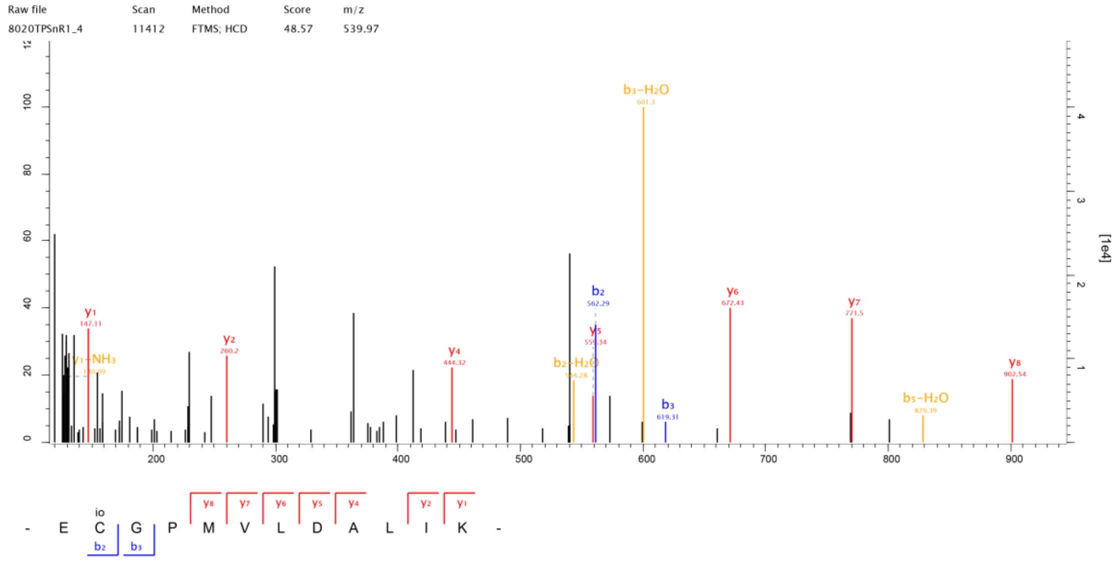

Fig S8: Representative MS/MS spectra of nitrosyl peptides from TCA cycle related enzyme SDHB (CSA004395.1). S-nitrosyl-peptide _EC(io)GPMVLDALIK_ with S-nitrosylation site at C74 related enzyme SDHB.

a.

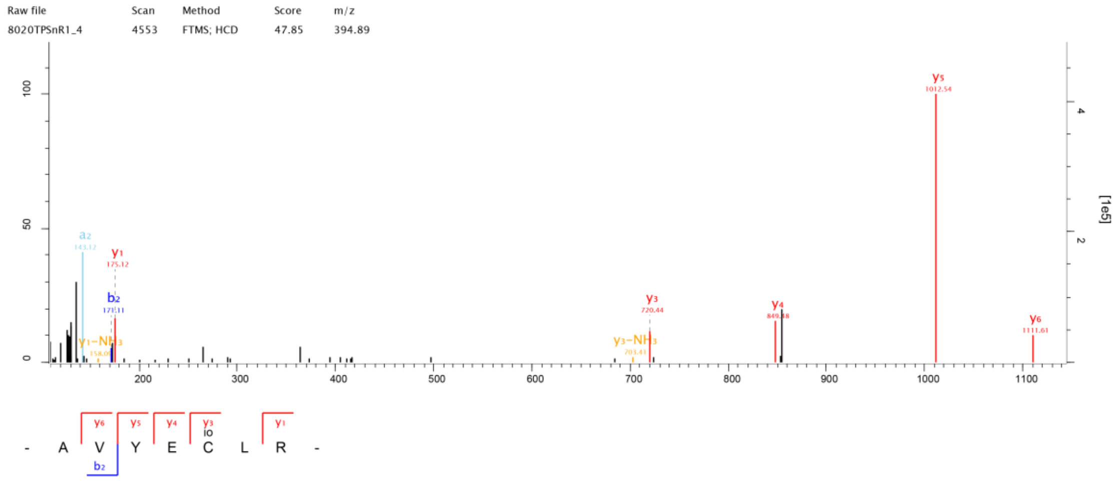

b.

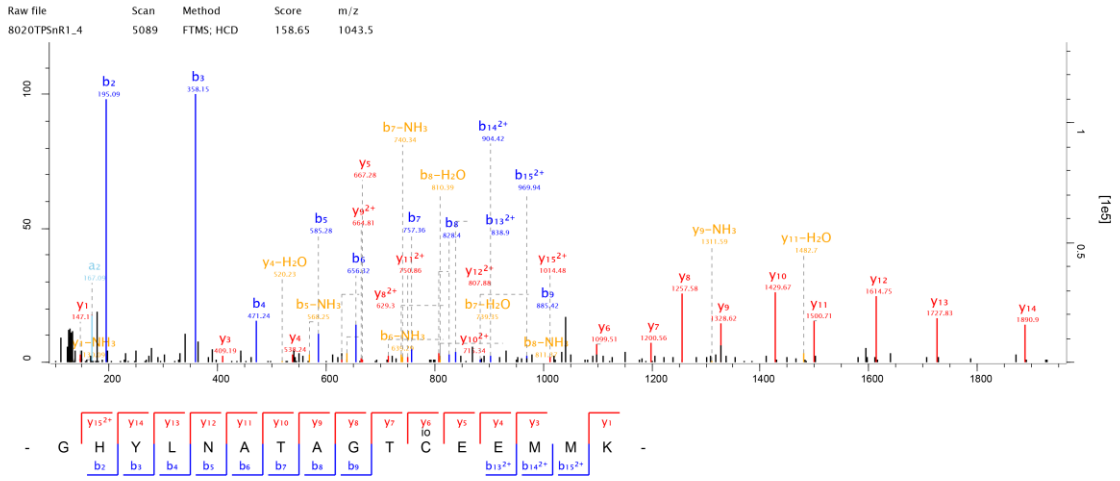

c.

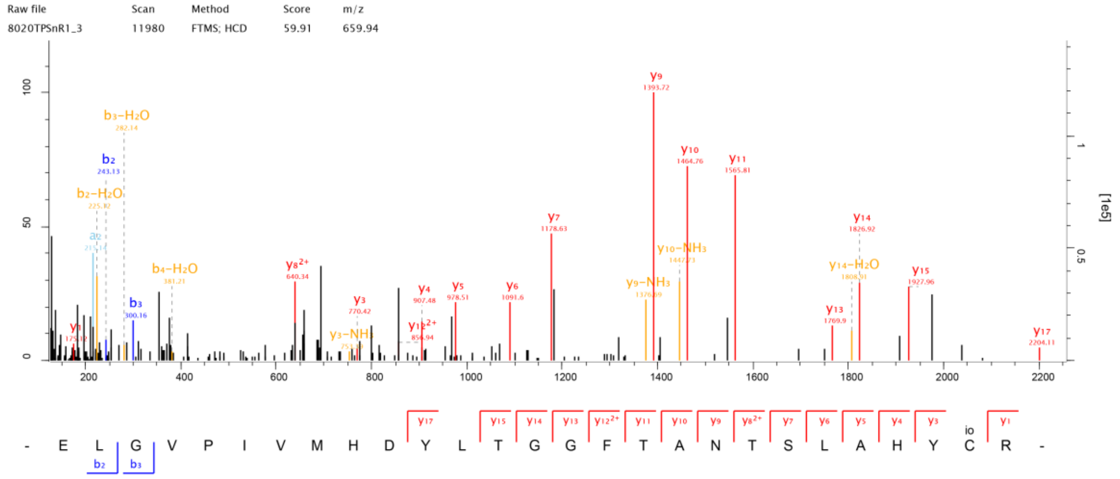

Fig S9: Representative MS/MS spectra of nitrosyl peptides from Calvin cycle related enzyme rbcL (CSA036938.1). (a) S-nitrosyl-peptide _AVYEC(io)LR_ with S-nitrosylation site at C207 related enzyme rbcL. (b) S-nitrosyl-peptide _GHYLNATAGTC(io)EEMMK_ with S-nitrosylation site at C262 related enzyme rbcL.

(c) S-nitrosyl-peptide _ELGVPIVMHDYLTGGFTANTSLAHYC(io)R_ with S-nitrosylation site at C299 related enzyme rbcL.

a.

b.

c.

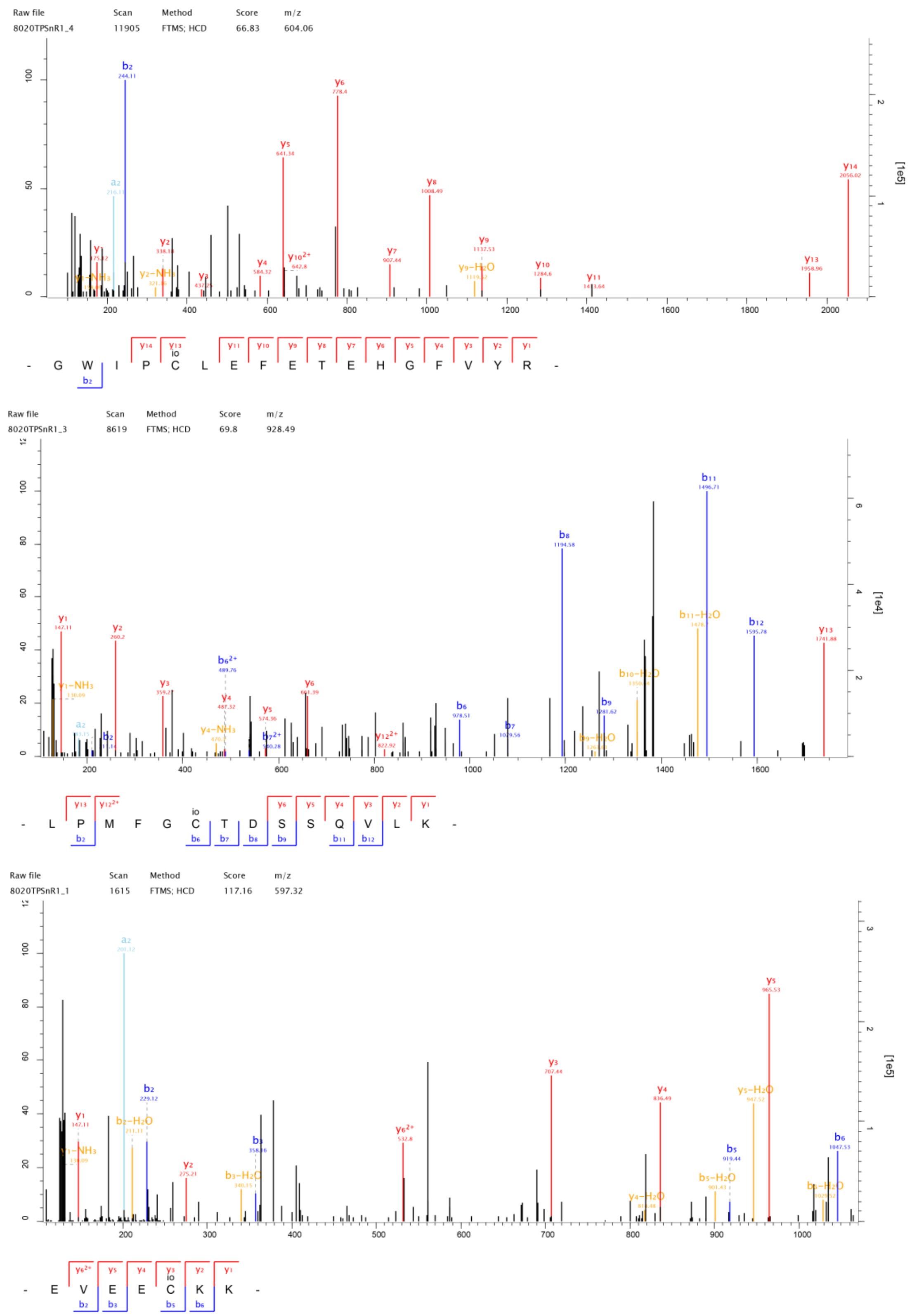

Fig S10: Representative MS/MS spectra of nitrosyl peptides from Calvin cycle related enzyme rbcS. (a) S-nitrosyl-peptide _GWIPC(io)LEFETEHGFVYR_ with S-nitrosylation site at C97 related enzyme rbcS (CSA020587.1). (b) S-nitrosyl-peptide _LPMFGC(io)TDSSQVLK_ with S-nitrosylation site at C130 related enzyme rbcS (CSA004763.1). (c) S-nitrosyl-peptide _EVEEC(io)KK_ with S-nitrosylation site at C143 related enzyme rbcS (CSA004763.1).

a.

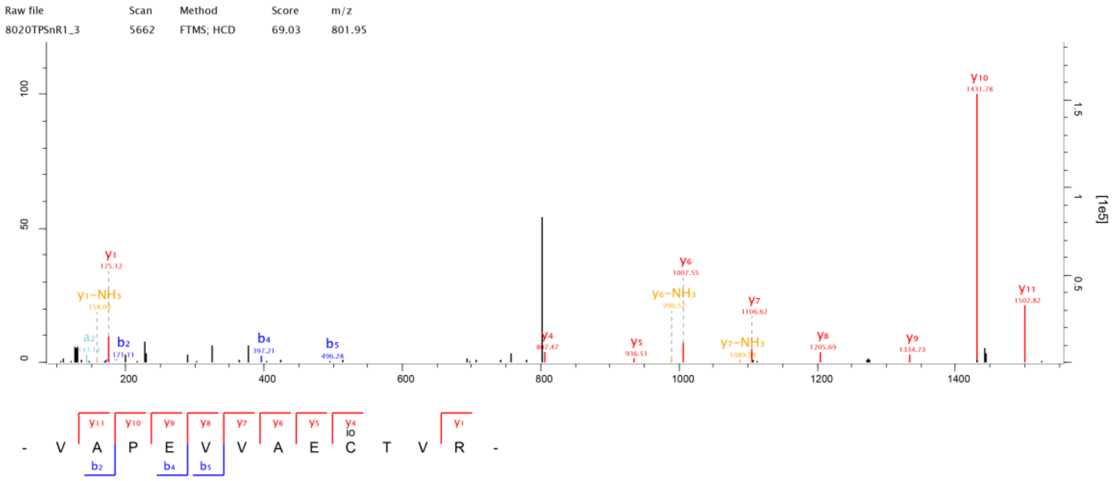

Raw file Scan Method Score m/z
8020TPSnR1_3 5662 FTMS; HCD 69.03 801.95
[1e5]
y10 1431.78
y11 1502.82
y6 1007.55
y6-NH3
y7 1106.62
y7-NH3
y8 1205.69
y9 1334.73
y5 936.51
y4
y1 175.12
y1-NH3
a2
b2
b4 397.21
b5 496.28
- V A P E V V A E C T V R -

b.

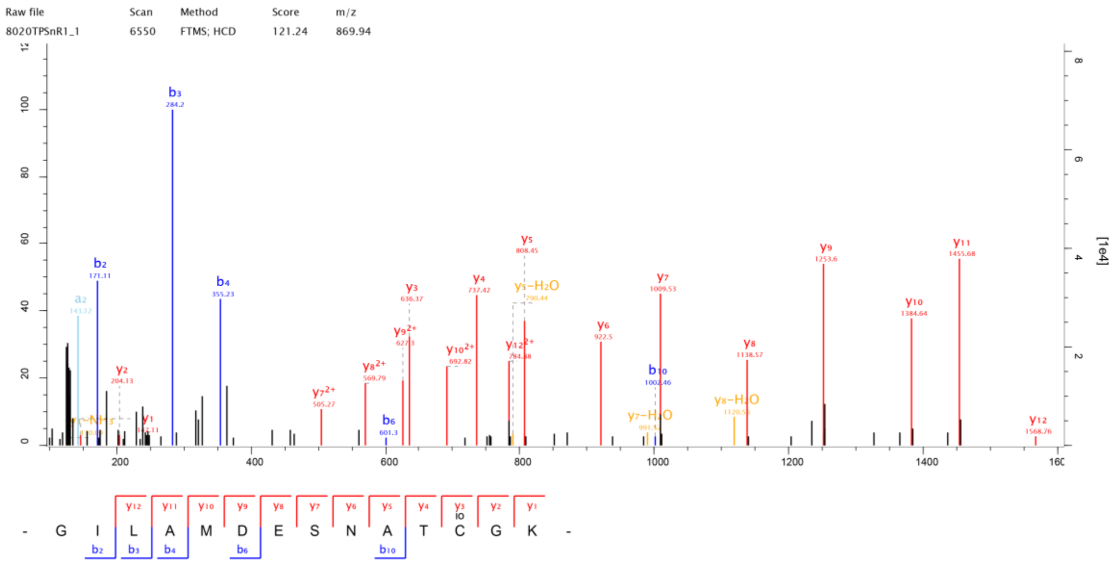

Raw file Scan Method Score m/z
8020TPSnR1_1 6550 FTMS; HCD 121.24 869.94
[1e4]
b3 284.2
b2 171.11
a2
b4 355.23
b6 601.3
b10
y2 204.13
y1
y7 2+ 505.27
y8 2+ 569.79
y9 2+
y3 636.37
y10 2+ 692.82
y4 737.42
y12 2+
y5 808.45
y5-H2O
y6 922.5
y7 1009.53
y7-H2O
y8 1138.57
y8-H2O
y9 1253.6
y10 1384.64
y11 1455.68
y12 1568.76
- G I L A M D E S N A T C G K -

c.

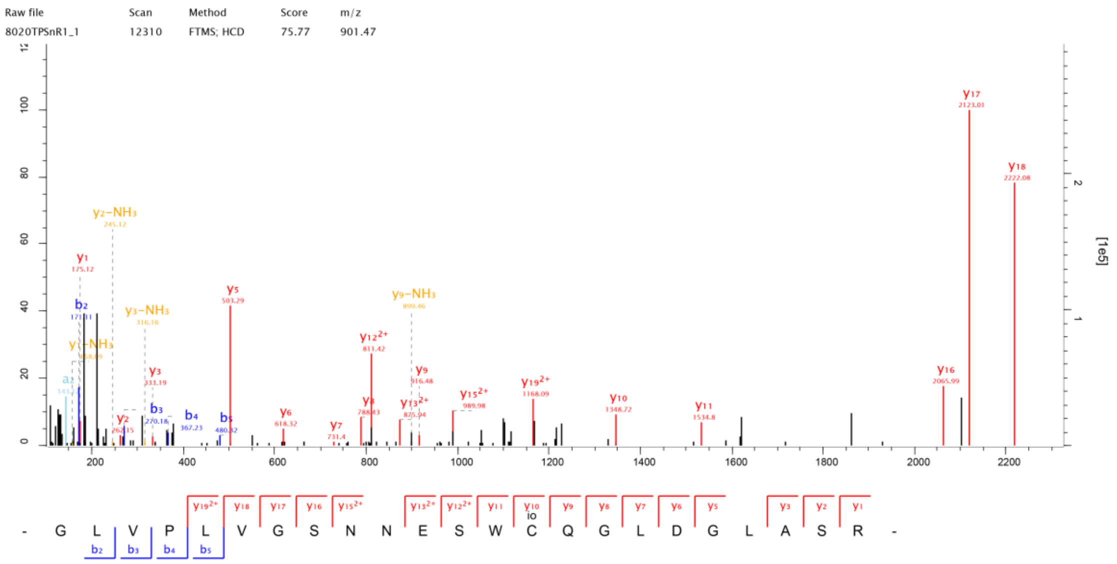

Raw file Scan Method Score m/z
8020TPSnR1_1 12310 FTMS; HCD 75.77 901.47
[1e5]
y17 2123.03
y18 2222.08
y2-NH3 245.12
y1 175.12
b2
y3-NH3 316.16
y1-NH3
y5 503.29
y9-NH3 899.46
y12 2+ 811.42
y3 333.19
y9 916.48
y15 2+ 989.98
y19 2+ 1168.09
y10 1348.72
y11 1534.8
y16 2065.99
y13 2+
y6 618.32
y7 731.4
b3
b4 367.23
b5
y2
a2
- G L V P L V G S N N E S W C Q G L D G L A S R -

d.

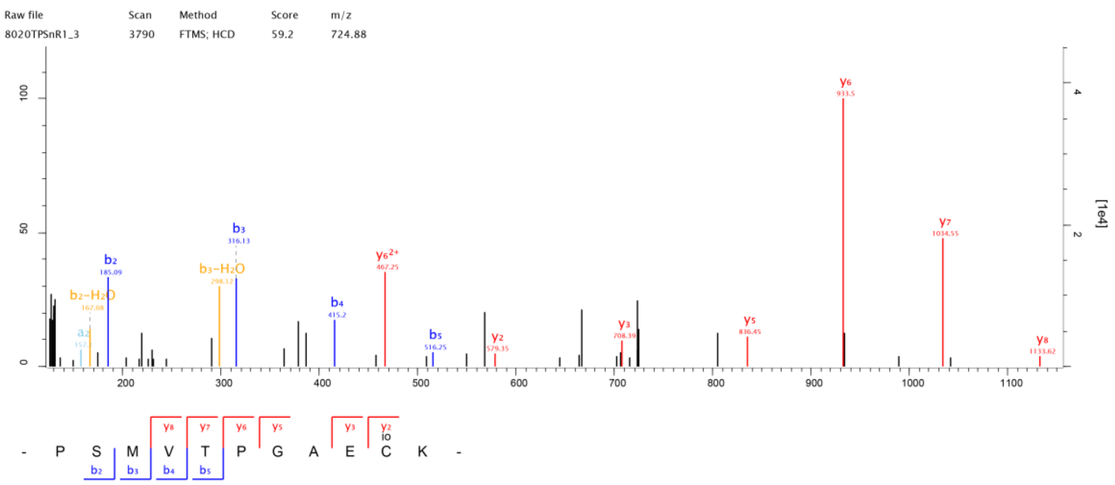

Fig S11: Representative MS/MS spectra of nitrosyl peptides from Calvin cycle related enzyme ALDO. (a) S-nitrosyl-peptide _VAPEVVAEC(io)TVR_ with S-nitrosylation site at C246 related enzyme ALDO (CSA035207.1). (b) S-nitrosyl-peptide _GILAMDESNATC(io)GK_ with S-nitrosylation site at C78 related enzyme ALDO (CSA009966.1). (c) S-nitrosyl-peptide _GLVPLVGSNNESWC(io)QGLDGLASR_ with S-nitrosylation site at C162 related enzyme ALDO (CSA009966.1). (d) S-nitrosyl-peptide _PSMVTPGAEC(io)K_ with S-nitrosylation site at C276 related enzyme ALDO (CSA009966.1).

Table S1. The information for all identified S-nitrosylated proteins.

Protein accession	Position	Amino acid	Protein description	Localization probability	PEP	Score	Modified sequence	Charge	Mass error [ppm]	MS/MS Count
CSA000036.1	632	C	Pyrophosphate-energized vacuolar membrane proton pump OS=Vigna radiata var. radiata PE=1 SV=4	1	0.000464	47.68	DIPGLMEGTAKPDYATC(1)VK	3	0.83527	1
CSA000242.1	125	C	"Malate dehydrogenase 2, cytoplasmic OS=Arabidopsis thaliana GN=MDH2 PE=1 SV=1"	1	0.00021	67.136	SQASALESYAAANC(1)K	3	0.82928	1
CSA000627.1	112	C	"Oxygen-evolving enhancer protein 1, chloroplastic OS=Solanum lycopersicum GN=PSBO PE=2 SV=2"	1	0.000000264	87.429	GTGTANQC(1)PTIEGGVDK	2	-0.080288	2
CSA000627.1	135	C	"Oxygen-evolving enhancer protein 1, chloroplastic OS=Solanum lycopersicum GN=PSBO PE=2 SV=2"	1	0.00242	67.334	FC(1)LEPTSFTVK	2	0.1288	2
CSA000795.1	67	C	"Phosphoribulokinase, chloroplastic OS=Triticum aestivum PE=2 SV=1"	1	0.000000167	97.69	TVVIGLAADSGC(1)GK	2	-1.0173	2
CSA000795.1	106	C	"Phosphoribulokinase, chloroplastic OS=Triticum aestivum PE=2 SV=1"	1	1.82E-12	59.359	GGNPDSNTLLSDMTTVIC(1)LDDYHSLDR	3	-0.2245	1
CSA000795.1	295	C	"Phosphoribulokinase, chloroplastic OS=Triticum aestivum PE=2 SV=1"	1	2.09E-33	128.05	FFSPVYLFDEGSTISWIPC(1)GR	2	-0.12105	3
CSA000918.1	382	C	Glutamate--glyoxylate aminotransferase 2 OS=Arabidopsis thaliana GN=GGAT2 PE=1 SV=1	1	8.89E-15	108.47	NVVC(1)NFTEGAMYSFPQIR	2	-0.2711	2
CSA000918.1	417	C	Glutamate--glyoxylate aminotransferase 2 OS=Arabidopsis thaliana GN=GGAT2 PE=1 SV=1	1	0.00323	63.816	VPDVFYC(1)LK	3	-0.73969	1
CSA000918.1	226	C	Glutamate--glyoxylate aminotransferase 2 OS=Arabidopsis thaliana GN=GGAT2 PE=1 SV=1	1	0.000193	40.968	AMVIINPGNPTGQC(1)LSEANLK	3	-0.53357	2
CSA000918.1	377	C	Glutamate--glyoxylate aminotransferase 2 OS=Arabidopsis thaliana GN=GGAT2 PE=1 SV=1	1	0.0135	63.816	IMTDGFNSC(1)R	2	-0.81489	1
CSA001213.1	13	C	"Putative quinone-oxidoreductase homolog, chloroplastic OS=Arabidopsis thaliana GN=At4g13010 PE=1 SV=1"	1	0.0000055	58.98	LMNAVQYDC(1)YGGGVAGLK	3	-1.657	1
CSA001272.1	248	C	Catalase isozyme 3 OS=Nicotiana plumbaginifolia GN=CAT3 PE=2 SV=1	1	0.0421	60.019	C(1)LLDDEAIK	2	0.96719	1
CSA001272.1	86	C	Catalase isozyme 3 OS=Nicotiana plumbaginifolia GN=CAT3 PE=2 SV=1	1	1.77E-08	70.837	GFFEVTHDISHLTC(1)ADFMR	4	1.0214	1
CSA001302.1	153	C	Non-functional NADPH-dependent codeinone reductase 2 OS=Papaver somniferum GN=COR2 PE=1 SV=1	1	7.33E-09	105.46	SVWEAMEDC(1)QR	2	-0.45387	2
CSA001667.1	87	C	Elongation factor 1-alpha OS=Manihot esculenta GN=EF1 PE=3 SV=1	1	0.00754	42.843	YYC(1)TVIDAPGHR	3	0.75848	1
CSA002108.1	167	C	"Carbonic anhydrase, chloroplastic OS=Spinacia oleracea PE=1 SV=2"	1	0.00000581	74.141	VC(1)PSHVLDFQPGEAFVVR	3	-0.59948	1
CSA002124.1	175	C	"Plastid lipid-associated protein 3, chloroplastic OS=Brassica campestris GN=PAP3 PE=2 SV=1"	1	0.000257	60.157	C(1)LVDSVYGTDFGFR	3	-0.24695	1
CSA002335.1	168	C	Peptidyl-prolyl cis-trans isomerase OS=Catharanthus roseus GN=PCKR1 PE=1 SV=1	1	0.000813	83.862	TVTIADC(1)GQLA	2	0.096927	1
CSA002602.1	40	C	Peptidyl-prolyl cis-trans isomerase OS=Catharanthus roseus GN=PCKR1 PE=1 SV=1	1	0.000000632	100.19	ALC(1)TGEK	2	0.16011	1
CSA002712.1	282	C	Enolase 1 OS=Hevea brasiliensis GN=ENO1 PE=1 SV=1	1	0.00866	74.464	MTC(1)EIGEK	2	-1.0029	2
CSA002712.1	107	C	Enolase 1 OS=Hevea brasiliensis GN=ENO1 PE=1 SV=1	1	1.1E-24	91.733	DPTDQVAIDNFMVQQLDGTVNEWGWC(1)K	4	-0.58525	2
CSA002945.1	9	C	Calcium-binding allergen Bet v 3 OS=Betula pendula GN=BETVIII PE=1 SV=1	1	0.000851	59.067	METTAGTPC(1)K	2	2.3834	1
CSA003060.1	382	C	"Linoleate 13S-lipoxygenase 2-1, chloroplastic OS=Solanum tuberosum GN=LOX2.1 PE=1 SV=1"	1	0.000132	62.162	THC(1)ATEPYIIASNR	4	-1.8096	1
CSA003086.1	516	C	"Ketol-acid reductoisomerase, chloroplastic OS=Spinacia oleracea GN=AHRI PE=1 SV=1"	1	0.000418	57.434	GVSFMVDNC(1)STTAR	3	0.1165	1
CSA003232.1	324	C	Elongation factor 1-gamma OS=Prunus avium PE=2 SV=1	1	4.12E-08	66.893	GFWDMYDPEGYSLWFC(1)DYK	3	0.56802	2
CSA003654.1	30	C	"Ferredoxin-dependent glutamate synthase, chloroplastic OS=Spinacia oleracea GN=FdGOGAT PE=1 SV=3"	1	0.000026	52.185	EPTFC(1)MGDDIPLAILSQK	3	0.52279	1
CSA003658.1	475	C	"Ribulose bisphosphate carboxylase/oxygenase activase 1, chloroplastic OS=Larrea tridentata GN=RCA1 PE=1 SV=1"	1	2.2E-14	75.956	AAQQVHIPVPEGC(1)TDPYATNFDPTAR	3	-0.56842	2
CSA003662.1	175	C	"Ribulose bisphosphate carboxylase/oxygenase activase 2, chloroplastic OS=Larrea tridentata GN=RCA2 PE=2 SV=1"	1	0.0106	66.19	SFQC(1)ELVFAK	2	0.28823	2
CSA003818.1	169	C	"Rhodanese-like domain-containing protein 9, chloroplastic OS=Arabidopsis thaliana GN=STR9 PE=2 SV=1"	1	0.00692	48.284	LLLVC(1)QEGLR	3	-0.20283	1
CSA003990.1	156	C	Uncharacterized protein	1	0.000673	64.82	C(1)VGLQYLEAIR	3	-0.061532	1
CSA004105.1	159	C	ADP-ribosylation factor 1 OS=Oryza sativa subsp. japonica GN=Os01g0813400 PE=2 SV=3	1	7.15E-26	97.057	HWYIQSTC(1)ATSGEGLYEGLDWLSNNIASK	4	0.23309	1
CSA004187.1	239	C	"LL-diaminopimelate aminotransferase, chloroplastic OS=Arabidopsis thaliana GN=DAP PE=1 SV=1"	1	0.000113	47.726	TDVIFFC(1)SPNNPTGSAATR	3	0.47932	1
CSA004197.1	284	C	Probable galactinol--sucrose galactosyltransferase 5 OS=Arabidopsis thaliana GN=RFS5 PE=1 SV=1	1	0.00506	50.353	TSAGEQMPC(1)R	3	-0.5573	1
CSA004395.1	74	C	"Succinate dehydrogenase [ubiquinone] iron-sulfur subunit 2, mitochondrial OS=Arabidopsis thaliana GN=SDH2-2 PE=1 SV=2"	1	0.00332	48.568	EC(1)GPMVLDALIK	3	0.37335	1
CSA004532.1	141	C	"Chlorophyll a-b binding protein CP26, chloroplastic OS=Arabidopsis thaliana GN=LHCB5 PE=1 SV=1"	1	0.00538	58.32	FGANC(1)GPEAVWFK	2	-2.0656	2
CSA004535.1	285	C	Uncharacterized protein	1	0.00226	46.318	GMSC(1)TWDVSETVDK	3	0.74347	1
CSA004763.1	130	C	"Ribulose bisphosphate carboxylase small chain, chloroplastic OS=Betula pendula GN=RBCS1 PE=2 SV=1"	1	0.0011	69.795	LPMFGC(1)TDSSQVLK	2	-1.038	6
CSA004763.1	143	C	"Ribulose bisphosphate carboxylase small chain, chloroplastic OS=Betula pendula GN=RBCS1 PE=2 SV=1"	1	1.85E-13	117.16	EVEEC(1)KK	2	-0.082179	1
CSA004930.1	170	C	Flavanone 3-dioxygenase OS=Petroselinum crispum GN=FHT PE=1 SV=1	1	0.0366	46.016	LMDLAC(1)K	2	-0.27477	1
CSA005033.1	90	C	S-adenosylmethionine synthase 2 OS=Vitis vinifera GN=METK2 PE=3 SV=1	1	0.00000132	79.837	TIGFVSDDVGLDADNC(1)K	3	0.71744	1
CSA005422.1	132	C	DEAD-box ATP-dependent RNA helicase 56 OS=Arabidopsis thaliana GN=RH56 PE=1 SV=2	1	0.00925	40.516	ELAYQIC(1)HEFER	3	2.8488	1
CSA005660.1	449	C	"Leucine aminopeptidase 2, chloroplastic OS=Arabidopsis thaliana GN=LAP2 PE=2 SV=2"	1	0.0000492	52.52	LTLADALVYAC(1)NQGVEK	3	0.52291	1
CSA005866.1	83	C	"PGR5-like protein 1A, chloroplastic OS=Arabidopsis thaliana GN=PGRL1A PE=1 SV=1"	1	0.00374	66.056	ILPYC(1)SIDEK	2	0.53301	2
CSA005966.1	180	C	Basic secretory protease (Fragments) OS=Boswellia serrata PE=1 SV=1	1	4.26E-08	67.645	WDQGYDVTMWFLDYC(1)NGLK	3	-0.089526	1
CSA006518.1	244	C	"Chaperonin CPN60-2, mitochondrial OS=Cucurbita maxima GN=CPN60-2 PE=1 SV=1"	1	0.000438	64.104	C(1)ELDDPLILIYEK	3	0.44573	1
CSA006518.1	135	C	"Chaperonin CPN60-2, mitochondrial OS=Cucurbita maxima GN=CPN60-2 PE=1 SV=1"	1	0.017	44.349	AIFAEGC(1)K	3	0.34202	1
CSA006839.1	412	C	Putative ribonuclease H protein At1g65750 OS=Arabidopsis thaliana GN=At1g65750 PE=3 SV=1	1	3.33E-10	106.16	C(1)GEGWTC(1)TITK	3	-0.16391	1
CSA006839.1	406	C	Putative ribonuclease H protein At1g65750 OS=Arabidopsis thaliana GN=At1g65750 PE=3 SV=1	1	3.33E-10	106.16	C(1)GEGWTC(1)TITK	3	-0.16391	1
CSA006911.1	94	C	60S ribosomal protein L32-1 OS=Arabidopsis thaliana GN=RPL32A PE=2 SV=2	1	0.000278	61.11	TYC(1)AEIAHNVSTQK	3	-0.090893	1
CSA007312.1	246	C	"Alpha-1,4-glucan-protein synthase [UDP-forming] 2 OS=Solanum tuberosum GN=UPTG2 PE=1 SV=1"	1	0.000577	66.267	YDDMWAGWC(1)TK	3	0.41422	2
CSA007724.1	42	C	"Elongation factor Ts, mitochondrial OS=Arabidopsis thaliana GN=EFTS PE=2 SV=1"	1	0.000928	83.045	EETGAGMMDC(1)K	2	0.34427	3
CSA008160.1	78	C	Uncharacterized protein	1	0.0327	69.812	SIFC(1)VLEK	2	0.84301	1
CSA008431.1	172	C	Serine/threonine-protein phosphatase 2A 65 kDa regulatory subunit A beta isoform OS=Arabidopsis thaliana GN=PP2AA2 PE=1 SV=2	1	0.000175	64.04	SIYSQLC(1)QDDMPMVR	3	-1.0094	1
CSA008456.1	150	C	"3-isopropylmalate dehydratase large subunit, chloroplastic OS=Arabidopsis thaliana GN=IIL1 PE=1 SV=1"	1	0.00968	47.96	DFC(1)MEQNIK	3	-0.92432	1
CSA008457.1	124	C	"Thioredoxin-like protein CDSP32, chloroplastic OS=Arabidopsis thaliana GN=CDSP32 PE=1 SV=1"	1	5.85E-10	85.937	TC(1)SDVEFLLVMGDESEETR	3	-0.87574	1
CSA008625.1	588	C	"Glycine--tRNA ligase, mitochondrial 1 OS=Arabidopsis thaliana GN=At1g29880 PE=1 SV=1"	1	0.000386	46.892	C(1)TVFPLVQNQQYEEVAK	3	-1.5098	1
CSA008945.1	280	C	"RuBisCO large subunit-binding protein subunit beta, chloroplastic OS=Brassica napus PE=2 SV=1"	1	2.36E-12	113.71	MAVEFENC(1)K	2	-0.12892	3
CSA009025.1	548	C	NADP-dependent malic enzyme OS=Vitis vinifera PE=2 SV=1	1	0.000257	72.29	YAESC(1)MYTPNYR	2	-0.061848	3
CSA009205.1	309	C	"Succinate dehydrogenase [ubiquinone] flavoprotein subunit 1, mitochondrial OS=Arabidopsis thaliana GN=SDH1-1 PE=1 SV=1"	1	1.27E-14	98.443	TYFSATSAHTC(1)TGDGNAMVAR	3	0.0471	2
CSA009205.1	179	C	"Succinate dehydrogenase [ubiquinone] flavoprotein subunit 1, mitochondrial OS=Arabidopsis thaliana GN=SDH1-1 PE=1 SV=1"	1	5.92E-10	96.673	GSDWLGDQDAIQYMC(1)R	3	-0.31768	1
CSA009282.1	178	C	Mitochondrial-processing peptidase subunit alpha OS=Solanum tuberosum GN=MPP PE=1 SV=1	1	4.43E-08	91.076	TYVPEMVELLIDC(1)VR	3	-0.1753	1
CSA009367.1	231	C	Uncharacterized protein	1	0.0146	47.082	RC(1)LTRASK	2	-3.5597	1
CSA009889.1	82	C	Serine hydroxymethyltransferase 4 OS=Arabidopsis thaliana GN=SHM4 PE=1 SV=1	1	0.0000196	74.141	YYGGNEFIDEIENLC(1)R	3	-1.8331	1
CSA009966.1	276	C	"Fructose-bisphosphate aldolase 2, chloroplastic OS=Arabidopsis thaliana GN=FBA2 PE=1 SV=2"	1	0.000452	59.198	PSMVTPGAEC(1)K	2	0.91888	1
CSA009966.1	162	C	"Fructose-bisphosphate aldolase 2, chloroplastic OS=Arabidopsis thaliana GN=FBA2 PE=1 SV=2"	1	4.22E-13	75.773	GLVPLVGSNNESWC(1)QGLDGLASR	3	-0.1431	2
CSA009966.1	78	C	"Fructose-bisphosphate aldolase 2, chloroplastic OS=Arabidopsis thaliana GN=FBA2 PE=1 SV=2"	1	5.22E-26	121.24	GILAMDESNATC(1)GK	2	-0.30591	5
CSA010160.1	485	C	"Dihydrolipoyl dehydrogenase 1, mitochondrial OS=Arabidopsis thaliana GN=LPD1 PE=1 SV=2"	1	2.8E-17	113.99	TC(1)HAHPTMSEALK	3	0.52199	5
CSA010323.1	385	C	Uncharacterized oxidoreductase At4g09670 OS=Arabidopsis thaliana GN=At4g09670 PE=1 SV=1	1	0.00346	70.056	SIDLGFEPVC(1)L	2	3.7027	1
CSA010519.1	159	C	"Peroxiredoxin Q, chloroplastic OS=Populus jackii GN=PRXQ PE=1 SV=1"	1	0.000000169	67.418	GKPVVVYFYPADETPGC(1)TK	3	1.1742	1
CSA010519.1	113	C	"Peroxiredoxin Q, chloroplastic OS=Populus jackii GN=PRXQ PE=1 SV=1"	1	0.000175	43.164	GKPVVVYFYPADESPGC(1)TK	4	0.50138	1
CSA010527.1	367	C	"Porphobilinogen deaminase, chloroplastic OS=Pisum sativum GN=HEMC PE=1 SV=1"	1	0.000515	85.963	AFLEALDGSC(1)R	2	0.51145	1
CSA010785.1	92	C	Glutamine synthetase cytosolic isozyme 1 OS=Vitis vinifera GN=GS1-1 PE=2 SV=1	1	3.88E-09	65.184	GNNILVMC(1)DTYTPGGEPIPTNK	3	-0.54305	2
CSA010862.1	137	C	"Chlorophyll a-b binding protein CP29.2, chloroplastic OS=Arabidopsis thaliana GN=LHCB4.2 PE=1 SV=1"	1	0.0051	65.252	FREC(1)ELIHGR	4	1.1381	2
CSA011054.1	70	C	"Thioredoxin M-type, chloroplastic OS=Pisum sativum PE=2 SV=1"	1	0.0000247	65.231	LNTDDC(1)PSIATEYGIR	3	-1.0321	2
CSA011328.1	219	C	"Chlorophyll a-b binding protein 7, chloroplastic OS=Solanum lycopersicum GN=CAB7 PE=3 SV=1"	1	0.000000113	66.809	WADILKPGC(1)VNTDPIFPNNK	3	0.30881	1
CSA011328.1	153	C	"Chlorophyll a-b binding protein 7, chloroplastic OS=Solanum lycopersicum GN=CAB7 PE=3 SV=1"	1	0.0000127	96.113	WNVQSELVHC(1)R	2	1.0521	2
CSA011363.1	44	C	Lactoylglutathione lyase OS=Oryza sativa subsp. japonica GN=GLYI-11 PE=1 SV=2	1	0.0176	41.427	FYTEC(1)FGMK	3	-1.0635	1
CSA011544.1	8	C	Glycine-rich RNA-binding protein OS=Daucus carota PE=2 SV=1	1	0.00052	45.684	C(1)FVGGLAWTTDDQSLER	3	-1.2094	1
CSA011668.1	272	C	"Glyceraldehyde-3-phosphate dehydrogenase, cytosolic OS=Antirrhinum majus GN=GAPC PE=2 SV=1"	1	3.15E-10	100.93	VVDLIVHMANTC(1)	2	-0.18415	4
CSA011729.1	269	C	"Allene oxide cyclase, chloroplastic OS=Oryza sativa subsp. japonica GN=AOC PE=1 SV=1"	1	0.0003	84.479	AC(1)EAGSTISNFTD	2	-0.31463	1
CSA011729.1	113	C	"Allene oxide cyclase, chloroplastic OS=Oryza sativa subsp. japonica GN=AOC PE=1 SV=1"	1	0.0000972	77.894	VQELC(1)VYEMNER	3	-0.19638	1
CSA011928.1	471	C	Elongation factor 2 OS=Arabidopsis thaliana GN=LOS1 PE=1 SV=1	1	0.000967	50.324	NC(1)DPEGPLMLYVSK	3	-0.51607	1
CSA012055.1	194	C	"3-ketoacyl-CoA thiolase 2, peroxisomal OS=Arabidopsis thaliana GN=PED1 PE=1 SV=2"	1	4.74E-08	58.107	TMAQAQNC(1)LLPMGVTSENVAHR	3	-0.9516	3
CSA012305.1	125	C	Peroxidase 51 OS=Arabidopsis thaliana GN=PER51 PE=2 SV=1	1	0.00207	51.726	VSC(1)ADILAMATR	3	-0.16746	1
CSA012532.1	446	C	"Transketolase, chloroplastic OS=Solanum tuberosum PE=2 SV=1"	1	0.00785	58.511	NLSQQC(1)LNALAK	2	0.25363	1
CSA012532.1	151	C	"Transketolase, chloroplastic OS=Solanum tuberosum PE=2 SV=1"	1	1.8E-22	87.739	FVLSAGHGC(1)MLQYALLHLAGYDSVQEEDLK	5	0.58936	1
CSA013010.1	224	C	(+)-neomenthol dehydrogenase OS=Capsicum annuum GN=MNR1 PE=1 SV=1	1	0.00474	44.543	LTTEQGAQC(1)PMK	3	-1.5435	1
CSA013547.1	345	C	Heat shock cognate protein 80 OS=Solanum lycopersicum GN=HSC80 PE=2 SV=1	1	0.000175	46.543	VFIMDNC(1)EELMPEYLAFVK	3	0.52199	1
CSA013744.1	393	C	"Serine hydroxymethyltransferase 3, chloroplastic OS=Arabidopsis thaliana GN=SHM3 PE=1 SV=2"	1	0.0091	43.897	AYQNEVVSNC(1)R	3	-2.2449	1
CSA013811.1	345	C	Heat shock cognate protein 80 OS=Solanum lycopersicum GN=HSC80 PE=2 SV=1	1	0.00000236	55.437	VFIMDNC(1)EELIPEYLSFVK	3	0.74183	1
CSA014226.1	153	C	"Zeaxanthin epoxidase, chloroplastic OS=Nicotiana plumbaginifolia GN=ABA2 PE=1 SV=1"	1	0.0205	41.448	TGC(1)ITGDR	3	-0.53495	1
CSA014904.1	26	C	(+)-neomenthol dehydrogenase OS=Capsicum annuum GN=MNR1 PE=1 SV=1	1	0.00952	52.716	GLGFEIC(1)R	3	0.48279	1
CSA015603.1	202	C	"Triosephosphate isomerase, chloroplastic OS=Fragaria ananassa GN=TPI PE=2 SV=1"	1	0.000108	91.265	TFDVC(1)FQQMK	2	-1.3028	1
CSA015823.1	370	C	"NADH dehydrogenase [ubiquinone] flavoprotein 1, mitochondrial OS=Arabidopsis thaliana GN=At5g08530 PE=2 SV=1"	1	0.000356	64.798	HIC(1)DDVLMDYDALK	3	-1.0891	1
CSA015987.1	409	C	"Serine/threonine-protein kinase STN7, chloroplastic OS=Arabidopsis thaliana GN=STN7 PE=1 SV=1"	1	0.0115	46.462	C(1)DYDLVAWR	3	-0.43694	1
CSA015996.1	750	C	"Glycine dehydrogenase (decarboxylating), mitochondrial OS=Solanum tuberosum GN=GDCSP PE=2 SV=1"	1	5.88E-14	69.845	ENLSAFMVTYPSTHGVYEEGIDEIC(1)K	4	-0.90013	1
CSA016043.1	402	C	"ATP-dependent Clp protease ATP-binding subunit ClpA homolog CD4B, chloroplastic OS=Solanum lycopersicum GN=CD4B PE=3 SV=1"	1	0.00245	42.095	GELQC(1)IGATTLDEYR	3	1.0431	1
CSA016104.1	56	C	2-alkenal reductase (NADP(+)-dependent) OS=Nicotiana tabacum GN=DBR PE=1 SV=1	1	0.00766	45.433	NLYLSC(1)DPYMR	3	-0.30401	1
CSA016186.1	329	C	"Formate dehydrogenase, mitochondrial OS=Solanum tuberosum GN=FDH1 PE=1 SV=2"	1	0.00626	40.941	C(1)DIVVINMPLTEK	3	0.53002	1
CSA016186.1	133	C	"Formate dehydrogenase, mitochondrial OS=Solanum tuberosum GN=FDH1 PE=1 SV=2"	1	2.07E-29	125.45	ANEYAEMNPNFVGC(1)AER	3	-0.66705	1
CSA016400.1	101	C	"Ferritin-3, chloroplastic OS=Glycine max PE=2 SV=1"	1	0.000000013	58.373	NNDPQMC(1)EFIESEFLTEQVEAIKK	4	-1.7797	2
CSA016587.1	115	C	"Chlorophyll a-b binding protein 8, chloroplastic OS=Solanum lycopersicum GN=CAB8 PE=3 SV=1"	1	0.0000415	57.148	TAMVGSIGC(1)IAPEILGK	2	-0.06082	5
CSA016736.1	242	C	"Ferredoxin--NADP reductase, leaf isozyme, chloroplastic OS=Pisum sativum GN=PETH PE=1 SV=1"	1	0.0157	57.532	DSTFVYMC(1)GLK	2	-0.22932	1
CSA017430.1	151	C	"Chloroplast stem-loop binding protein of 41 kDa a, chloroplastic OS=Arabidopsis thaliana GN=CSP41A PE=1 SV=1"	1	0.000877	77.662	DC(1)EEWFFDR	3	-0.43789	1
CSA017438.1	242	C	"Alpha-1,4-glucan-protein synthase [UDP-forming] 2 OS=Solanum tuberosum GN=UPTG2 PE=1 SV=1"	1	0.00499	48.284	YDDMWAGWC(1)VK	3	0.37226	1
CSA017504.1	438	C	"Citrate synthase, mitochondrial OS=Daucus carota GN=CS PE=2 SV=1"	1	5.32E-55	156.01	SVTMEWLENYC(1)K	2	-1.7364	3
CSA017525.1	8	C	"Glucose-6-phosphate isomerase, cytosolic 1 OS=Clarkia xantiana GN=PGIC1 PE=3 SV=1"	1	0.00251	54.066	ASSALIC(1)DK	2	-0.20353	1
CSA017604.1	289	C	Uncharacterized protein	1	0.00179	49.483	TPSTDIVC(1)PMYMR	3	-1.8187	1
CSA017616.1	334	C	"Malate dehydrogenase [NADP], chloroplastic OS=Pisum sativum PE=1 SV=2"	1	2.93E-08	50.752	C(1)VAHLTGEGIAVC(1)DLPEDTMLPGEV	4	-0.75966	1
CSA017616.1	346	C	"Malate dehydrogenase [NADP], chloroplastic OS=Pisum sativum PE=1 SV=2"	1	2.93E-08	50.752	C(1)VAHLTGEGIAVC(1)DLPEDTMLPGEV	4	-0.75966	1
CSA017654.1	365	C	"Fructose-1,6-bisphosphatase, chloroplastic OS=Pisum sativum GN=FBP PE=1 SV=2"	1	0.00000574	62.94	LLYEC(1)APMSYLAEQAGGK	3	0.19562	2
CSA018092.1	171	C	"Glutamate-1-semialdehyde 2,1-aminomutase, chloroplastic OS=Nicotiana tabacum GN=GSA PE=2 SV=1"	1	0.00028	59.227	FVNSGTEAC(1)MGVIR	3	-0.88386	1
CSA018159.1	133	C	"3-deoxy-manno-octulosonate cytidylyltransferase, mitochondrial OS=Arabidopsis thaliana GN=KDSB PE=1 SV=1"	1	0.00146	48.527	GFGADVIMTSESC(1)R	3	-0.54544	1
CSA018282.1	4	C	"L-ascorbate peroxidase, cytosolic OS=Pisum sativum GN=APX1 PE=1 SV=2"	1	1.66E-14	118.71	C(1)YPTVSEEYKK	2	-1.2396	3
CSA018288.1	246	C	"2-Cys peroxiredoxin BAS1-like, chloroplastic OS=Arabidopsis thaliana GN=At5g06290 PE=2 SV=3"	1	6.62E-17	89.297	TLQALQYVQENPDEVC(1)PAGWKPGEK	3	-0.47786	4
CSA018664.1	81	C	"Threonine--tRNA ligase, mitochondrial 1 OS=Arabidopsis thaliana GN=THRRS PE=1 SV=3"	1	0.00638	46.796	FQQDDAHIFC(1)R	3	-2.3005	1
CSA018927.1	183	C	Uncharacterized protein	1	0.00745	58.679	AGGLSC(1)K	2	-0.17005	1
CSA018928.1	105	C	Nudix hydrolase 3 OS=Arabidopsis thaliana GN=NUDT3 PE=1 SV=1	1	0.0207	68.224	YC(1)TMTQPLK	2	-0.017358	1
CSA018950.1	367	C	"Glyceraldehyde-3-phosphate dehydrogenase B, chloroplastic OS=Pisum sativum GN=GAPB PE=2 SV=2"	1	0.0000288	71.354	GILAVC(1)DVPLVSVDFR	3	-0.15061	1
CSA018950.1	441	C	"Glyceraldehyde-3-phosphate dehydrogenase B, chloroplastic OS=Pisum sativum GN=GAPB PE=2 SV=2"	1	0.00000648	44.963	WPGMPAQGSGDPLEDFC(1)ETSPAEK	3	-0.88277	1
CSA019572.1	102	C	"Chlorophyll a-b binding protein 37, chloroplastic OS=Petunia sp. GN=CAB37 PE=3 SV=1"	1	7.78E-19	126.28	NRELEVIHC(1)R	4	-2.1784	4
CSA019651.1	496	C	Eukaryotic translation initiation factor 3 subunit D OS=Arabidopsis thaliana GN=TIF3D1 PE=1 SV=1	1	0.00994	51.346	SIVDLC(1)MK	3	-0.93526	1
CSA019750.1	67	C	"Triosephosphate isomerase, cytosolic OS=Petunia hybrida GN=TPIP1 PE=2 SV=1"	1	0.0000315	50.04	DLLRPDFHIAAQNC(1)WVK	4	0.40532	1
CSA019750.1	165	C	"Triosephosphate isomerase, cytosolic OS=Petunia hybrida GN=TPIP1 PE=2 SV=1"	1	0.000439	80.96	ESGC(1)TMDVVAAQTK	2	-2.6197	1
CSA019750.1	153	C	"Triosephosphate isomerase, cytosolic OS=Petunia hybrida GN=TPIP1 PE=2 SV=1"	1	0.000000601	96.745	VIAC(1)IGETLEQR	2	-0.46138	2
CSA019797.1	190	C	Serine/threonine-protein phosphatase 2A 65 kDa regulatory subunit A beta isoform OS=Arabidopsis thaliana GN=PP2AA2 PE=1 SV=2	1	0.00118	45.614	TIYNQLC(1)QDDMPMVR	3	-1.4035	2
CSA019996.1	74	C	"Aminomethyltransferase, mitochondrial OS=Solanum tuberosum GN=GDCST PE=2 SV=1"	1	4.51E-14	116.55	DSIMESTLNC(1)R	2	-0.87002	3
CSA019996.1	220	C	"Aminomethyltransferase, mitochondrial OS=Solanum tuberosum GN=GDCST PE=2 SV=1"	1	4.77E-08	96.464	MLDINGVHC(1)FITR	3	-0.38539	1
CSA019996.1	150	C	"Aminomethyltransferase, mitochondrial OS=Solanum tuberosum GN=GDCST PE=2 SV=1"	1	0.000018	82.515	VTDDHIYLVVNAGC(1)R	3	-0.53688	1
CSA020093.1	42	C	Thaumatin-like protein OS=Actinidia deliciosa GN=tlp PE=1 SV=2	1	0.00802	47.302	TGC(1)TFDGTGR	3	-0.60883	1
CSA020098.1	30	C	40S ribosomal protein SA OS=Daucus carota GN=179B PE=2 SV=1	1	0.0158	45.368	NC(1)DFQMER	3	-0.17544	1
CSA020098.1	148	C	40S ribosomal protein SA OS=Daucus carota GN=179B PE=2 SV=1	1	7.95E-10	82.609	EAALGNIPTIAFC(1)DTDSPMR	3	-0.82755	1
CSA020117.1	745	C	"Glycine dehydrogenase (decarboxylating), mitochondrial OS=Solanum tuberosum GN=GDCSP PE=2 SV=1"	1	6.55E-16	86.791	ANLSALMVTYPSTHGVYEEGIDEIC(1)K	3	-0.5679	1
CSA020587.1	97	C	"Ribulose bisphosphate carboxylase small chain, chloroplastic OS=Brassica napus GN=RBCS PE=2 SV=2"	1	0.00000798	66.826	GWIPC(1)LEFETEHGFVYR	4	-0.42028	1
CSA020605.1	293	C	Ribulose bisphosphate carboxylase large chain OS=Cucumis sativus GN=rbcL PE=1 SV=5	1	0.00394	59.67	WSPELAAAC(1)EVWK	2	-0.49279	2
CSA020775.1	102	C	Uncharacterized oxidoreductase At4g09670 OS=Arabidopsis thaliana GN=At4g09670 PE=1 SV=1	1	0.0000018	50.226	MKPDLDALGALGDAGWYC(1)VR	4	-0.49731	1
CSA021163.1	131	C	Stem-specific protein TSJT1 OS=Nicotiana tabacum GN=TSJT1 PE=2 SV=1	1	0.00224	42.947	SFAPFPTGC(1)MYQNER	3	-0.69067	1
CSA021473.1	12	C	Uncharacterized protein	1	0.0000653	75.739	SGAQGAQPPEC(1)K	2	0.71888	1
CSA021512.1	317	C	Probable 2-oxoglutarate-dependent dioxygenase At3g49630 OS=Arabidopsis thaliana GN=At3g50210 PE=2 SV=1	1	0.0022	75.213	C(1)ETYMLQR	3	0.59249	1
CSA021957.1	50	C	"Epimerase family protein SDR39U1 homolog, chloroplastic OS=Arabidopsis thaliana GN=GC1 PE=2 SV=2"	1	1.75E-09	52.09	DC(1)IQGSNAVVNLAGMPISTR	3	0.32526	1
CSA022347.1	148	C	Uncharacterized protein	1	0.00019	58.079	ESPVWMDSSTTEQC(1)K	3	-0.9036	1
CSA022360.1	254	C	Methyl-CpG-binding domain-containing protein 11 OS=Arabidopsis thaliana GN=MBD11 PE=1 SV=1	1	0.000867	89.08	C(1)QMEGQEK	2	-0.10202	1
CSA022661.1	155	C	"Plastocyanin, chloroplastic OS=Pisum sativum GN=PETE PE=3 SV=1"	1	0.000055	51.495	GSYGFYC(1)TPHQGAGMVGK	2	-0.65473	1
CSA022831.1	462	C	Uncharacterized protein	1	0.00745	45.653	C(1)TYDGNLPTTK	3	-0.85112	1
CSA023566.1	181	C	Probable methionine--tRNA ligase OS=Oryza sativa subsp. japonica GN=Os06g0508700 PE=2 SV=2	1	0.0000748	52.527	TSSPQQTEVC(1)QAIFMK	3	-0.42139	1
CSA023611.1	57	C	Sm-like protein LSM3A OS=Arabidopsis thaliana GN=LSM3A PE=1 SV=1	1	0.00152	62.466	HC(1)NMVLENVR	3	-0.080421	1
CSA024064.1	92	C	"Chlorophyll a-b binding protein 6A, chloroplastic OS=Solanum lycopersicum GN=CAB6A PE=2 SV=1"	1	0.00561	71.501	YKESELIHC(1)R	4	-1.279	2
CSA024718.1	84	C	Chalcone synthase 1 OS=Camellia sinensis GN=CHS1 PE=2 SV=1	1	0.0000683	53.255	ENPQVC(1)EYMAPSLDAR	3	-0.024236	1
CSA024855.1	214	C	Alpha-galactosidase 3 OS=Arabidopsis thaliana GN=AGAL3 PE=1 SV=1	1	0.000506	47.301	HDC(1)VFGTDLNLDEIR	4	0.9282	1
CSA024857.1	496	C	Inositol-3-phosphate synthase OS=Nicotiana paniculata GN=INPS1 PE=2 SV=1	1	0.00118	42.257	AC(1)VGLAPENNMILEYK	3	0.21184	1
CSA024921.1	377	C	Glutamate--glyoxylate aminotransferase 2 OS=Arabidopsis thaliana GN=GGAT2 PE=1 SV=1	1	0.024	49.934	AQIMTDGFNSC(1)R	2	0.24111	1
CSA025026.1	112	C	Beta-D-xylosidase 4 OS=Arabidopsis thaliana GN=BXL4 PE=1 SV=1	1	0.000224	84.479	TMIGNYEGTPC(1)K	2	0.18052	6
CSA025110.1	322	C	"Ferredoxin--NADP reductase, leaf isozyme, chloroplastic OS=Pisum sativum GN=PETH PE=1 SV=1"	1	0.0000314	91.549	KDNTYVYMC(1)GLK	3	-1.8003	3
CSA025612.1	591	C	Probable cytosolic oligopeptidase A OS=Arabidopsis thaliana GN=CYOP PE=1 SV=1	1	0.00028	59.227	HYETGEC(1)LPEDIYR	3	-0.64472	1
CSA025613.1	367	C	"Serine hydroxymethyltransferase, mitochondrial OS=Solanum tuberosum PE=2 SV=1"	1	0.002	52.071	AYQEQVLSNC(1)SK	3	0.9742	1
CSA025613.1	129	C	"Serine hydroxymethyltransferase, mitochondrial OS=Solanum tuberosum PE=2 SV=1"	1	4.51E-38	131.4	YYGGNEYIDMAESLC(1)QK	3	0.21626	4
CSA025704.1	205	C	"Aconitate hydratase 2, mitochondrial OS=Arabidopsis thaliana GN=ACO2 PE=1 SV=2"	1	0.000113	47.726	VLLQDFTGVPAVVDLAC(1)MR	3	1.1444	1
CSA026073.1	173	C	"Stromal 70 kDa heat shock-related protein, chloroplastic OS=Pisum sativum GN=HSP70 PE=2 SV=1"	1	0.0205	41.429	LQC(1)PAMGK	3	-2.5057	1
CSA026411.1	77	C	"Probable lactoylglutathione lyase, chloroplastic OS=Arabidopsis thaliana GN=At1g67280 PE=1 SV=1"	1	0.000000103	105.1	FYTEC(1)LGMK	2	-1.5568	2
CSA026696.1	260	C	5-methyltetrahydropteroyltriglutamate--homocysteine methyltransferase OS=Catharanthus roseus GN=METE PE=2 SV=1	1	0.00477	48.513	C(1)VTGFGFDLVR	3	-0.38625	1
CSA026796.1	346	C	"Glyceraldehyde-3-phosphate dehydrogenase A, chloroplastic OS=Spinacia oleracea GN=GAPA PE=1 SV=2"	1	0.00000147	82.59	GILSVC(1)DEPLVSVDFR	3	-0.040816	1
CSA026821.1	341	C	Thiol protease aleurain-like OS=Arabidopsis thaliana GN=At3g45310 PE=2 SV=1	1	0.000342	87.184	DGVYTSDTC(1)GK	2	0.38223	1
CSA027795.1	198	C	Monodehydroascorbate reductase OS=Solanum lycopersicum GN=AFRR PE=1 SV=1	1	0.000781	40.493	LNNLDVNMVYPEPWC(1)MPR	3	-0.19876	1
CSA028285.1	185	C	"Ornithine aminotransferase, mitochondrial OS=Arabidopsis thaliana GN=DELTA-OAT PE=1 SV=1"	1	0.000305	55.335	TLAVISMSC(1)DNEATR	3	-0.60195	1
CSA028496.1	362	C	Phospholipase D alpha 1 OS=Ricinus communis GN=PLD1 PE=1 SV=1	1	0.00345	43.03	IMSFVGGIDLC(1)DGR	3	-0.19408	1
CSA028657.1	238	C	Actin-depolymerizing factor 2 OS=Petunia hybrida GN=ADF2 PE=2 SV=1	1	0.0000998	74.789	ANAASGMAVHDDC(1)K	3	-0.77268	3
CSA028657.1	304	C	Actin-depolymerizing factor 2 OS=Petunia hybrida GN=ADF2 PE=2 SV=1	1	0.000698	45.614	YAVYDYDFVTEENC(1)QK	3	-1.0891	1
CSA028748.1	156	C	"Elongation factor Tu, chloroplastic OS=Glycine max GN=TUFB1 PE=3 SV=1"	1	5.04E-39	138.73	HYAHVDC(1)PGHADYVK	3	0.69106	3
CSA028748.1	458	C	"Elongation factor Tu, chloroplastic OS=Glycine max GN=TUFB1 PE=3 SV=1"	1	1.6E-09	92.38	MIVELIMPVAC(1)EQGMR	3	0.53542	4
CSA028976.1	478	C	Uncharacterized mitochondrial protein AtMg01250 OS=Arabidopsis thaliana GN=AtMg01250 PE=4 SV=1	1	0.0138	51.193	TVVC(1)SMYGK	2	-2.0608	1
CSA029266.1	228	C	Eukaryotic translation initiation factor 3 subunit I OS=Arabidopsis thaliana GN=TIF3I1 PE=2 SV=2	1	0.000419	47.548	SYNMPC(1)NIESASLEPK	3	-2.2365	1
CSA029580.1	258	C	26S protease regulatory subunit 7 OS=Prunus persica GN=RPT1 PE=2 SV=1	1	0.00122	56.205	SVC(1)TEAGMYAIR	2	-1.9388	2
CSA029723.1	158	C	"Glycine cleavage system H protein, mitochondrial OS=Flaveria anomala GN=GDCSH PE=2 SV=1"	1	0.0338	63.216	FC(1)QEEDAAH	2	0.0080499	1
CSA029743.1	184	C	UTP--glucose-1-phosphate uridylyltransferase OS=Musa acuminata GN=UGPA PE=2 SV=1	1	0.00104	57.175	LIVEDFMPLPC(1)K	3	-0.58377	1
CSA029934.1	193	C	Probable aldo-keto reductase 2 OS=Oryza sativa subsp. indica GN=OsI_15387 PE=3 SV=1	1	0.00141	79.659	DIEADIVPTC(1)R	2	0.29583	2
CSA030116.1	65	C	Aldehyde dehydrogenase family 7 member B4 OS=Arabidopsis thaliana GN=ALDH7B4 PE=2 SV=3	1	0.000088	51.03	AC(1)SEAANMWMQIPAPK	3	-0.76523	1
CSA030124.1	45	C	SKP1-like protein 11 OS=Arabidopsis thaliana GN=ASK11 PE=1 SV=1	1	0.00021	67.136	SLLDLTC(1)QTVADMIK	3	0.40754	1
CSA030275.1	209	C	AP-1 complex subunit mu-2 OS=Arabidopsis thaliana GN=AP1M2 PE=1 SV=1	1	0.0219	58.699	TYLSGMPEC(1)K	2	0.43444	1
CSA030434.1	107	C	40S ribosomal protein S12 OS=Hordeum vulgare GN=RPS12 PE=2 SV=1	1	0.000441	88.495	TLGEWAGLC(1)K	2	0.88899	2
CSA030616.1	18	C	Glutathione S-transferase OS=Hyoscyamus muticus PE=1 SV=1	1	0.00298	87.476	VFAC(1)LNEK	2	0.96074	1
CSA030616.1	85	C	Glutathione S-transferase OS=Hyoscyamus muticus PE=1 SV=1	1	0.00922	67.334	GNQLIC(1)QDPK	2	-0.77756	1
CSA030920.1	139	C	26S proteasome non-ATPase regulatory subunit 1 homolog A OS=Arabidopsis thaliana GN=RPN2A PE=1 SV=1	1	0.000329	92.112	YQQAIGMAIEC(1)R	2	0.371	2
CSA031127.1	277	C	UDP-glucuronic acid decarboxylase 6 OS=Arabidopsis thaliana GN=UXS6 PE=2 SV=1	1	0.000425	68.044	SFC(1)YVSDMVDGLIR	3	0.59642	1
CSA031259.1	50	C	CSC1-like protein ERD4 OS=Arabidopsis thaliana GN=ERD4 PE=2 SV=1	1	0.005	57.55	TGC(1)LGLTGK	3	0.142	1
CSA031477.1	229	C	Sorbitol dehydrogenase OS=Arabidopsis thaliana GN=SDH PE=1 SV=1	1	1.81E-25	122.39	TMSTALSATC(1)PGGK	2	0.16057	2
CSA031477.1	19	C	Sorbitol dehydrogenase OS=Arabidopsis thaliana GN=SDH PE=1 SV=1	1	0.00823	44.692	C(1)AGFEVK	2	-0.62666	1
CSA031781.1	42	C	Adenosylhomocysteinase OS=Petroselinum crispum GN=SAHH PE=2 SV=2	1	0.000000131	88.385	LELELAEVEMPGLMSC(1)R	3	0.012224	2
CSA031781.1	120	C	Adenosylhomocysteinase OS=Petroselinum crispum GN=SAHH PE=2 SV=2	1	0.000783	56.569	GETLQEYWWC(1)TER	3	-2.1593	1
CSA032333.1	200	C	"Pyruvate dehydrogenase E1 component subunit alpha-3, chloroplastic OS=Oryza sativa subsp. japonica GN=Os04g0119400 PE=2 SV=2"	1	0.00000252	49.768	LEQAYC(1)GTIGYEYMHIADR	4	-1.2106	1
CSA032553.1	445	C	UDP-sugar pyrophosphorylase OS=Cucumis melo GN=USP PE=1 SV=1	1	0.00108	66.056	LEC(1)MMQDYPK	3	-1.1725	1
CSA032664.1	159	C	ADP-ribosylation factor 2 OS=Oryza sativa subsp. japonica GN=ARF PE=2 SV=2	1	2.92E-31	108.44	HWYIQSTC(1)ATSGEGLYEGLDWLSNNIANK	4	0.36378	1
CSA032885.1	17	C	Phosphoenolpyruvate carboxylase 2 OS=Sorghum bicolor PE=3 SV=1	1	0.0215	43.124	DVC(1)LLAR	3	-0.28067	1
CSA033259.1	141	C	"Oxygen-evolving enhancer protein 2-1, chloroplastic OS=Nicotiana tabacum GN=PSBP1 PE=3 SV=2"	1	8.66E-10	82.01	YEDNFDPNSNVC(1)VMVTPTDK	3	-0.36718	2
CSA033261.1	559	C	"2,3-bisphosphoglycerate-independent phosphoglycerate mutase OS=Mesembryanthemum crystallinum GN=PGM1 PE=2 SV=1"	1	3.54E-12	62.589	VNLPNSDMVGHTGDIDATVVAC(1)K	4	-0.18778	1
CSA033307.1	179	C	Auxin-induced protein PCNT115 OS=Nicotiana tabacum PE=2 SV=1	1	0.000137	72.891	RLDMDC(1)IDLYYQHR	4	0.15624	2
CSA033864.1	158	C	"Photosystem I reaction center subunit N, chloroplastic OS=Arabidopsis thaliana GN=PSAN PE=1 SV=2"	1	1.33E-08	102.52	FLSEDLELEC(1)K	2	0.30469	2
CSA033864.1	138	C	"Photosystem I reaction center subunit N, chloroplastic OS=Arabidopsis thaliana GN=PSAN PE=1 SV=2"	1	0.00143	51.463	FPENFTGC(1)QDLAK	3	-1.3574	1
CSA033994.1	291	C	"ATP synthase subunit beta, mitochondrial OS=Nicotiana plumbaginifolia GN=ATPB PE=1 SV=1"	1	9.2E-13	105.03	C(1)ALVYGQMNEPPGAR	3	-0.7066	3
CSA034340.1	50	C	NADP-dependent glyceraldehyde-3-phosphate dehydrogenase OS=Nicotiana plumbaginifolia GN=GAPN PE=2 SV=1	1	0.000513	67.214	VQAC(1)TQEEVNK	3	-0.13997	1
CSA034373.1	33	C	ATP-citrate synthase beta chain protein 1 OS=Oryza sativa subsp. japonica GN=ACLB-1 PE=2 SV=1	1	9.04E-10	107.79	MLDFDFLC(1)GR	3	0.28414	1
CSA034381.1	107	C	Enolase 2 OS=Zea mays GN=ENO2 PE=2 SV=1	1	3.86E-13	93.5	IDNFMVQELDGTVNEWGWC(1)K	3	0.038153	1
CSA034422.1	590	C	Sucrose synthase 2 OS=Pisum sativum GN=SUS2 PE=2 SV=1	1	0.00903	61.344	NITGLVEC(1)YAK	2	-2.7604	1
CSA034492.1	97	C	"Sedoheptulose-1,7-bisphosphatase, chloroplastic OS=Spinacia oleracea PE=2 SV=1"	1	0.0101	45.433	LMVC(1)MGEALR	3	-1.0913	1
CSA034513.1	117	C	Proteasome subunit alpha type-5 OS=Glycine max GN=PAE1 PE=2 SV=1	1	5.02E-10	78.708	FSYGEPMTVESTTQALC(1)DLALR	3	-0.62439	1
CSA034775.1	516	C	"Malate dehydrogenase 1, mitochondrial OS=Arabidopsis thaliana GN=At1g53240 PE=1 SV=1"	1	0.00000762	77.776	ALEGC(1)DIVIIPAGVPR	3	-0.18994	1
CSA034838.1	100	C	"6-phosphogluconate dehydrogenase, decarboxylating 3 OS=Arabidopsis thaliana GN=At3g02360 PE=2 SV=1"	1	0.000664	44.391	GDC(1)IIDGGNEWYENTER	3	-0.26251	1
CSA034927.1	195	C	"GDP-mannose 3,5-epimerase 2 OS=Oryza sativa subsp. japonica GN=GME-2 PE=2 SV=2"	1	0.00997	57.177	DFGIEC(1)R	3	-1.1661	1
CSA035044.1	81	C	"Glutathione S-transferase DHAR3, chloroplastic OS=Arabidopsis thaliana GN=DHAR3 PE=1 SV=1"	1	0.0149	43.635	LGDC(1)PFTQR	3	-0.75353	1
CSA035053.1	40	C	Uncharacterized protein	1	0.00055	55.724	TC(1)GSPGGPPITAPR	3	0.40537	1
CSA035117.1	90	C	"Glutaredoxin-C5, chloroplastic OS=Arabidopsis thaliana GN=GRXC5 PE=1 SV=1"	1	0.00104	86.898	SWC(1)SYSSEVK	2	0.84236	3
CSA035207.1	246	C	"Fructose-bisphosphate aldolase, cytoplasmic isozyme OS=Cicer arietinum GN=ALDC PE=2 SV=1"	1	0.00346	69.03	VAPEVVAEC(1)TVR	2	-0.23922	1
CSA035327.1	149	C	"3-ketoacyl-CoA thiolase 2, peroxisomal OS=Arabidopsis thaliana GN=PED1 PE=1 SV=2"	1	3.04E-10	79.143	TMAQAQDC(1)LLPMGVTSENVAHR	3	0.12557	3
CSA035382.1	9	C	Glycine-rich RNA-binding protein OS=Daucus carota PE=2 SV=1	1	0.000000684	84.41	C(1)FVGGLAWATDGQSLER	3	0.76304	1
CSA035408.1	468	C	Catalase isozyme 1 OS=Gossypium hirsutum GN=CAT1 PE=2 SV=2	1	0.00000348	66.068	VTHEIC(1)SIWISYWSQADR	4	-1.823	1
CSA035408.1	90	C	Catalase isozyme 1 OS=Gossypium hirsutum GN=CAT1 PE=2 SV=2	1	2.96E-56	145.91	GFFEVTHDISQLTC(1)ADFLR	4	-0.59482	2
CSA035465.1	28	C	T-complex protein 1 subunit alpha OS=Arabidopsis thaliana GN=CCT1 PE=1 SV=1	1	0.00167	45.207	TQNVMAC(1)QAVANIVK	3	1.0337	1
CSA035914.1	327	C	UDP-glucose 4-epimerase GEPI48 OS=Cyamopsis tetragonoloba PE=2 SV=1	1	0.0185	43.083	YGVEEMC(1)R	3	-1.4114	1
CSA036240.1	11	C	40S ribosomal protein S16 OS=Gossypium hirsutum GN=RPS16 PE=2 SV=1	1	0.000573	51.726	AAPPVESVQC(1)FGR	2	0.29173	1
CSA036240.1	24	C	40S ribosomal protein S16 OS=Gossypium hirsutum GN=RPS16 PE=2 SV=1	1	0.0357	62.463	TAVAVTYC(1)K	2	-1.382	1
CSA036476.1	9	C	Tropinone reductase homolog OS=Datura stramonium PE=2 SV=1	1	0.00476	46.39	ASEELMSC(1)R	2	-0.31749	1
CSA036590.1	218	C	"RuBisCO large subunit-binding protein subunit beta, chloroplastic OS=Pisum sativum PE=1 SV=2"	1	1.03E-26	130.75	MTVEYENC(1)K	2	-0.6429	2
CSA036672.1	463	C	"Crocetin glucosyltransferase, chloroplastic OS=Gardenia jasminoides GN=UGT75L6 PE=1 SV=1"	1	0.00133	52.247	NLMAFVEEVGGC(1)F	3	-0.15627	1
CSA036932.1	63	C	"ATP synthase subunit beta, chloroplastic OS=Agapanthus africanus GN=atpB PE=3 SV=1"	1	7.64E-11	88.176	DTVGQPINVTC(1)EVQQLLGNNR	3	1.4684	1
CSA036936.1	42	C	Ribulose bisphosphate carboxylase large chain (Fragment) OS=Cyrilla racemiflora GN=rbcL PE=3 SV=1	1	0.0136	42.336	VALEAC(1)VQAR	3	0.73245	3
CSA036938.1	207	C	Ribulose bisphosphate carboxylase large chain OS=Carpinus caroliniana GN=rbcL PE=3 SV=1	1	0.0169	47.849	AVYEC(1)LR	3	-0.9748	1
CSA036938.1	262	C	Ribulose bisphosphate carboxylase large chain OS=Carpinus caroliniana GN=rbcL PE=3 SV=1	1	6.22E-77	158.65	GHYLNATAGTC(1)EEMMKR	4	-0.061731	23
CSA036938.1	299	C	Ribulose bisphosphate carboxylase large chain OS=Carpinus caroliniana GN=rbcL PE=3 SV=1	1	1.24E-15	59.912	ELGVPIVMHDYLTGGFTANTSLAHYC(1)R	5	-0.51643	5

Table S2. The information of secondary structures.

Type	Non-modified Cys	Modified Cys	P value
Surface accessibility	36.65	33.48	0.2799
Alpha-helix	0.1048	0.1472	0.43936
Beta-strand	0.0537	0.0581	0.47572
Coil	0.8413	0.7947	8.92E-02

Table S3. The information of functional classification and subcellular localization.

GO Terms Level 1	GO Terms Level 2	No. of Protein	Protein IDs
**Biological Process**	metabolic process	114	CSA003232.1 CSA026821.1 CSA023611.1 CSA027795.1 CSA020605.1 CSA017525.1 CSA009282.1 CSA023566.1 CSA017616.1 CSA034340.1 CSA032553.1 CSA025704.1 CSA034492.1 CSA015823.1 CSA012532.1 CSA032333.1 CSA034422.1 CSA035914.1 CSA024855.1 CSA016736.1 CSA018092.1 CSA008456.1 CSA028748.1 CSA010519.1 CSA001667.1 CSA015603.1 CSA034775.1 CSA033994.1 CSA000242.1 CSA033864.1 CSA024921.1 CSA017654.1 CSA000795.1 CSA009889.1 CSA025110.1 CSA018950.1 CSA029580.1 CSA035327.1 CSA025026.1 CSA030434.1 CSA016043.1 CSA015996.1 CSA003990.1 CSA009205.1 CSA003086.1 CSA007312.1 CSA028496.1 CSA034513.1 CSA017438.1 CSA002712.1 CSA010527.1 CSA035207.1 CSA009025.1 CSA009966.1 CSA012305.1 CSA025613.1 CSA025612.1 CSA006911.1 CSA014226.1 CSA011668.1 CSA034838.1 CSA030124.1 CSA019750.1 CSA026796.1 CSA002602.1 CSA004930.1 CSA008625.1 CSA019996.1 CSA029743.1 CSA004187.1 CSA001302.1 CSA020117.1 CSA036932.1 CSA016186.1 CSA021512.1 CSA036672.1 CSA022347.1 CSA017604.1 CSA034373.1 CSA033261.1 CSA036938.1 CSA002335.1 CSA003654.1 CSA018664.1 CSA031477.1 CSA010160.1 CSA035408.1 CSA001272.1 CSA024857.1 CSA004395.1 CSA018282.1 CSA020098.1 CSA007724.1 CSA013744.1 CSA029723.1 CSA000918.1 CSA010785.1 CSA012055.1 CSA031781.1 CSA030116.1 CSA026696.1 CSA000627.1 CSA036240.1 CSA032885.1 CSA003060.1 CSA024718.1 CSA005660.1 CSA005033.1 CSA017504.1 CSA034381.1 CSA018288.1 CSA011054.1 CSA015987.1 CSA033259.1
	cellular process	81	CSA003232.1 CSA023611.1 CSA008945.1 CSA017525.1 CSA036590.1 CSA023566.1 CSA017616.1 CSA032333.1 CSA034422.1 CSA018092.1 CSA028748.1 CSA035465.1 CSA010519.1 CSA001667.1 CSA015603.1 CSA034775.1 CSA033994.1 CSA000242.1 CSA033864.1 CSA009889.1 CSA026073.1 CSA008457.1 CSA006518.1 CSA030434.1 CSA015996.1 CSA003990.1 CSA009205.1 CSA003086.1 CSA007312.1 CSA028496.1 CSA034513.1 CSA017438.1 CSA002712.1 CSA010527.1 CSA035207.1 CSA028657.1 CSA009025.1 CSA009966.1 CSA012305.1 CSA006911.1 CSA014226.1 CSA025613.1 CSA032664.1 CSA034838.1 CSA019750.1 CSA030124.1 CSA002602.1 CSA008625.1 CSA019996.1 CSA004187.1 CSA029743.1 CSA020117.1 CSA036932.1 CSA002335.1 CSA003654.1 CSA016400.1 CSA018664.1 CSA010160.1 CSA024857.1 CSA035117.1 CSA004395.1 CSA004105.1 CSA020098.1 CSA007724.1 CSA013744.1 CSA010785.1 CSA029723.1 CSA000627.1 CSA026696.1 CSA031781.1 CSA036240.1 CSA032885.1 CSA005033.1 CSA017504.1 CSA013547.1 CSA034381.1 CSA018288.1 CSA011054.1 CSA015987.1 CSA033259.1 CSA013811.1
	single-organism process	77	CSA027795.1 CSA020605.1 CSA017525.1 CSA023566.1 CSA017616.1 CSA034340.1 CSA000036.1 CSA015823.1 CSA032333.1 CSA034422.1 CSA035914.1 CSA016736.1 CSA002108.1 CSA010519.1 CSA015603.1 CSA034775.1 CSA033994.1 CSA000242.1 CSA009889.1 CSA025110.1 CSA018950.1 CSA008457.1 CSA015996.1 CSA003990.1 CSA009205.1 CSA007312.1 CSA003086.1 CSA028496.1 CSA017438.1 CSA002712.1 CSA035207.1 CSA028657.1 CSA009025.1 CSA009966.1 CSA012305.1 CSA014226.1 CSA025613.1 CSA032664.1 CSA011668.1 CSA034838.1 CSA019750.1 CSA004930.1 CSA026796.1 CSA008625.1 CSA019996.1 CSA004187.1 CSA001302.1 CSA020117.1 CSA036932.1 CSA016186.1 CSA021512.1 CSA033261.1 CSA036938.1 CSA003654.1 CSA016400.1 CSA018664.1 CSA010160.1 CSA031477.1 CSA035408.1 CSA001272.1 CSA024857.1 CSA035117.1 CSA004395.1 CSA004105.1 CSA018282.1 CSA013744.1 CSA010785.1 CSA029723.1 CSA026696.1 CSA030116.1 CSA031781.1 CSA003060.1 CSA032885.1 CSA017504.1 CSA034381.1 CSA018288.1 CSA011054.1
	biological regulation	11	CSA008457.1 CSA010519.1 CSA000627.1 CSA032664.1 CSA035117.1 CSA004105.1 CSA018288.1 CSA011054.1 CSA016400.1 CSA030920.1 CSA010160.1
	response to stimulus	8	CSA035408.1 CSA013547.1 CSA001272.1 CSA018282.1 CSA012305.1 CSA004105.1 CSA032664.1 CSA013811.1
	localization	4	CSA033994.1 CSA036932.1 CSA000036.1 CSA030275.1
	other	3	CSA028657.1 CSA004105.1 CSA032664.1
**Cellular Component**	cell	36	CSA028657.1 CSA023611.1 CSA014226.1 CSA006911.1 CSA032664.1 CSA008945.1 CSA018159.1 CSA008625.1 CSA036590.1 CSA023566.1 CSA011729.1 CSA033261.1 CSA018664.1 CSA030275.1 CSA028748.1 CSA001667.1 CSA022360.1 CSA033994.1 CSA033864.1 CSA004105.1 CSA020098.1 CSA007724.1 CSA029580.1 CSA030920.1 CSA019651.1 CSA029723.1 CSA000627.1 CSA036240.1 CSA005660.1 CSA006518.1 CSA030434.1 CSA003990.1 CSA034381.1 CSA034513.1 CSA002712.1 CSA033259.1
	macromolecular complex	16	CSA019651.1 CSA029723.1 CSA023611.1 CSA006911.1 CSA000627.1 CSA036240.1 CSA033994.1 CSA030434.1 CSA034381.1 CSA033864.1 CSA034513.1 CSA002712.1 CSA020098.1 CSA033259.1 CSA030275.1 CSA030920.1
	organelle	10	CSA011729.1 CSA028657.1 CSA023611.1 CSA014226.1 CSA006911.1 CSA036240.1 CSA022360.1 CSA033994.1 CSA030434.1 CSA020098.1
	membrane	9	CSA000036.1 CSA014226.1 CSA000627.1 CSA033994.1 CSA033864.1 CSA031259.1 CSA028496.1 CSA030275.1 CSA033259.1
**Molecular Function**	catalytic activity	116	CSA026821.1 CSA004197.1 CSA027795.1 CSA011928.1 CSA009367.1 CSA020605.1 CSA017525.1 CSA009282.1 CSA023566.1 CSA017616.1 CSA034340.1 CSA032553.1 CSA000036.1 CSA034492.1 CSA015823.1 CSA012532.1 CSA032333.1 CSA034422.1 CSA035914.1 CSA024855.1 CSA016736.1 CSA002108.1 CSA018092.1 CSA028748.1 CSA010519.1 CSA001667.1 CSA015603.1 CSA034775.1 CSA033994.1 CSA000242.1 CSA024921.1 CSA017654.1 CSA000795.1 CSA009889.1 CSA025110.1 CSA018950.1 CSA029580.1 CSA035327.1 CSA026411.1 CSA025026.1 CSA013010.1 CSA015996.1 CSA003990.1 CSA009205.1 CSA003086.1 CSA007312.1 CSA010323.1 CSA028496.1 CSA034513.1 CSA017438.1 CSA002712.1 CSA031127.1 CSA010527.1 CSA035207.1 CSA009025.1 CSA009966.1 CSA012305.1 CSA025613.1 CSA025612.1 CSA014226.1 CSA011668.1 CSA034838.1 CSA019750.1 CSA018159.1 CSA026796.1 CSA002602.1 CSA004930.1 CSA028285.1 CSA008625.1 CSA019996.1 CSA029743.1 CSA004187.1 CSA020117.1 CSA001302.1 CSA011729.1 CSA016186.1 CSA021512.1 CSA036672.1 CSA022347.1 CSA017604.1 CSA034373.1 CSA036476.1 CSA033261.1 CSA036938.1 CSA002335.1 CSA003654.1 CSA018664.1 CSA031477.1 CSA010160.1 CSA035408.1 CSA001272.1 CSA022831.1 CSA011363.1 CSA024857.1 CSA035117.1 CSA004395.1 CSA018282.1 CSA013744.1 CSA000918.1 CSA010785.1 CSA012055.1 CSA031781.1 CSA030116.1 CSA026696.1 CSA032885.1 CSA003060.1 CSA024718.1 CSA005660.1 CSA005033.1 CSA017504.1 CSA034381.1 CSA034927.1 CSA018288.1 CSA011054.1 CSA015987.1 CSA017430.1
	binding	87	CSA003232.1 CSA008945.1 CSA027795.1 CSA011928.1 CSA019797.1 CSA020605.1 CSA036590.1 CSA022661.1 CSA009282.1 CSA023566.1 CSA003658.1 CSA015823.1 CSA035044.1 CSA032333.1 CSA011544.1 CSA030616.1 CSA002108.1 CSA018092.1 CSA005422.1 CSA028748.1 CSA035465.1 CSA001667.1 CSA033994.1 CSA024921.1 CSA035382.1 CSA000795.1 CSA009889.1 CSA018950.1 CSA030920.1 CSA029580.1 CSA026411.1 CSA026073.1 CSA006518.1 CSA016043.1 CSA002945.1 CSA009205.1 CSA028496.1 CSA002712.1 CSA031127.1 CSA028657.1 CSA009025.1 CSA012305.1 CSA014226.1 CSA025613.1 CSA032664.1 CSA011668.1 CSA026796.1 CSA008625.1 CSA028285.1 CSA019996.1 CSA029266.1 CSA004187.1 CSA036932.1 CSA016186.1 CSA033261.1 CSA036938.1 CSA016400.1 CSA018664.1 CSA031477.1 CSA010160.1 CSA035408.1 CSA001272.1 CSA011363.1 CSA022360.1 CSA004395.1 CSA004105.1 CSA018282.1 CSA007724.1 CSA019651.1 CSA013744.1 CSA000918.1 CSA036936.1 CSA031781.1 CSA000627.1 CSA003662.1 CSA026696.1 CSA003060.1 CSA005660.1 CSA005033.1 CSA013547.1 CSA034381.1 CSA034927.1 CSA015987.1 CSA017430.1 CSA033259.1 CSA008431.1 CSA013811.1
	antioxidant activity	6	CSA035408.1 CSA001272.1 CSA018288.1 CSA018282.1 CSA012305.1 CSA010519.1
	structural molecule activity	4	CSA030434.1 CSA006911.1 CSA036240.1 CSA020098.1
	electron carrier activity	3	CSA022661.1 CSA035117.1 CSA004395.1
	other	3	CSA033994.1 CSA000036.1 CSA030920.1

Subcellular Location	No. of Protein	Protein IDs
chloroplast	79	CSA019572.1 CSA026821.1 CSA008945.1 CSA002124.1 CSA021957.1 CSA020605.1 CSA022661.1 CSA003658.1 CSA025704.1 CSA034492.1 CSA016104.1 CSA015823.1 CSA012532.1 CSA035044.1 CSA006839.1 CSA030616.1 CSA002108.1 CSA018092.1 CSA008456.1 CSA028748.1 CSA010519.1 CSA015603.1 CSA034775.1 CSA033864.1 CSA017654.1 CSA000795.1 CSA025110.1 CSA018950.1 CSA026411.1 CSA026073.1 CSA016043.1 CSA014904.1 CSA002945.1 CSA003086.1 CSA010323.1 CSA002712.1 CSA031127.1 CSA010527.1 CSA004535.1 CSA009025.1 CSA009966.1 CSA012305.1 CSA006911.1 CSA014226.1 CSA010862.1 CSA034838.1 CSA019750.1 CSA030124.1 CSA004532.1 CSA018159.1 CSA005966.1 CSA016587.1 CSA020587.1 CSA026796.1 CSA008625.1 CSA004187.1 CSA036932.1 CSA011729.1 CSA036672.1 CSA020093.1 CSA034373.1 CSA004763.1 CSA033261.1 CSA002335.1 CSA010160.1 CSA035117.1 CSA024064.1 CSA018927.1 CSA033307.1 CSA007724.1 CSA013744.1 CSA029723.1 CSA000627.1 CSA003662.1 CSA005866.1 CSA005660.1 CSA018288.1 CSA011054.1 CSA033259.1
cytoplasm	71	CSA023611.1 CSA004197.1 CSA027795.1 CSA011928.1 CSA008160.1 CSA019797.1 CSA017525.1 CSA036590.1 CSA023566.1 CSA017616.1 CSA032553.1 CSA035914.1 CSA016736.1 CSA005422.1 CSA020775.1 CSA035465.1 CSA003818.1 CSA001667.1 CSA000242.1 CSA024921.1 CSA030920.1 CSA029580.1 CSA035327.1 CSA025026.1 CSA030434.1 CSA003990.1 CSA007312.1 CSA028496.1 CSA017438.1 CSA035207.1 CSA011668.1 CSA002602.1 CSA004930.1 CSA029266.1 CSA029743.1 CSA001302.1 CSA021512.1 CSA017604.1 CSA021163.1 CSA036938.1 CSA003654.1 CSA016400.1 CSA018664.1 CSA030275.1 CSA031477.1 CSA022831.1 CSA011363.1 CSA024857.1 CSA001213.1 CSA018928.1 CSA018282.1 CSA020098.1 CSA019651.1 CSA012055.1 CSA010785.1 CSA000918.1 CSA026696.1 CSA030116.1 CSA031781.1 CSA036240.1 CSA003060.1 CSA032885.1 CSA024718.1 CSA017504.1 CSA013547.1 CSA034381.1 CSA015987.1 CSA017430.1 CSA011328.1 CSA013811.1 CSA008431.1
mitochondria	17	CSA025613.1 CSA032664.1 CSA004395.1 CSA033994.1 CSA009367.1 CSA004105.1 CSA019996.1 CSA009282.1 CSA020117.1 CSA016186.1 CSA021473.1 CSA035053.1 CSA036936.1 CSA006518.1 CSA015996.1 CSA009205.1 CSA034422.1
nucleus	8	CSA028657.1 CSA008457.1 CSA022360.1 CSA013010.1 CSA011544.1 CSA036476.1 CSA024855.1 CSA035382.1
cytoskeleton	6	CSA005033.1 CSA003232.1 CSA034340.1 CSA034927.1 CSA029934.1 CSA009889.1
peroxisome	3	CSA035408.1 CSA001272.1 CSA034513.1
endoplasmic reticulum	2	CSA025612.1 CSA022347.1
chloroplast,mitochondria	2	CSA032333.1 CSA028285.1
vacuolar membrane	1	CSA000036.1
cytoplasm,nucleus	1	CSA028976.1
plasma membrane	1	CSA031259.1

Table S4. The information of GO, KEGG enrichment and protein domain.

GO Terms Level 1	GO Terms Description	GO Terms Level	GO Terms ID	Mapping	Background	All Mapping	All Background	Fold Enrichment	Fisher' exact test P value	-log10(p value)	Related Protein
Molecular Function	catalytic activity	2	GO:0003824	116	8737	153	17667	1.53	2.90536E-11	10.54	CSA026821.1 CSA004197.1 CSA027795.1 CSA011928.1 CSA009367.1 CSA020605.1 CSA017525.1 CSA009282.1 CSA017616.1 CSA023566.1 CSA034340.1 CSA032553.1 CSA000036.1 CSA034492.1 CSA012532.1 CSA015823.1 CSA032333.1 CSA034422.1 CSA016736.1 CSA035914.1 CSA024855.1 CSA002108.1 CSA018092.1 CSA028748.1 CSA010519.1 CSA001667.1 CSA015603.1 CSA033994.1 CSA034775.1 CSA000242.1 CSA024921.1 CSA009889.1 CSA000795.1 CSA017654.1 CSA018950.1 CSA025110.1 CSA029580.1 CSA035327.1 CSA026411.1 CSA025026.1 CSA013010.1 CSA015996.1 CSA003990.1 CSA009205.1 CSA003086.1 CSA010323.1 CSA007312.1 CSA028496.1 CSA017438.1 CSA034513.1 CSA002712.1 CSA031127.1 CSA010527.1 CSA035207.1 CSA009966.1 CSA012305.1 CSA009025.1 CSA014226.1 CSA025613.1 CSA025612.1 CSA011668.1 CSA019750.1 CSA034838.1 CSA018159.1 CSA002602.1 CSA004930.1 CSA026796.1 CSA008625.1 CSA028285.1 CSA019996.1 CSA004187.1 CSA029743.1 CSA020117.1 CSA001302.1 CSA011729.1 CSA016186.1 CSA021512.1 CSA017604.1 CSA022347.1 CSA036672.1 CSA034373.1 CSA033261.1 CSA036476.1 CSA036938.1 CSA002335.1 CSA003654.1 CSA018664.1 CSA031477.1 CSA010160.1 CSA035408.1 CSA001272.1 CSA022831.1 CSA011363.1 CSA024857.1 CSA035117.1 CSA004395.1 CSA018282.1 CSA013744.1 CSA000918.1 CSA012055.1 CSA010785.1 CSA026696.1 CSA031781.1 CSA030116.1 CSA003060.1 CSA032885.1 CSA024718.1 CSA017504.1 CSA005660.1 CSA005033.1 CSA034381.1 CSA034927.1 CSA018288.1 CSA011054.1 CSA015987.1 CSA017430.1
	cofactor binding	3	GO:0048037	23	473	153	17667	5.61	1.15009E-10	9.94	CSA009025.1 CSA014226.1 CSA025613.1 CSA011668.1 CSA027795.1 CSA024921.1 CSA026796.1 CSA028285.1 CSA009889.1 CSA018950.1 CSA004187.1 CSA016186.1 CSA013744.1 CSA000918.1 CSA031781.1 CSA015823.1 CSA032333.1 CSA009205.1 CSA034927.1 CSA018092.1 CSA017430.1 CSA031127.1 CSA010160.1
	oxidoreductase activity	3	GO:0016491	40	1640	153	17667	2.82	2.98087E-09	8.53	CSA009025.1 CSA012305.1 CSA014226.1 CSA011668.1 CSA027795.1 CSA034838.1 CSA004930.1 CSA026796.1 CSA020117.1 CSA001302.1 CSA017616.1 CSA016186.1 CSA034340.1 CSA021512.1 CSA015823.1 CSA032333.1 CSA036476.1 CSA016736.1 CSA003654.1 CSA031477.1 CSA010160.1 CSA035408.1 CSA001272.1 CSA010519.1 CSA035117.1 CSA004395.1 CSA034775.1 CSA000242.1 CSA018282.1 CSA025110.1 CSA018950.1 CSA030116.1 CSA003060.1 CSA013010.1 CSA015996.1 CSA009205.1 CSA018288.1 CSA010323.1 CSA003086.1 CSA011054.1
	isomerase activity	3	GO:0016853	13	218	153	17667	6.89	4.11085E-07	6.39	CSA011729.1 CSA024857.1 CSA015603.1 CSA019750.1 CSA033261.1 CSA002602.1 CSA034927.1 CSA035914.1 CSA007312.1 CSA002335.1 CSA017525.1 CSA018092.1 CSA017438.1
	coenzyme binding	4	GO:0050662	15	315	153	17667	5.5	5.87007E-07	6.23	CSA016186.1 CSA009025.1 CSA014226.1 CSA031781.1 CSA015823.1 CSA011668.1 CSA027795.1 CSA032333.1 CSA026796.1 CSA009205.1 CSA034927.1 CSA018950.1 CSA017430.1 CSA031127.1 CSA010160.1
	oxidoreductase activity, acting on the aldehyde or oxo group of donors	4	GO:0016903	7	56	153	17667	14.43	8.36578E-06	5.08	CSA016186.1 CSA032333.1 CSA034340.1 CSA026796.1 CSA030116.1 CSA018950.1 CSA011668.1
	NAD binding	6	GO:0051287	8	95	153	17667	9.72	1.78258E-05	4.75	CSA016186.1 CSA026796.1 CSA034927.1 CSA009025.1 CSA031781.1 CSA015823.1 CSA018950.1 CSA011668.1
	pyridoxal phosphate binding	5	GO:0030170	8	106	153	17667	8.71	3.63353E-05	4.44	CSA013744.1 CSA000918.1 CSA025613.1 CSA024921.1 CSA028285.1 CSA009889.1 CSA018092.1 CSA004187.1
Cellular Component	intracellular	4	GO:0005622	36	2182	39	4311	1.82	4.41502E-08	7.36	CSA023611.1 CSA028657.1 CSA014226.1 CSA006911.1 CSA032664.1 CSA008945.1 CSA018159.1 CSA008625.1 CSA036590.1 CSA011729.1 CSA023566.1 CSA033261.1 CSA018664.1 CSA030275.1 CSA028748.1 CSA022360.1 CSA001667.1 CSA033994.1 CSA033864.1 CSA004105.1 CSA020098.1 CSA029580.1 CSA030920.1 CSA007724.1 CSA019651.1 CSA029723.1 CSA000627.1 CSA036240.1 CSA005660.1 CSA006518.1 CSA030434.1 CSA003990.1 CSA034381.1 CSA034513.1 CSA002712.1 CSA033259.1
	cell	2	GO:0005623	36	2336	39	4311	1.7	3.95724E-07	6.4	CSA023611.1 CSA028657.1 CSA014226.1 CSA006911.1 CSA032664.1 CSA008945.1 CSA018159.1 CSA008625.1 CSA036590.1 CSA011729.1 CSA023566.1 CSA033261.1 CSA018664.1 CSA030275.1 CSA028748.1 CSA022360.1 CSA001667.1 CSA033994.1 CSA033864.1 CSA004105.1 CSA020098.1 CSA029580.1 CSA030920.1 CSA007724.1 CSA019651.1 CSA029723.1 CSA000627.1 CSA036240.1 CSA005660.1 CSA006518.1 CSA030434.1 CSA003990.1 CSA034381.1 CSA034513.1 CSA002712.1 CSA033259.1
	cell part	3	GO:0044464	36	2336	39	4311	1.7	3.95724E-07	6.4	CSA023611.1 CSA028657.1 CSA014226.1 CSA006911.1 CSA032664.1 CSA008945.1 CSA018159.1 CSA008625.1 CSA036590.1 CSA011729.1 CSA023566.1 CSA033261.1 CSA018664.1 CSA030275.1 CSA028748.1 CSA022360.1 CSA001667.1 CSA033994.1 CSA033864.1 CSA004105.1 CSA020098.1 CSA029580.1 CSA030920.1 CSA007724.1 CSA019651.1 CSA029723.1 CSA000627.1 CSA036240.1 CSA005660.1 CSA006518.1 CSA030434.1 CSA003990.1 CSA034381.1 CSA034513.1 CSA002712.1 CSA033259.1
	cytoplasm	6	GO:0005737	24	1079	39	4311	2.46	3.37566E-06	5.47	CSA014226.1 CSA006911.1 CSA008945.1 CSA001667.1 CSA033994.1 CSA018159.1 CSA008625.1 CSA020098.1 CSA036590.1 CSA029580.1 CSA019651.1 CSA011729.1 CSA023566.1 CSA029723.1 CSA036240.1 CSA005660.1 CSA006518.1 CSA030434.1 CSA003990.1 CSA033261.1 CSA034381.1 CSA018664.1 CSA002712.1 CSA030275.1
	intracellular part	5	GO:0044424	32	2049	39	4311	1.73	1.53087E-05	4.82	CSA023611.1 CSA028657.1 CSA014226.1 CSA006911.1 CSA008945.1 CSA018159.1 CSA008625.1 CSA036590.1 CSA011729.1 CSA023566.1 CSA033261.1 CSA018664.1 CSA030275.1 CSA022360.1 CSA001667.1 CSA033994.1 CSA033864.1 CSA020098.1 CSA029580.1 CSA030920.1 CSA019651.1 CSA029723.1 CSA000627.1 CSA036240.1 CSA005660.1 CSA006518.1 CSA030434.1 CSA034381.1 CSA003990.1 CSA034513.1 CSA002712.1 CSA033259.1
	catalytic complex	3	GO:1902494	7	239	39	4311	3.24	0.01688336	1.77	CSA034381.1 CSA029723.1 CSA000627.1 CSA002712.1 CSA034513.1 CSA033259.1 CSA030920.1
	oxidoreductase complex	4	GO:1990204	3	36	39	4311	9.21	0.039494371	1.4	CSA029723.1 CSA000627.1 CSA033259.1
	phosphopyruvate hydratase complex	10	GO:0000015	2	5	39	4311	44.22	0.043322886	1.36	CSA034381.1 CSA002712.1
Biological Process	organic acid metabolic process	5	GO:0006082	34	612	131	11709	4.97	3.02536E-13	12.52	CSA035207.1 CSA009966.1 CSA009025.1 CSA014226.1 CSA025613.1 CSA019750.1 CSA008625.1 CSA019996.1 CSA017525.1 CSA004187.1 CSA020117.1 CSA017616.1 CSA023566.1 CSA032333.1 CSA003654.1 CSA018664.1 CSA015603.1 CSA004395.1 CSA034775.1 CSA000242.1 CSA009889.1 CSA013744.1 CSA010785.1 CSA029723.1 CSA026696.1 CSA031781.1 CSA032885.1 CSA017504.1 CSA015996.1 CSA003990.1 CSA034381.1 CSA009205.1 CSA003086.1 CSA002712.1
	oxoacid metabolic process	6	GO:0043436	34	611	131	11709	4.97	7.34635E-13	12.13	CSA035207.1 CSA009966.1 CSA009025.1 CSA014226.1 CSA025613.1 CSA019750.1 CSA008625.1 CSA019996.1 CSA017525.1 CSA004187.1 CSA020117.1 CSA017616.1 CSA023566.1 CSA032333.1 CSA003654.1 CSA018664.1 CSA015603.1 CSA004395.1 CSA034775.1 CSA000242.1 CSA009889.1 CSA013744.1 CSA010785.1 CSA029723.1 CSA026696.1 CSA031781.1 CSA032885.1 CSA017504.1 CSA015996.1 CSA003990.1 CSA034381.1 CSA009205.1 CSA003086.1 CSA002712.1
	carboxylic acid metabolic process	7	GO:0019752	34	597	131	11709	5.09	9.70002E-13	12.01	CSA035207.1 CSA009966.1 CSA009025.1 CSA014226.1 CSA025613.1 CSA019750.1 CSA008625.1 CSA019996.1 CSA017525.1 CSA004187.1 CSA020117.1 CSA017616.1 CSA023566.1 CSA032333.1 CSA003654.1 CSA018664.1 CSA015603.1 CSA004395.1 CSA034775.1 CSA000242.1 CSA009889.1 CSA013744.1 CSA010785.1 CSA029723.1 CSA026696.1 CSA031781.1 CSA032885.1 CSA017504.1 CSA015996.1 CSA003990.1 CSA034381.1 CSA009205.1 CSA003086.1 CSA002712.1
	small molecule metabolic process	4	GO:0044281	44	943	131	11709	4.17	1.09901E-12	11.96	CSA035207.1 CSA009966.1 CSA009025.1 CSA014226.1 CSA025613.1 CSA011668.1 CSA019750.1 CSA034838.1 CSA026796.1 CSA008625.1 CSA019996.1 CSA017525.1 CSA004187.1 CSA020117.1 CSA017616.1 CSA036932.1 CSA023566.1 CSA032333.1 CSA033261.1 CSA035914.1 CSA003654.1 CSA018664.1 CSA024857.1 CSA004395.1 CSA015603.1 CSA033994.1 CSA034775.1 CSA000242.1 CSA009889.1 CSA018950.1 CSA013744.1 CSA010785.1 CSA029723.1 CSA031781.1 CSA026696.1 CSA032885.1 CSA017504.1 CSA015996.1 CSA003990.1 CSA034381.1 CSA009205.1 CSA003086.1 CSA011054.1 CSA002712.1
	single-organism metabolic process	3	GO:0044710	69	2860	131	11709	2.16	5.82788E-12	11.23	CSA027795.1 CSA020605.1 CSA017525.1 CSA017616.1 CSA023566.1 CSA034340.1 CSA015823.1 CSA032333.1 CSA034422.1 CSA016736.1 CSA035914.1 CSA010519.1 CSA015603.1 CSA033994.1 CSA034775.1 CSA000242.1 CSA009889.1 CSA025110.1 CSA018950.1 CSA015996.1 CSA003990.1 CSA009205.1 CSA003086.1 CSA007312.1 CSA028496.1 CSA017438.1 CSA002712.1 CSA035207.1 CSA012305.1 CSA009025.1 CSA009966.1 CSA025613.1 CSA014226.1 CSA011668.1 CSA034838.1 CSA019750.1 CSA004930.1 CSA026796.1 CSA008625.1 CSA019996.1 CSA004187.1 CSA001302.1 CSA020117.1 CSA036932.1 CSA016186.1 CSA021512.1 CSA033261.1 CSA036938.1 CSA018664.1 CSA003654.1 CSA031477.1 CSA010160.1 CSA035408.1 CSA001272.1 CSA024857.1 CSA004395.1 CSA018282.1 CSA013744.1 CSA010785.1 CSA029723.1 CSA030116.1 CSA031781.1 CSA026696.1 CSA032885.1 CSA003060.1 CSA017504.1 CSA034381.1 CSA018288.1 CSA011054.1
	single-organism cellular process	3	GO:0044763	50	1885	131	11709	2.37	1.87283E-09	8.73	CSA035207.1 CSA028657.1 CSA009966.1 CSA009025.1 CSA014226.1 CSA025613.1 CSA019750.1 CSA034838.1 CSA008625.1 CSA019996.1 CSA017525.1 CSA004187.1 CSA020117.1 CSA017616.1 CSA036932.1 CSA023566.1 CSA032333.1 CSA034422.1 CSA003654.1 CSA018664.1 CSA016400.1 CSA010160.1 CSA010519.1 CSA024857.1 CSA035117.1 CSA004395.1 CSA015603.1 CSA033994.1 CSA034775.1 CSA000242.1 CSA009889.1 CSA013744.1 CSA029723.1 CSA010785.1 CSA008457.1 CSA031781.1 CSA026696.1 CSA032885.1 CSA017504.1 CSA015996.1 CSA003990.1 CSA034381.1 CSA009205.1 CSA003086.1 CSA018288.1 CSA007312.1 CSA028496.1 CSA011054.1 CSA002712.1 CSA017438.1
	single-organism process	2	GO:0044699	77	3982	131	11709	1.73	8.53503E-09	8.07	CSA027795.1 CSA020605.1 CSA017525.1 CSA017616.1 CSA023566.1 CSA034340.1 CSA000036.1 CSA015823.1 CSA032333.1 CSA034422.1 CSA016736.1 CSA035914.1 CSA002108.1 CSA010519.1 CSA015603.1 CSA033994.1 CSA034775.1 CSA000242.1 CSA009889.1 CSA025110.1 CSA018950.1 CSA008457.1 CSA015996.1 CSA003990.1 CSA009205.1 CSA003086.1 CSA007312.1 CSA028496.1 CSA017438.1 CSA002712.1 CSA028657.1 CSA035207.1 CSA012305.1 CSA009025.1 CSA009966.1 CSA025613.1 CSA014226.1 CSA032664.1 CSA011668.1 CSA034838.1 CSA019750.1 CSA004930.1 CSA026796.1 CSA008625.1 CSA019996.1 CSA004187.1 CSA001302.1 CSA020117.1 CSA036932.1 CSA016186.1 CSA021512.1 CSA033261.1 CSA036938.1 CSA016400.1 CSA018664.1 CSA003654.1 CSA031477.1 CSA010160.1 CSA035408.1 CSA001272.1 CSA024857.1 CSA035117.1 CSA004395.1 CSA004105.1 CSA018282.1 CSA013744.1 CSA029723.1 CSA010785.1 CSA031781.1 CSA030116.1 CSA026696.1 CSA032885.1 CSA003060.1 CSA017504.1 CSA034381.1 CSA018288.1 CSA011054.1
	organonitrogen compound metabolic process	4	GO:1901564	38	1280	131	11709	2.65	2.6215E-08	7.58	CSA003232.1 CSA010527.1 CSA035207.1 CSA009966.1 CSA025613.1 CSA006911.1 CSA019750.1 CSA034838.1 CSA008625.1 CSA019996.1 CSA017525.1 CSA004187.1 CSA020117.1 CSA036932.1 CSA023566.1 CSA003654.1 CSA018092.1 CSA018664.1 CSA028748.1 CSA001667.1 CSA015603.1 CSA033994.1 CSA009889.1 CSA020098.1 CSA007724.1 CSA013744.1 CSA010785.1 CSA029723.1 CSA031781.1 CSA026696.1 CSA036240.1 CSA030434.1 CSA015996.1 CSA003990.1 CSA034381.1 CSA003086.1 CSA028496.1 CSA002712.1
	glycine metabolic process	11	GO:0006544	7	20	131	11709	31.28	5.69141E-08	7.24	CSA020117.1 CSA013744.1 CSA015996.1 CSA029723.1 CSA025613.1 CSA009889.1 CSA019996.1
	single-organism catabolic process	4	GO:0044712	13	155	131	11709	7.5	1.42028E-07	6.85	CSA020117.1 CSA035207.1 CSA029723.1 CSA009966.1 CSA031781.1 CSA015603.1 CSA019750.1 CSA015996.1 CSA033261.1 CSA034381.1 CSA019996.1 CSA002712.1 CSA017525.1
	single-organism carbohydrate metabolic process	5	GO:0044723	16	263	131	11709	5.44	2.10983E-07	6.68	CSA035207.1 CSA009966.1 CSA024857.1 CSA011668.1 CSA015603.1 CSA019750.1 CSA026796.1 CSA017525.1 CSA018950.1 CSA034422.1 CSA034381.1 CSA033261.1 CSA007312.1 CSA035914.1 CSA002712.1 CSA017438.1
	cellular amino acid metabolic process	8	GO:0006520	17	317	131	11709	4.79	4.2596E-07	6.37	CSA025613.1 CSA008625.1 CSA009889.1 CSA019996.1 CSA004187.1 CSA020117.1 CSA023566.1 CSA013744.1 CSA029723.1 CSA010785.1 CSA031781.1 CSA026696.1 CSA015996.1 CSA003990.1 CSA003086.1 CSA018664.1 CSA003654.1
	generation of precursor metabolites and energy	4	GO:0006091	12	148	131	11709	7.25	7.26835E-07	6.14	CSA035207.1 CSA009966.1 CSA032885.1 CSA004395.1 CSA015603.1 CSA019750.1 CSA017504.1 CSA032333.1 CSA034381.1 CSA009205.1 CSA002712.1 CSA017525.1
	alpha-amino acid metabolic process	9	GO:1901605	12	155	131	11709	6.92	1.15172E-06	5.94	CSA020117.1 CSA013744.1 CSA010785.1 CSA029723.1 CSA025613.1 CSA026696.1 CSA031781.1 CSA015996.1 CSA009889.1 CSA003654.1 CSA019996.1 CSA004187.1

KEGG pathway	Mapping	Background	All Mapping	All Background	Fold enrichment	Fisher's exact test p value	-log10(p value)	Related proteins
ath00630 Glyoxylate and dicarboxylate metabolism	22	58	116	3267	10.68	5.39568E-14	13.27	CSA035408.1 CSA001272.1 CSA022831.1 CSA025613.1 CSA034775.1 CSA000242.1 CSA024921.1 CSA020587.1 CSA009889.1 CSA019996.1 CSA020117.1 CSA016186.1 CSA029723.1 CSA025704.1 CSA010785.1 CSA000918.1 CSA017504.1 CSA004763.1 CSA015996.1 CSA036938.1 CSA003654.1 CSA010160.1
ath00710 Carbon fixation in photosynthetic organisms	13	57	116	3267	9.39	2.08389E-13	12.68	CSA035207.1 CSA009966.1 CSA015603.1 CSA019750.1 CSA026796.1 CSA020587.1 CSA000795.1 CSA017654.1 CSA018950.1 CSA034492.1 CSA012532.1 CSA004763.1 CSA036938.1
ath00020 Citrate cycle (TCA cycle)	9	40	116	3267	6.34	5.53517E-05	4.26	CSA025704.1 CSA004395.1 CSA017504.1 CSA034775.1 CSA034373.1 CSA000242.1 CSA032333.1 CSA009205.1 CSA010160.1
ath00260 Glycine, serine and threonine metabolism	10	57	116	3267	4.94	0.000131718	3.88	CSA020117.1 CSA029723.1 CSA000918.1 CSA025613.1 CSA030116.1 CSA015996.1 CSA024921.1 CSA009889.1 CSA019996.1 CSA010160.1
ath00196 Photosynthesis - antenna proteins	6	16	116	3267	10.56	0.000158607	3.8	CSA019572.1 CSA004532.1 CSA016587.1 CSA010862.1 CSA011328.1 CSA024064.1
ath00010 Glycolysis / Gluconeogenesis	11	85	116	3267	3.64	0.000665951	3.18	CSA034340.1 CSA035207.1 CSA009966.1 CSA030116.1 CSA015603.1 CSA019750.1 CSA034381.1 CSA017654.1 CSA002712.1 CSA017525.1 CSA010160.1 CSA018950.1 CSA026796.1
ath00030 Pentose phosphate pathway	7	45	116	3267	4.38	0.004426525	2.35	CSA034838.1 CSA034340.1 CSA035207.1 CSA009966.1 CSA017654.1 CSA017525.1 CSA012532.1
ath00053 Ascorbate and aldarate metabolism	6	37	116	3267	4.57	0.008769056	2.06	CSA032553.1 CSA034927.1 CSA018282.1 CSA030116.1 CSA035044.1 CSA027795.1
ath00620 Pyruvate metabolism	7	67	116	3267	2.94	0.029036969	1.54	CSA034775.1 CSA000242.1 CSA017616.1 CSA011363.1 CSA009025.1 CSA030116.1 CSA010160.1
ath00910 Nitrogen metabolism	4	21	116	3267	5.36	0.035599895	1.45	CSA022831.1 CSA010785.1 CSA002108.1 CSA003654.1

Domain Description	Mapping	Background	All Mapping	All Backgroud	Fold enrichment	Fisher's exact test p value	-log10(P value)	Related Protein
NAD(P)-binding domain	23	578	175	22761	5.18	6.4575E-10	9.19	CSA009025.1 CSA024857.1 CSA011668.1 CSA034838.1 CSA034775.1 CSA000242.1 CSA026796.1 CSA021957.1 CSA018950.1 CSA016186.1 CSA017616.1 CSA031781.1 CSA013010.1 CSA034373.1 CSA014904.1 CSA036476.1 CSA034927.1 CSA035914.1 CSA010323.1 CSA003086.1 CSA017430.1 CSA031477.1 CSA031127.1
Pyridoxal phosphate-dependent transferase, major region, subdomain 1	10	122	175	22761	10.66	4.21661E-07	6.38	CSA020117.1 CSA013744.1 CSA000918.1 CSA025613.1 CSA015996.1 CSA024921.1 CSA028285.1 CSA009889.1 CSA018092.1 CSA004187.1
Pyridoxal phosphate-dependent transferase	10	134	175	22761	9.71	9.32568E-07	6.03	CSA020117.1 CSA013744.1 CSA000918.1 CSA025613.1 CSA015996.1 CSA024921.1 CSA028285.1 CSA009889.1 CSA018092.1 CSA004187.1
Chlorophyll a/b binding protein domain	6	31	175	22761	25.17	3.56492E-06	5.45	CSA019572.1 CSA004532.1 CSA016587.1 CSA010862.1 CSA011328.1 CSA024064.1
Pyridoxal phosphate-dependent transferase, major region, subdomain 2	8	91	175	22761	11.43	6.37697E-06	5.2	CSA013744.1 CSA000918.1 CSA025613.1 CSA024921.1 CSA028285.1 CSA009889.1 CSA018092.1 CSA004187.1
Aldolase-type TIM barrel	7	125	175	22761	7.28	0.000405234	3.39	CSA019750.1 CSA035207.1 CSA024855.1 CSA009966.1 CSA004197.1 CSA003654.1 CSA015603.1
Ribulose bisphosphate carboxylase, large subunit, C-terminal	3	6	175	22761	65.03	0.000854194	3.07	CSA036938.1 CSA036936.1 CSA020605.1
GroEL-like apical domain	4	29	175	22761	17.94	0.001386246	2.86	CSA006518.1 CSA035465.1 CSA008945.1 CSA036590.1
GroEL-like equatorial domain	4	40	175	22761	13.01	0.003524728	2.45	CSA006518.1 CSA035465.1 CSA008945.1 CSA036590.1
Glyceraldehyde 3-phosphate dehydrogenase, catalytic domain	3	12	175	22761	32.52	0.003646691	2.44	CSA026796.1 CSA018950.1 CSA011668.1
Glyceraldehyde 3-phosphate dehydrogenase, NAD(P) binding domain	3	13	175	22761	30.01	0.004288142	2.37	CSA026796.1 CSA018950.1 CSA011668.1
Thioredoxin-like fold	8	274	175	22761	3.8	0.005243716	2.28	CSA003232.1 CSA008457.1 CSA010519.1 CSA035117.1 CSA035044.1 CSA030616.1 CSA018288.1 CSA011054.1
Lactate/malate dehydrogenase, N-terminal	3	16	175	22761	24.39	0.006498644	2.19	CSA034775.1 CSA017616.1 CSA000242.1
Lactate/malate dehydrogenase, C-terminal	3	20	175	22761	19.51	0.010085632	2	CSA034775.1 CSA017616.1 CSA000242.1
Lactate dehydrogenase/glycoside hydrolase, family 4, C-terminal	3	21	175	22761	18.58	0.011091727	1.96	CSA034775.1 CSA017616.1 CSA000242.1
Translation elongation factor EFTu-like, domain 2	3	24	175	22761	16.26	0.014361115	1.84	CSA028748.1 CSA001667.1 CSA011928.1
Ribulose-1,5-bisphosphate carboxylase small subunit, N-terminal	2	2	175	22761	130.06	0.015231203	1.82	CSA004763.1 CSA020587.1
Ribosomal protein S5 domain 2-type fold	4	75	175	22761	6.94	0.019842429	1.7	CSA013547.1 CSA036240.1 CSA013811.1 CSA011928.1
Ribulose bisphosphate carboxylase small chain, domain	2	3	175	22761	86.71	0.022760083	1.64	CSA004763.1 CSA020587.1
Transcription factor, GTP-binding domain	3	31	175	22761	12.59	0.023367427	1.63	CSA028748.1 CSA001667.1 CSA011928.1
Thioredoxin domain	4	83	175	22761	6.27	0.025838111	1.59	CSA008457.1 CSA018288.1 CSA010519.1 CSA011054.1
Catalase immune-responsive domain	2	4	175	22761	65.03	0.03023173	1.52	CSA035408.1 CSA001272.1
Ribulose bisphosphate carboxylase, large subunit, ferrodoxin-like N-terminal	2	4	175	22761	65.03	0.03023173	1.52	CSA036938.1 CSA020605.1
Enolase, N-terminal	2	5	175	22761	52.03	0.037646577	1.42	CSA034381.1 CSA002712.1
Enolase, C-terminal TIM barrel domain	2	5	175	22761	52.03	0.037646577	1.42	CSA034381.1 CSA002712.1

Table S5. The information of S-nitrosylated proteins in multiple metabolic pathways .

Glycolysis

Protein accession	Position	Amino acid	Protein description	Subcellular localization	KEGG KO No.	KEGG Gene
CSA017525.1	8	C	"Glucose-6-phosphate isomerase, cytosolic 1 OS=Clarkia xantiana GN=PGIC1 PE=3 SV=1"	cytoplasm	K01810	GPI; glucose-6-phosphate isomerase [EC:5.3.1.9]
CSA017654.1	365	C	"Fructose-1,6-bisphosphatase, chloroplastic OS=Pisum sativum GN=FBP PE=1 SV=2"	chloroplast	K03841	FBP; fructose-1,6-bisphosphatase I [EC:3.1.3.11]
CSA009966.1	276	C	"Fructose-bisphosphate aldolase 2, chloroplastic OS=Arabidopsis thaliana GN=FBA2 PE=1 SV=2"	chloroplast	K01623	ALDO; fructose-bisphosphate aldolase, class I [EC:4.1.2.13]
CSA009966.1	162	C	"Fructose-bisphosphate aldolase 2, chloroplastic OS=Arabidopsis thaliana GN=FBA2 PE=1 SV=2"	chloroplast	K01623	ALDO; fructose-bisphosphate aldolase, class I [EC:4.1.2.13]
CSA009966.1	78	C	"Fructose-bisphosphate aldolase 2, chloroplastic OS=Arabidopsis thaliana GN=FBA2 PE=1 SV=2"	chloroplast	K01623	ALDO; fructose-bisphosphate aldolase, class I [EC:4.1.2.13]
CSA035207.1	246	C	"Fructose-bisphosphate aldolase, cytoplasmic isozyme OS=Cicer arietinum GN=ALDC PE=2 SV=1"	cytoplasm	K01623	ALDO; fructose-bisphosphate aldolase, class I [EC:4.1.2.13]
CSA015603.1	202	C	"Triosephosphate isomerase, chloroplastic OS=Fragaria ananassa GN=TPI PE=2 SV=1"	chloroplast	K01803	TPI; triosephosphate isomerase (TIM) [EC:5.3.1.1]
CSA019750.1	67	C	"Triosephosphate isomerase, cytosolic OS=Petunia hybrida GN=TPIP1 PE=2 SV=1"	chloroplast	K01803	TPI; triosephosphate isomerase (TIM) [EC:5.3.1.1]
CSA019750.1	165	C	"Triosephosphate isomerase, cytosolic OS=Petunia hybrida GN=TPIP1 PE=2 SV=1"	chloroplast	K01803	TPI; triosephosphate isomerase (TIM) [EC:5.3.1.1]
CSA019750.1	153	C	"Triosephosphate isomerase, cytosolic OS=Petunia hybrida GN=TPIP1 PE=2 SV=1"	chloroplast	K01803	TPI; triosephosphate isomerase (TIM) [EC:5.3.1.1]
CSA034340.1	50	C	NADP-dependent glyceraldehyde-3-phosphate dehydrogenase OS=Nicotiana plumbaginifolia GN=GAPN PE=2 SV=1	cytoskeleton	K00131	gapN; glyceraldehyde-3-phosphate dehydrogenase (NADP+) [EC:1.2.1.9]
CSA002712.1	282	C	Enolase 1 OS=Hevea brasiliensis GN=ENO1 PE=1 SV=1	chloroplast	K01689	ENO; enolase [EC:4.2.1.11]
CSA002712.1	107	C	Enolase 1 OS=Hevea brasiliensis GN=ENO1 PE=1 SV=1	chloroplast	K01689	ENO; enolase [EC:4.2.1.11]
CSA034381.1	107	C	Enolase 2 OS=Zea mays GN=ENO2 PE=2 SV=1	cytoplasm	K01689	ENO; enolase [EC:4.2.1.11]
CSA018950.1	367	C	"Glyceraldehyde-3-phosphate dehydrogenase B, chloroplastic OS=Pisum sativum GN=GAPB PE=2 SV=2"	chloroplast	K05298	GAPB; glyceraldehyde-3-phosphate dehydrogenase (NADP+) (phosphorylating) [EC:1.2.1.13]
CSA018950.1	441	C	"Glyceraldehyde-3-phosphate dehydrogenase B, chloroplastic OS=Pisum sativum GN=GAPB PE=2 SV=2"	chloroplast	K05298	GAPB; glyceraldehyde-3-phosphate dehydrogenase (NADP+) (phosphorylating) [EC:1.2.1.13]
CSA026796.1	346	C	"Glyceraldehyde-3-phosphate dehydrogenase A, chloroplastic OS=Spinacia oleracea GN=GAPA PE=1 SV=2"	chloroplast	K05298	GAPA; glyceraldehyde-3-phosphate dehydrogenase (NADP+) (phosphorylating) [EC:1.2.1.13]

Pyruvate metabolism						
**Protein accession**	**Position**	**Amino acid**	**Protein description**	**Subcellular localization**	**KEGG KO No.**	**KEGG Gene**
CSA000242.1	125	C	"Malate dehydrogenase 2, cytoplasmic OS=Arabidopsis thaliana GN=MDH2 PE=1 SV=1"	cytoplasm	K00025	MDH2; malate dehydrogenase [EC:1.1.1.37]
CSA034775.1	516	C	"Malate dehydrogenase 1, mitochondrial OS=Arabidopsis thaliana GN=At1g53240 PE=1 SV=1"	chloroplast	K00026	MDH1; malate dehydrogenase [EC:1.1.1.37]
CSA017616.1	334	C	"Malate dehydrogenase [NADP], chloroplastic OS=Pisum sativum PE=1 SV=2"	cytoplasm	K00051	E1.1.1.82; malate dehydrogenase (NADP+) [EC:1.1.1.82]
CSA017616.1	346	C	"Malate dehydrogenase [NADP], chloroplastic OS=Pisum sativum PE=1 SV=2"	cytoplasm	K00051	E1.1.1.82; malate dehydrogenase (NADP+) [EC:1.1.1.82]
CSA009025.1	548	C	NADP-dependent malic enzyme OS=Vitis vinifera PE=2 SV=1	chloroplast	K00029	E1.1.1.40; malate dehydrogenase (oxaloacetate-decarboxylating)(NADP+) [EC:1.1.1.40]
CSA010160.1	485	C	"Dihydrolipoyl dehydrogenase 1, mitochondrial OS=Arabidopsis thaliana GN=LPD1 PE=1 SV=2"	chloroplast	K00382	DLD; dihydrolipoamide dehydrogenase [EC:1.8.1.4]
CSA030116.1	65	C	Aldehyde dehydrogenase family 7 member B4 OS=Arabidopsis thaliana GN=ALDH7B4 PE=2 SV=3	cytoplasm	K14085	ALDH7A1; aldehyde dehydrogenase family 7 member A1 [EC:1.2.1.31 1.2.1.8 1.2.1.3]
CSA011363.1	44	C	Lactoylglutathione lyase OS=Oryza sativa subsp. japonica GN=GLYI-11 PE=1 SV=2	cytoplasm	K01759	GLO1; lactoylglutathione lyase [EC:4.4.1.5]

TCA cycle						
**Protein accession**	**Position**	**Amino acid**	**Protein description**	**Subcellular localization**	**KEGG KO No.**	**KEGG Gene**
CSA017504.1	438	C	"Citrate synthase, mitochondrial OS=Daucus carota GN=CS PE=2 SV=1"	cytoplasm	K01647	CS; citrate synthase [EC:2.3.3.1]
CSA034373.1	33	C	ATP-citrate synthase beta chain protein 1 OS=Oryza sativa subsp. japonica GN=ACLB-1 PE=2 SV=1	chloroplast	K01648	ACLY; ATP citrate (pro-S)-lyase [EC:2.3.3.8]
CSA025704.1	205	C	"Aconitate hydratase 2, mitochondrial OS=Arabidopsis thaliana GN=ACO2 PE=1 SV=2"	chloroplast	K01681	ACO; aconitate hydratase [EC:4.2.1.3]
CSA032333.1	200	C	"Pyruvate dehydrogenase E1 component subunit alpha-3, chloroplastic OS=Oryza sativa subsp. japonica GN=Os04g0119400 PE=2 SV=2"	oplast,mitocho	K00164	OGDH; 2-oxoglutarate dehydrogenase E1 component [EC:1.2.4.2]
CSA010160.1	485	C	"Dihydrolipoyl dehydrogenase 1, mitochondrial OS=Arabidopsis thaliana GN=LPD1 PE=1 SV=2"	chloroplast	K00382	DLD; dihydrolipoamide dehydrogenase [EC:1.8.1.4]
CSA004395.1	74	C	"Succinate dehydrogenase [ubiquinone] iron-sulfur subunit 2, mitochondrial OS=Arabidopsis thaliana GN=SDH2-2 PE=1 SV=2"	mitochondria	K00235	SDHB; succinate dehydrogenase (ubiquinone) iron-sulfur subunit [EC:1.3.5.1]
CSA009205.1	309	C	"Succinate dehydrogenase [ubiquinone] flavoprotein subunit 1, mitochondrial OS=Arabidopsis thaliana GN=SDH1-1 PE=1 SV=1"	mitochondria	K00234	SDHA; succinate dehydrogenase (ubiquinone) flavoprotein subunit [EC:1.3.5.1]
CSA009205.1	179	C	"Succinate dehydrogenase [ubiquinone] flavoprotein subunit 1, mitochondrial OS=Arabidopsis thaliana GN=SDH1-1 PE=1 SV=1"	mitochondria	K00234	SDHA; succinate dehydrogenase (ubiquinone) flavoprotein subunit [EC:1.3.5.1]
CSA000242.1	125	C	"Malate dehydrogenase 2, cytoplasmic OS=Arabidopsis thaliana GN=MDH2 PE=1 SV=1"	cytoplasm	K00025	MDH2; malate dehydrogenase [EC:1.1.1.37]
CSA034775.1	516	C	"Malate dehydrogenase 1, mitochondrial OS=Arabidopsis thaliana GN=At1g53240 PE=1 SV=1"	chloroplast	K00026	MDH1; malate dehydrogenase [EC:1.1.1.37]

Calvin cycle						
**Protein accession**	**Position**	**Amino acid**	**Protein description**	**Subcellular localization**	**KEGG KO No.**	**KEGG Gene**
CSA012532.1	446	C	"Transketolase, chloroplastic OS=Solanum tuberosum PE=2 SV=1"	chloroplast	K00615	TK; transketolase [EC:2.2.1.1]
CSA012532.1	151	C	"Transketolase, chloroplastic OS=Solanum tuberosum PE=2 SV=1"	chloroplast	K00615	TK; transketolase [EC:2.2.1.1]
CSA009966.1	276	C	"Fructose-bisphosphate aldolase 2, chloroplastic OS=Arabidopsis thaliana GN=FBA2 PE=1 SV=2"	chloroplast	K01623	ALDO; fructose-bisphosphate aldolase, class I [EC:4.1.2.13]
CSA009966.1	162	C	"Fructose-bisphosphate aldolase 2, chloroplastic OS=Arabidopsis thaliana GN=FBA2 PE=1 SV=2"	chloroplast	K01623	ALDO; fructose-bisphosphate aldolase, class I [EC:4.1.2.13]
CSA009966.1	78	C	"Fructose-bisphosphate aldolase 2, chloroplastic OS=Arabidopsis thaliana GN=FBA2 PE=1 SV=2"	chloroplast	K01623	ALDO; fructose-bisphosphate aldolase, class I [EC:4.1.2.13]
CSA035207.1	246	C	"Fructose-bisphosphate aldolase, cytoplasmic isozyme OS=Cicer arietinum GN=ALDC PE=2 SV=1"	cytoplasm	K01623	ALDO; fructose-bisphosphate aldolase, class I [EC:4.1.2.13]
CSA034492.1	97	C	"Sedoheptulose-1,7-bisphosphatase, chloroplastic OS=Spinacia oleracea PE=2 SV=1"	chloroplast	K01100	SBPase; sedoheptulose-bisphosphatase [EC:3.1.3.37]
CSA000795.1	67	C	"Phosphoribulokinase, chloroplastic OS=Triticum aestivum PE=2 SV=1"	chloroplast	K00855	PRK; phosphoribulokinase [EC:2.7.1.19]
CSA000795.1	106	C	"Phosphoribulokinase, chloroplastic OS=Triticum aestivum PE=2 SV=1"	chloroplast	K00855	PRK; phosphoribulokinase [EC:2.7.1.19]
CSA000795.1	295	C	"Phosphoribulokinase, chloroplastic OS=Triticum aestivum PE=2 SV=1"	chloroplast	K00855	PRK; phosphoribulokinase [EC:2.7.1.19]
CSA004763.1	130	C	"Ribulose bisphosphate carboxylase small chain, chloroplastic OS=Betula pendula GN=RBCS1 PE=2 SV=1"	chloroplast	K01602	rbcS; ribulose-bisphosphate carboxylase small chain [EC:4.1.1.39]
CSA004763.1	143	C	"Ribulose bisphosphate carboxylase small chain, chloroplastic OS=Betula pendula GN=RBCS1 PE=2 SV=1"	chloroplast	K01602	rbcS; ribulose-bisphosphate carboxylase small chain [EC:4.1.1.39]
CSA020587.1	97	C	"Ribulose bisphosphate carboxylase small chain, chloroplastic OS=Brassica napus GN=RBCS PE=2 SV=2"	chloroplast	K01602	rbcS; ribulose-bisphosphate carboxylase small chain [EC:4.1.1.39]
CSA036938.1	207	C	Ribulose bisphosphate carboxylase large chain OS=Carpinus caroliniana GN=rbcL PE=3 SV=1	cytoplasm	K01601	rbcL; ribulose-bisphosphate carboxylase large chain [EC:4.1.1.39]
CSA036938.1	262	C	Ribulose bisphosphate carboxylase large chain OS=Carpinus caroliniana GN=rbcL PE=3 SV=1	cytoplasm	K01601	rbcL; ribulose-bisphosphate carboxylase large chain [EC:4.1.1.39]
CSA036938.1	299	C	Ribulose bisphosphate carboxylase large chain OS=Carpinus caroliniana GN=rbcL PE=3 SV=1	cytoplasm	K01601	rbcL; ribulose-bisphosphate carboxylase large chain [EC:4.1.1.39]
CSA018950.1	367	C	"Glyceraldehyde-3-phosphate dehydrogenase B, chloroplastic OS=Pisum sativum GN=GAPB PE=2 SV=2"	chloroplast	K05298	GAPB; glyceraldehyde-3-phosphate dehydrogenase (NADP+) (phosphorylating) [EC:1.2.1.13]
CSA018950.1	441	C	"Glyceraldehyde-3-phosphate dehydrogenase B, chloroplastic OS=Pisum sativum GN=GAPB PE=2 SV=2"	chloroplast	K05298	GAPB; glyceraldehyde-3-phosphate dehydrogenase (NADP+) (phosphorylating) [EC:1.2.1.13]
CSA026796.1	346	C	"Glyceraldehyde-3-phosphate dehydrogenase A, chloroplastic OS=Spinacia oleracea GN=GAPA PE=1 SV=2"	chloroplast	K05298	GAPA; glyceraldehyde-3-phosphate dehydrogenase (NADP+) (phosphorylating) [EC:1.2.1.13]
CSA017654.1	365	C	"Fructose-1,6-bisphosphatase, chloroplastic OS=Pisum sativum GN=FBP PE=1 SV=2"	chloroplast	K03841	FBP; fructose-1,6-bisphosphatase I [EC:3.1.3.11]
CSA015603.1	202	C	"Triosephosphate isomerase, chloroplastic OS=Fragaria ananassa GN=TPI PE=2 SV=1"	chloroplast	K01803	TPI; triosephosphate isomerase (TIM) [EC:5.3.1.1]
CSA019750.1	67	C	"Triosephosphate isomerase, cytosolic OS=Petunia hybrida GN=TPIP1 PE=2 SV=1"	chloroplast	K01803	TPI; triosephosphate isomerase (TIM) [EC:5.3.1.1]
CSA019750.1	165	C	"Triosephosphate isomerase, cytosolic OS=Petunia hybrida GN=TPIP1 PE=2 SV=1"	chloroplast	K01803	TPI; triosephosphate isomerase (TIM) [EC:5.3.1.1]
CSA019750.1	153	C	"Triosephosphate isomerase, cytosolic OS=Petunia hybrida GN=TPIP1 PE=2 SV=1"	chloroplast	K01803	TPI; triosephosphate isomerase (TIM) [EC:5.3.1.1]

Table S6. The information of PPI analysis.

Node name	String name	Protein accession	No. of modification site	Degree	MCODE_ Score	KEGG Gene
GLDP1	AT4G33010.1	CSA015996.1	1	13	8	GLDC; glycine dehydrogenase [EC:1.4.4.2]
GLDP2	AT2G26080.1	CSA020117.1	1	9	8	GLDC; glycine dehydrogenase [EC:1.4.4.2]
GAPA	AT3G26650.1	CSA026796.1	1	22	8.6727273	GAPA; glyceraldehyde-3-phosphate dehydrogenase (NADP+) (phosphorylating) [EC:1.2.1.13]
TIM	AT2G21170.1	CSA015603.1	1	10	7.4181818	TPI; triosephosphate isomerase (TIM) [EC:5.3.1.1]
GAPB	AT1G42970.1	CSA018950.1	2	31	7.125	GAPA; glyceraldehyde-3-phosphate dehydrogenase (NADP+) (phosphorylating) [EC:1.2.1.13]
TPI	AT3G55440.1	CSA019750.1	3	10	7.4181818	TPI; triosephosphate isomerase (TIM) [EC:5.3.1.1]
GAPC1	AT3G04120.1	CSA011668.1	1	9	8	
LOS2	AT2G36530.1	CSA034381.1	1	14	7.4181818	ENO; enolase [EC:4.2.1.11]
AT5G42740	AT5G42740.1	CSA017525.1	1	11	7.4181818	GPI; glucose-6-phosphate isomerase [EC:5.3.1.9]
mtLPD1	AT1G48030.1	CSA010160.1	1	12	8	DLD; dihydrolipoamide dehydrogenase [EC:1.8.1.4]
RCA	AT2G39730.1	CSA003662.1	1	18	7.7142857	
HCEF1	AT3G54050.1	CSA017654.1	1	22	8.1818182	FBP; fructose-1,6-bisphosphatase I [EC:3.1.3.11]
SHM4	AT4G13930.1	CSA009889.1	1	10	8	glyA; glycine hydroxymethyltransferase [EC:2.1.2.1]
GLU1	AT5G04140.2	CSA003654.1	1	17	7	E1.4.7.1; glutamate synthase (ferredoxin) [EC:1.4.7.1]
iPGAM1	AT1G09780.1	CSA033261.1	1	11	7.4181818	
SHM3	AT4G32520.1	CSA013744.1	1	9	8	
LHCA1	AT3G54890.1	CSA024064.1	1	13	8.6727273	LHCA1; light-harvesting complex I chlorophyll a/b binding protein 1
LHCA3	AT1G61520.1	CSA016587.1	1	12	7.1515152	LHCA3; light-harvesting complex I chlorophyll a/b binding protein 3
AT2G45290	AT2G45290.1	CSA012532.1	2	13	7.4181818	E2.2.1.1; transketolase [EC:2.2.1.1]
LHCA2	AT3G61470.1	CSA011328.1	2	14	7.8131868	LHCA2; light-harvesting complex I chlorophyll a/b binding protein 2
AOAT2	AT1G70580.1	CSA024921.1	1	15	8	GGAT; glutamate--glyoxylate aminotransferase [EC:2.6.1.4 2.6.1.2 2.6.1.44]
PSAN	AT5G64040.2	CSA033864.1	2	16	7.9615385	psaN; photosystem I subunit PsaN
PRK	AT1G32060.1	CSA000795.1	3	23	8.1818182	PRK; phosphoribulokinase [EC:2.7.1.19]
LHCB4.2	AT3G08940.2	CSA010862.1	1	10	8	LHCB4; light-harvesting complex II chlorophyll a/b binding protein 4
PSBO1	AT5G66570.1	CSA000627.1	2	17	7.275	psbO; photosystem II oxygen-evolving enhancer protein 1
LHCB5	AT4G10340.1	CSA004532.1	1	13	8.6727273	LHCB5; light-harvesting complex II chlorophyll a/b binding protein 5
PRXQ	AT3G26060.2	CSA010519.1	2	12	7.8222222	
ALDH11A3	AT2G24270.4	CSA034340.1	1	9	8	gapN; glyceraldehyde-3-phosphate dehydrogenase (NADP+) [EC:1.2.1.9]
PETE1	AT1G76100.1	CSA022661.1	1	12	8	petE; plastocyanin
CSP41A	AT3G63140.1	CSA017430.1	1	14	7.8222222	
FNR2	AT1G20020.1	CSA025110.1	1	10	8	petH; ferredoxin--NADP+ reductase [EC:1.18.1.2]
SDH1-1	AT5G66760.1	CSA009205.1	2	6	4	SDHA; succinate dehydrogenase (ubiquinone) flavoprotein subunit [EC:1.3.5.1]
SDH2-2	AT5G40650.1	CSA004395.1	1	6	4	SDHB; succinate dehydrogenase (ubiquinone) iron-sulfur subunit [EC:1.3.5.1]
AT5G65750	AT5G65750.1	CSA032333.1	1	7	4	OGDH; 2-oxoglutarate dehydrogenase E1 component [EC:1.2.4.2]
ACLB-2	AT5G49460.1	CSA034373.1	1	14	5	ACLY; ATP citrate (pro-S)-lyase [EC:2.3.3.8]
mMDH1	AT1G53240.1	CSA034775.1	1	9	4.1666667	MDH2; malate dehydrogenase [EC:1.1.1.37]
c-NAD-MDH2	AT5G43330.1	CSA000242.1	1	8	4.1666667	MDH1; malate dehydrogenase [EC:1.1.1.37]
ATCS	AT2G44350.2	CSA017504.1	1	13	5	CS; citrate synthase [EC:2.3.3.1]
NADP-ME4	AT1G79750.1	CSA009025.1	1	9	4.2857143	E1.1.1.40; malate dehydrogenase (oxaloacetate-decarboxylating)(NADP+) [EC:1.1.1.40]
AT5G58330	AT5G58330.1	CSA017616.1	2	8	4.1666667	E1.1.1.82; malate dehydrogenase (NADP+) [EC:1.1.1.82]
PPC1	AT1G53310.1	CSA032885.1	1	9	5	
FDH	AT5G14780.1	CSA016186.1	2	6	4.7619048	FDH; formate dehydrogenase [EC:1.17.1.9]
AT4G37560	AT4G37560.1	CSA022831.1	1	7	4.7619048	E3.5.1.49; formamidase [EC:3.5.1.49]
P40	AT1G72370.1	CSA020098.1	2	13	3.7333333	RP-SAe; small subunit ribosomal protein SAe
AT4G18100	AT4G18100.1	CSA006911.1	1	5	3.7333333	RP-L32e; large subunit ribosomal protein L32e
AT2G32060	AT2G32060.1	CSA030434.1	1	6	3.7333333	RP-S12e; small subunit ribosomal protein S12e
AT2G09990	AT2G09990.1	CSA036240.1	2	8	3.7333333	RP-S16e; small subunit ribosomal protein S16e
AT4G20980	AT4G20980.1	CSA019651.1	1	4	4	EIF3D; translation initiation factor 3 subunit D
PP2AA2	AT3G25800.1	CSA019797.1	1	4	4	PPP2R1; serine/threonine-protein phosphatase 2A regulatory subunit A
UGP2	AT5G17310.2	CSA029743.1	1	4	3	UGP2; UTP--glucose-1-phosphate uridylyltransferase [EC:2.7.7.9]
UGE5	AT4G10960.1	CSA035914.1	1	3	3	galE; UDP-glucose 4-epimerase [EC:5.1.3.2]
USP	AT5G52560.1	CSA032553.1	1	6	3	USP; UDP-sugar pyrophosphorylase [EC:2.7.7.64]
GME	AT5G28840.1	CSA034927.1	1	3	3	GME; GDP-D-mannose 3', 5'-epimerase [EC:5.1.3.18 5.1.3.-]
Hsp81.4	AT5G56000.1	CSA013811.1	1	5	3	HSP90A; molecular chaperone HtpG
cpHsc70-2	AT5G49910.1	CSA026073.1	1	5	3	HSPA1s; heat shock 70kDa protein 1/2/6/8
Cpn60beta2	AT3G13470.1	CSA036590.1	1	3	3	groEL; chaperonin GroEL
HSP60	AT3G23990.1	CSA006518.1	2	4	3	groEL; chaperonin GroEL
AT1G11860	AT1G11860.1	CSA019996.1	3	17	5.4095238	gcvT; aminomethyltransferase [EC:2.1.2.10]
SBPASE	AT3G55800.1	CSA034492.1	1	18	6.4175824	E3.1.3.37; sedoheptulose-bisphosphatase [EC:3.1.3.37]
PGRL1A	AT4G22890.1	CSA005866.1	1	7	5.7857143	
AT2G32730	AT2G32730.1	CSA030920.1	1	3	3	PSMD1; 26S proteasome regulatory subunit N2
RPT1A	AT1G53750.1	CSA029580.1	1	3	3	PSMC2; 26S proteasome regulatory subunit T1
PAE2	AT3G14290.1	CSA034513.1	1	3	3	PSMA5; 20S proteasome subunit alpha 5 [EC:3.4.25.1]
CAT	AT4G35090.1	CSA035408.1	2	3	2	katE; catalase [EC:1.11.1.6]
ALDH7B4	AT1G54100.1	CSA030116.1	1	4	2	ALDH7A1; aldehyde dehydrogenase family 7 member A1 [EC:1.2.1.31 1.2.1.8 1.2.1.3]
AT5G51970	AT5G51970.1	CSA031477.1	2	3	2	
AT1G09640	AT1G09640.1	CSA003232.1	1	9	2.7	
AT1G32470	AT1G32470.1	CSA029723.1	1	22	5.2666667	gcvH; glycine cleavage system H protein
RBCL	ATCG00490.1	CSA036938.1	3	3	3	rbcL; ribulose-bisphosphate carboxylase large chain [EC:4.1.1.39]
RBCS1A	AT1G67090.1	CSA004763.1	2	10	6	rbcS; ribulose-bisphosphate carboxylase small chain [EC:4.1.1.39]
emb2726	AT4G29060.1	CSA007724.1	1	4	2.7	
SHM1	AT4G37930.1	CSA025613.1	2	24	4.3290043	glyA; glycine hydroxymethyltransferase [EC:2.1.2.1]
At1g07940	AT5G60390.1	CSA001667.1	1	4	3	EEF1A; elongation factor 1-alpha
FBA2	AT4G38970.1	CSA009966.1	3	22	4.2055336	ALDO; fructose-bisphosphate aldolase, class I [EC:4.1.2.13]
AT4G02450	AT4G02450.1	CSA008160.1	1	1	0	
RBCS1B	AT5G38430.1	CSA020587.1	1	4	3	rbcS; ribulose-bisphosphate carboxylase small chain [EC:4.1.1.39]
ATMS1	AT5G17920.1	CSA026696.1	1	5	1.2	metE; 5-methyltetrahydropteroyltriglutamate--homocysteine methyltransferase [EC:2.1.1.14]
HOG1	AT4G13940.1	CSA031781.1	2	3	2	E3.3.1.1; adenosylhomocysteinase [EC:3.3.1.1]
RABE1b	AT4G20360.1	CSA028748.1	2	7	2.7	
GLN1-1	AT5G37600.1	CSA010785.1	1	9	6	glnA; glutamine synthetase [EC:6.3.1.2]
ACO3	AT2G05710.1	CSA025704.1	1	3	3	ACO; aconitate hydratase [EC:4.2.1.3]
At5g06290	AT5G06290.1	CSA018288.1	1	4	2	
TRX-M4	AT3G15360.1	CSA011054.1	1	4	1.4	
SAM1	AT1G02500.1	CSA005033.1	1	2	2	metK; S-adenosylmethionine synthetase [EC:2.5.1.6]
AT1G67280	AT1G67280.1	CSA026411.1	1	2	2	
PSBP-1	AT1G06680.1	CSA033259.1	1	19	6.7058824	psbP; photosystem II oxygen-evolving enhancer protein 2
LOS1	AT1G56070.1	CSA011928.1	1	8	2	
GLX1	AT1G11840.6	CSA011363.1	1	2	2	GLO1; lactoylglutathione lyase [EC:4.4.1.5]
AT4G13780	AT4G13780.1	CSA023566.1	1	4	0.4	MARS; methionyl-tRNA synthetase [EC:6.1.1.10]
AT2G47640	AT3G62840.1	CSA023611.1	1	3	0.5	SNRPD2; small nuclear ribonucleoprotein D2
ROC3	AT2G16600.1	CSA002602.1	1	1	0	
CDSP32	AT1G76080.1	CSA008457.1	1	2	0.6666667	
CI51	AT5G08530.1	CSA015823.1	1	6	1.0666667	NDUFV1; NADH dehydrogenase (ubiquinone) flavoprotein 1 [EC:1.6.5.3 1.6.99.3]
FBA6	AT2G36460.1	CSA035207.1	1	4	3	ALDO; fructose-bisphosphate aldolase, class I [EC:4.1.2.13]
GSTF8	AT2G47730.1	CSA030616.1	2	1	0	GST; glutathione S-transferase [EC:2.5.1.18]
MDAR1	AT3G52880.2	CSA027795.1	1	2	0.6666667	E1.6.5.4; monodehydroascorbate reductase (NADH) [EC:1.6.5.4]
APX1	AT1G07890.1	CSA018282.1	1	1	0	E1.11.1.11; L-ascorbate peroxidase [EC:1.11.1.11]
SUS3	AT4G02280.1	CSA034422.1	1	1	0	E2.4.1.13; sucrose synthase [EC:2.4.1.13]
RGP3	AT3G08900.1	CSA007312.1	1	1	0	RGP; reversibly glycosylated polypeptide / UDP-arabinopyranose mutase [EC:2.4.1.- 5.4.99.30]
RGP1	AT3G02230.1	CSA017438.1	1	1	0	RGP; reversibly glycosylated polypeptide / UDP-arabinopyranose mutase [EC:2.4.1.- 5.4.99.30]
PB	ATCG00480.1	CSA036932.1	1	5	2	
AVP1	AT1G15690.1	CSA000036.1	1	2	0.6666667	ppa; inorganic pyrophosphatase [EC:3.6.1.1]
AT5G08680	AT5G08680.1	CSA033994.1	1	5	2	ATPeF1B; F-type H+-transporting ATPase subunit beta [EC:3.6.3.14]
UXS6	AT2G28760.1	CSA031127.1	1	1	0	UXS1; UDP-glucuronate decarboxylase [EC:4.1.1.35]
TCP-1	AT3G20050.1	CSA035465.1	1	5	1.2	
CLPC1	AT5G50920.1	CSA016043.1	1	2	0.6666667	clpC; ATP-dependent Clp protease ATP-binding subunit ClpC
AT1G51980	AT1G51980.1	CSA009282.1	1	3	1.6666667	
PKT3	AT2G33150.1	CSA035327.1	1	2	2	ACAA1; acetyl-CoA acyltransferase 1 [EC:2.3.1.16]
DELTA-OAT	AT5G46180.1	CSA028285.1	1	2	0.6666667	rocD; ornithine--oxo-acid transaminase [EC:2.6.1.13]
AT3G58610	AT3G58610.1	CSA003086.1	1	2	2	ilvC; ketol-acid reductoisomerase [EC:1.1.1.86]
ABA1	AT5G67030.1	CSA014226.1	1	3	0.5	ZEP; zeaxanthin epoxidase [EC:1.14.15.21]
STN7	AT1G68830.1	CSA015987.1	1	1	0	
AT5G26830	AT5G26830.1	CSA018664.1	1	4	0.4	
CA1	AT3G01500.2	CSA002108.1	1	5	3	cynT; carbonic anhydrase [EC:4.2.1.1]
DAP	AT4G33680.1	CSA004187.1	1	2	0.6666667	E2.6.1.83; LL-diaminopimelate aminotransferase [EC:2.6.1.83]
AT2G42220	AT2G42220.1	CSA003818.1	1	6	3.4285714	
LAP2	AT4G30920.1	CSA005660.1	1	3	3	CARP; leucyl aminopeptidase [EC:3.4.11.1]
IIL1	AT4G13430.1	CSA008456.1	1	3	3	
AT4G13010	AT4G13010.1	CSA001213.1	1	1	0	
SULA	AT2G21280.1	CSA021957.1	1	3	3	
AT1G29880	AT1G29880.1	CSA008625.1	1	2	0.6666667	
AT4G10300	AT4G10300.1	CSA004535.1	1	1	0	
XK-2	AT5G49650.1	CSA022347.1	1	1	0	xylB; xylulokinase [EC:2.7.1.17]
AQI	AT4G38220.2	CSA003990.1	1	1	0	ACY1; aminoacylase [EC:3.5.1.14]
